# Aggregation-Induced Catalysis: Asymmetric Catalysis with Chiral Aggregates

**DOI:** 10.34133/research.0163

**Published:** 2023-06-09

**Authors:** Yao Tang, Yu Wang, Qingkai Yuan, Sai Zhang, Jia-Yin Wang, Shengzhou Jin, Ting Xu, Junyi Pan, Kazimierz Surowiec, Guigen Li

**Affiliations:** ^1^ Department of Chemistry and Biochemistry, Texas Tech University, Lubbock, TX 79409-1061, USA.; ^2^ School of Chemistry and Chemical Engineering, Nanjing University, Nanjing 210093, China.; ^3^ School of Pharmacy, Continuous Flow Engineering Laboratory of National Petroleum and Chemical Industry, Changzhou University, Changzhou, Jiangsu 213164, China.

## Abstract

So far, there have been 4 methods to control chirality including the use of chiral auxiliaries, reagents, solvents, and catalysts documented in literature and textbooks. Among them, asymmetric catalysts are normally divided into homogeneous and heterogeneous catalysis. In this report, we present a new type of asymmetric control—asymmetric catalysis via chiral aggregates that would not belong to the above categories. This new strategy is represented by catalytic asymmetric dihydroxylation reaction of olefins in which chiral ligands are aggregated by taking advantage of typical aggregation-induced emission systems containing tetrahydrofuran and H_2_O cosolvents. It was proven that the chiral induction can be enhanced from er of 78:22 to 97:3 simply by changing the ratios of these 2 cosolvents. The formation of chiral aggregates of asymmetric dihydroxylation ligands, (DHQD)_2_PHAL and (DHQ)_2_PHAL, has been proven by aggregation-induced emission and a new analytical tool—aggregation-induced polarization established by our laboratory. In the meanwhile, chiral aggregates were found to be formed either by adding NaCl into tetrahydrofuran/H_2_O systems or by increasing concentrations of chiral ligands. The present strategy also showed promising reverse control of enantioselectivity in the Diels–Alder reaction. This work is anticipated to be extended broadly to general catalysis, especially to asymmetric catalysis in the future.

## Introduction

Over the past half a century, asymmetric synthesis and catalysis have been actively pursued in chemical and materials sciences [1–4]. Increasing numbers of drugs and pesticides contain chiral structural units in their structures since drug actions require conformational matching to increase their potency and selectivity toward receptors and other active targets inside and on the surfaces of cells [5–9]. Structural design of pharmaceuticals plays key role in reducing or avoiding severe side effects during their action processes [6]. In the meanwhile, more advanced materials, especially nano- and photoelectronic materials contain micro- and macrochiral units requiring various levels of asymmetric factors [10–16]. Therefore, the control of chirality will continue to play a crucial role in the aforementioned fields. So far, 4 synthetic strategies have been developed for controlling molecular chirality relying on the use of chiral catalysts (bio- and chemocatalysts), chiral auxiliaries, chiral reagents, and chiral solvents [17–27]. There have been no other asymmetric controllers beyond these 4 methods documented in literature so far.

**Fig. 1.**
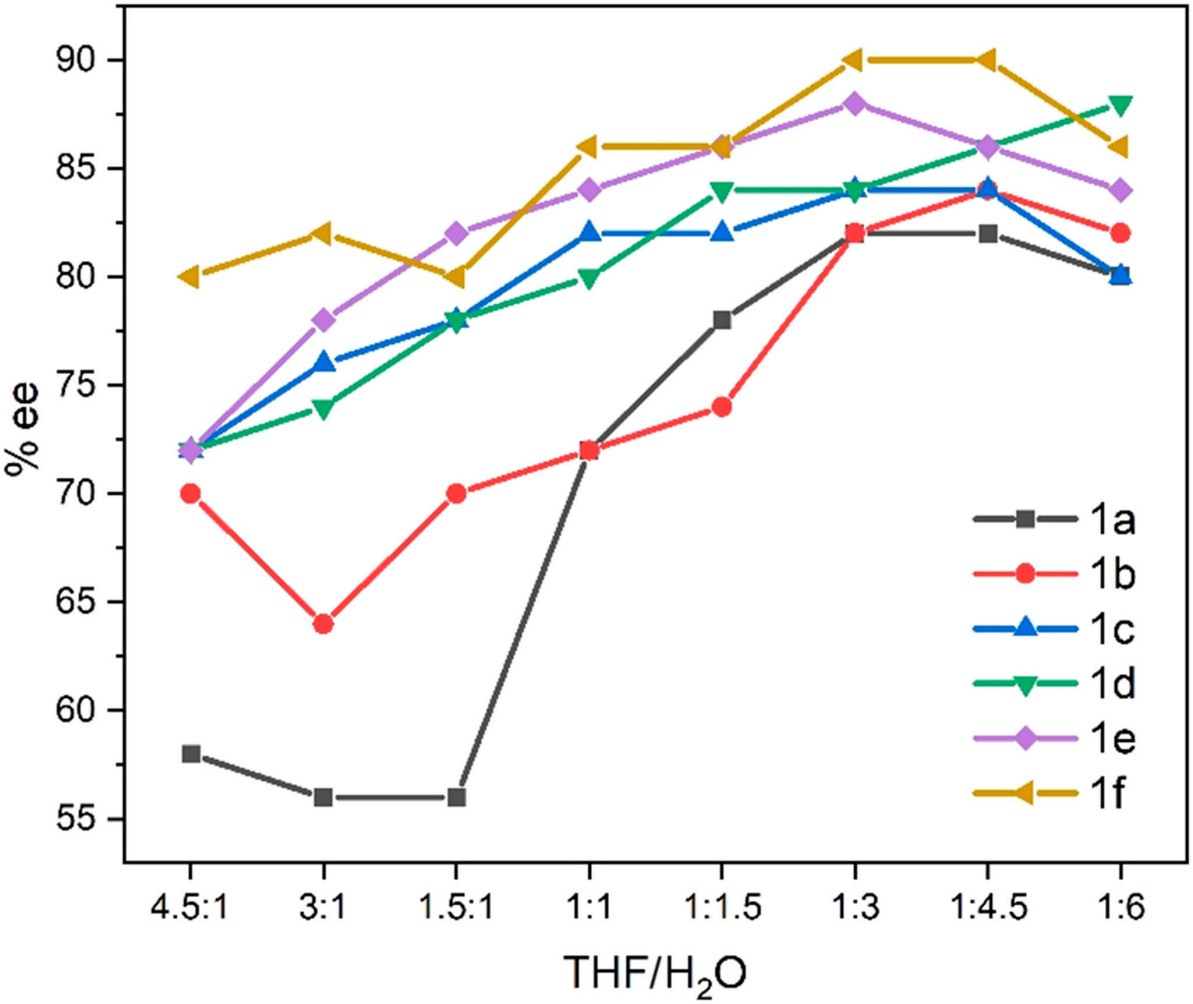
Curve description of er enhancement in aggregation-induced catalysis from AD-mix-β.

Very recently, our laboratories have developed a new tool, stoichiometric aggregation-induced asymmetric synthesis (AIAS), providing asymmetric access to chiral amino products by taking advantage of chiral aggregates of group-assisted purification reagents, *N*-phosphonyl imines (Fig. 1) [28–30]. The asymmetric group-assisted purification synthesis of functionalized 2,3-dihydrobenzofurans by reacting salicyl *N*-phosphonyl imines with dialkyl bromomalonates was conducted in various typical cosolvent systems of aggregation [28]. By changing the ratios of cosolvents of tetrahydrofuran (THF) and ethanol, the chiral products were controlled toward different directions, i.e., (*R*)-isomeric products were predominantly formed over (*S*)-counterparts when amounts of ethanol were increased, and vice versa. The formation of chiral aggregates was confirmed by aggregation-induced emission (AIE) [31–33] and, concurrently, by a new analytical tool—aggregation-induced polarization (AIP), which was established by our laboratory [28,34–35]. This work would change the traditional thoughts on organic synthesis in which individual molecules of starting materials are involved for forming products, i.e., organic reactions and synthesis are about the behaviors of freely distributed single molecules in solutions. The concentrations of reactants are based on the above stoichiometric calculations. However, the AIAS work proved that organic reactions would involve a combination of individual molecules, their dimers, trimers, etc., instead of only individual molecules in reaction systems. In particular, the reactions would consist of a series of chiral aggregates, which can increase the enantioselective control of asymmetric reactions, and even direct reactions toward different directions asymmetrically. The formations of achiral aggregates (for normal reactions) and chiral aggregates (for asymmetric reactions) during reaction processes would depend on various factors, such as concentrations, solvents/cosolvents, temperature, additives, pressure, etc. The opposite control provides a unique tool to control the chirality of organic and medicinal building blocks without changing the chirality of starting materials and catalysts. Therefore, this work belongs to greener and more environmentally friendly synthesis and production. By simply changing inexpensive solvents, stereoselectivity enhancement and opposite stereoisomers can be conveniently achieved. The latter avoids the tedious preparation of starting materials of opposite chirality and minimizes manpower, energy usage, and waste generation.

Inspired by our previous work on stoichiometric AIAS, our next effort is to establish asymmetric catalysis by taking advantage of chiral aggregates. One of the most successful asymmetric catalytic reactions, asymmetric dihydroxylation (AD) of alkenes, was thus selected for this purpose. In this report, we would like to disclose our preliminary results of this investigation by conducting AD under a series of new aggregation conditions.

## Results

### Rationale and synthesis

We began this work using one of the most influential asymmetric catalytic reactions—the Sharpless catalytic AD reaction owing to its simplicity with predictable asymmetric control by well-known and commercially available chiral ligands [36–38]. The commercial AD mixes containing the ligands (DHQ)_2_PHAL and (DHQD)_2_PHAL, referred to as AD-mix-α and AD-mix-β, respectively, were directly used for the reaction at room temperature. For the sake of aggregation, the cosolvents of THF–H_2_O commonly adopted in AIE [31–33] and AIP [34–35] were used for the present study instead of the traditional AD solvents of acetone, acetonitrile, and alcohols together with water. Among many AD substrates, styrene and its derivatives were considered since they are best researched mechanistically in AD due to their simplicity.

The reactions were carried out in vials equipped with magnetic stirrers and 2.0 or 6.0 ml of cosolvents, as described in the literature [38]. Styrene (0.20 or 0.60 mmol) and AD-mix-α or AD-mix-β (0.28 or 0.84 g) were added to each vial. The resulting mixtures were stirred at room temperature for 24 h until starting materials were consumed, as revealed by thin-layer chromatography. The reactions were quenched by adding sodium sulfite and then stirred at room temperature for 1 h. The resulting mixtures were then extracted several times with ethyl acetate. The organic layers were combined, dried over anhydrous Na_2_SO_4_, and concentrated to give a mixture of a crude diol containing chiral ligand, which was subsequently purified through flash chromatography prior to chiral high-performance liquid chromatography (HPLC) analysis.

The relationship between solvent ratios and enantioselectivity was researched by setting them toward 2 directions with the ratios of THF and H_2_O on the *x* horizontal coordinate correlating to enantioselectivity on the *y* vertical ordinate. Following our previous manner, the ratio of v/v = 1:1 was first set in the middle for various reactions to obtain average data as the reference on the *x* horizontal coordinate. The reaction was performed in 9 sets of cosolvents consisting of THF/ H_2_O: 4.5:1, 3:1, 1.5:1, 1:1, 1:1.5, 1:3, 1:4.5, and 1:6 (Fig. 1 and Table 1).

**Table 1.** Primary results of aggregation-induced catalytic AD reaction.^a^

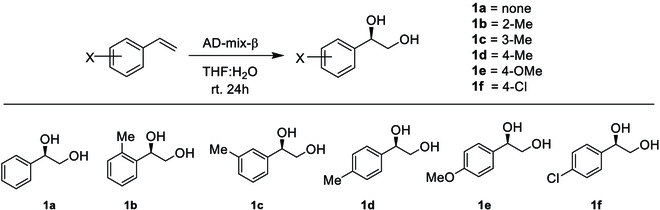												
EntryTHF/H_2_O	1a		1b		1c		1d		1e		1f^b^	
	R:S	Yield^c^	R:S^d^	Yield	R:S	Yield	R:S	Yield	R:S	Yield	R:S	Yield
4.5:1	**79**:21	99%	**85**:15	82%	**86**:14	68%	**86**:14	99%	**86**:14	78%	**90**:10	45%
3:1	**78**:22	99%	**82**:18	82%	**88**:12	98%	**87:**13	99%	**89**:11	78%	**91**:9	53%
1.5:1	**78**:22	99%	**85**:15	84%	**89**:11	99%	**89**:11	99%	**91**:9	84%	**90**:10	54%
1:1	**86**:14	99%	**86**:14	85%	**91**:9	94%	**90**:10	99%	**92**:8	84%	**93**:7	50%
1:1.5	**89:**11	99%	**87**:13	80%	**91**:9	94%	**92**:8	99%	**93**:7	86%	**93**:7	48%
1:3	**91**:9	99%	**91**:9	86%	**92**:8	99%	**92**:8	99%	**94**:6	88%	**95**:5	50%
1:4.5	**91**:9	99%	**92**:8	80%	**92**:8	99%	**93**:7	99%	**93**:7	86%	**95**:5	50%
1:6	**90**:10	97%	**91:**9	84%	**90**:10	95%	**94**:6	99%	**92**:8	84%	**93**:7	58%

^a^Standard condition: Styrene (0.2 mmol) and 0.28 g of AD-mix-β in 2 ml of THF/H_2_O mixture at room temperature for 24 h. ^b^Condition: Styrene (0.6 mmol) and 0.84 g of AD-mix-β in 6 ml of THF/H_2_O mixture at room temperature for 24 h. ^c^All yields determined by crude ^1^H NMR with internal standard. ^d^All R:S ratios determined by HPLC analysis of isolated samples.

To avoid or minimize possible variations of experimental skills, especially purifications of column chromatography, we determined the yield of each trail by crude proton nuclear magnetic resonance (NMR) spectroscopic analysis using CH_2_Br_2_ as the internal standard. As described in Table 1, each trial of styrene-**1a** produced a high yield that was nearly unaffected by the ratios of solvents. For the case of styrene-**1b**, enantioselectivity is slightly higher than that of styrene-**1a**, albeit yields drop ~10% as compared with the latter. This observation would be attributed to the bigger steric effect by *o*-Me group on the phenyl ring when the ligand–metal complex approaches this substrate. Cases of styrene-**1c** and -**1d** showed similar enantioselectivity and yields, except for the first trial of **1c**, giving a lower yield. As the fraction of H_2_O was increased as the major solvent in the cosolvent systems, enantioselectivity was increased in the above cases in general. For the case of styrene-**1e** attached by a strong electron-donating group (*p*-OMe), chemical yields dropped again, but enantioselectivity was gradually increased as *fw* became bigger. A similar trend was observed for styrene-**1f** in regard to enantioselectivity, but chemical yields were substantially diminished to a range from 45% to 58%.

More specifically, for case **1a**, the largest jump of er occurred from S:R = 78:22 to 86:14 when v-THF/v-H_2_O was increased from 1.5:1 to 1:1, which eventually reached 91:9 when the v-THF/v-H_2_O ratio was set within a range of 1:3 to 1:6 as shown in both Table 1 and Fig. 1. For case **1b**, there is not an obvious er jump depicted, but a steady er enhancement was observed as H_2_O became more predominant in the reaction mixture within the whole range of v-THF/v-H_2_O ratios. A similar situation occurred for the rest of other cases, **1c** to **1f**, although er dropped a little for most of these cases when the v-THF/v-H_2_O ratio reached 1:6. The present phenomenon would be attributed to the aggregate formation from (DHQD)_2_PHAL-based catalyst species, the higher the *fw* of cosolvents, the more aggregation for the asymmetric catalysis. It seems that electronic effects of styrene substrates did not show obvious effects on er enhancement trend when substitutes of Me, OMe, and Cl were attached to the phenyl ring, although electron-withdrawing Cl moiety led to low chemical yields than in other cases.

**Fig. 2.**
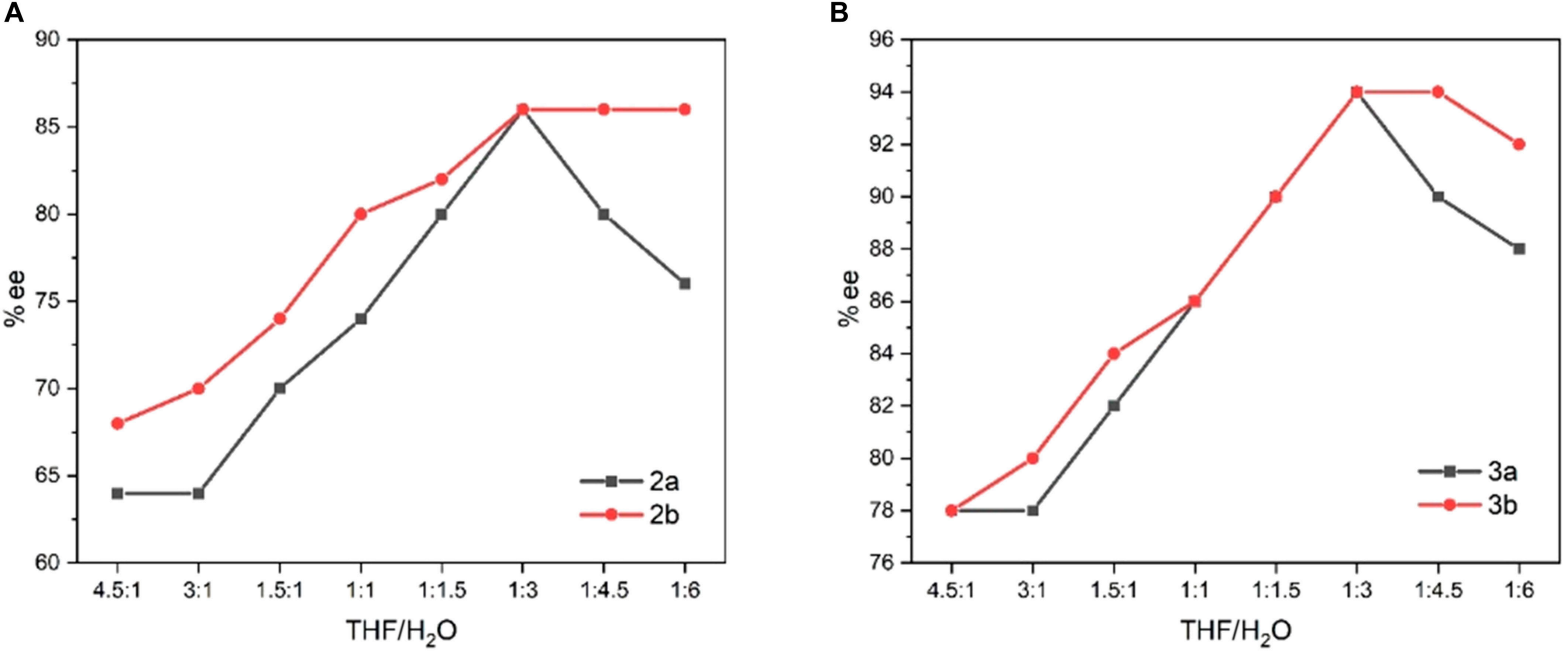
Curve descriptions of er enhancement in aggregation-induced catalysis via (A) AD-mix-α and (B) *trans*-β-substituted styrenes via AD-mix-β.

Since (DHQD)_2_PHAL and (DHQ)_2_PHAL are not exactly defined as enantiomers considering Et attachment on C(sp^3^) chiral centers on their tricyclic frames, AD-mix-α was next examined for the present aggregation-induced asymmetric catalysis. Two representative substrates, styrene, and 4-Me–styrene were selected for this purpose. As described in Table 2 and Fig. 2, both cases showed steady enantioselectivity enhancement as H_2_O became more predominant in the AD reaction mixture within the whole range of v-THF/v-H_2_O ratios, affording er from R:S = 18:82 to 12:88 and 16:84 to 7:93, respectively. However, er dropped back a little in the case of **2a** after the v-THF/v-H_2_O ratio turned from 1:3. Both **1a** and **1b** cases afforded consistent chemical yields from 81% to 89% and 90% to 96%, respectively, within the whole range of v-THF/v-H_2_O ratios.

**Table 2.** Aggregation-induced catalytic AD based on (DHQ)_2_PHAL (2a and 2b) and on *trans*-styrene (3a and 3b).

	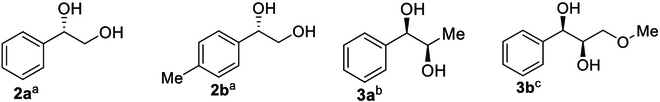							
THF/H_2_O	R:S^d^	Yield^e^	R:S	Yield	RR:SS	Yield	RR:SS	Yield
4.5:1	18:**82**	81%	16:**84**	90%	**89**:11	52%	**89:**11	50%
3:1	18:**82**	86%	15:**85**	95%	**89**:11	72%	**90**:10	88%
1.5:1	15:**85**	86%	13:**87**	93%	**91**:9	73%	**92**:8	93%
1:1	13:**87**	85%	10:**90**	93%	**93**:7	73%	**93**:7	90%
1:1.5	10:**90**	83%	9:**91**	93%	**95**:5	74%	**95**:5	96%
1:3	7:**93**	87%	7:**93**	96%	**97**:3	72%	**97**:3	90%
1:4.5	10:**90**	89%	7:**93**	95%	**95**:5	74%	**97**:3	87%
1:6	12:**88**	89%	7:**93**	96%	**94**:6	70%	**96**:4	87%

^a^Condition: Styrene (0.2 mmol) and 0.28 g of AD-mix-α in 2 ml of THF/H_2_O mixture at room temperature for 24 h. ^b^Condition 3a: Styrene (0.6 mmol) and 0.84 g of AD-mix-β in 6 ml of THF/H_2_O mixture at room temperature for 24 h. ^c^Condition: Styrene (0.2 mmol) and 0.28 g of AD-mix-β in 2 ml of THF/H_2_O mixture at room temperature for 24 h. ^d^ All R:S and RR:SS ratios were determined by chiral HPLC analysis of isolated samples. ^e^All yields determined by crude ^1^H NMR with internal standard.

The present aggregation-induced asymmetric catalysis was also proven to be effective for *trans*-β-substituted styrenes, which were examined under the standard AD condition. As revealed in Table 2 and Fig. 2, the ee values in **3a** case with methyl substitution on β position boosted from 78% to 94% from the ratio of v-THF/v-H_2_O = 6:1 to 1:3, but as the proportion of water in the combination was increased, the ee value decreased somewhat to 88%. Notably, the **3n**-diol with a methyl ester on the β position of styrene can only give 70% ee with a cosolvent ratio of 6:1 (v-THF/v-H_2_O). However, as the proportion of water in the solvent system grew, the ee value reached as high as 94% and dropped slightly to 92% when the ratio hit 1:6 (v-THF/v-H_2_O).

### Aggregation investigation on chiral ligands—AIE and AIP

To confirm the existence of the aggregates from chiral quinine ligands during the catalytic pathway, we investigated the aggregation of (DHQD)_2_PHAL and (DHQ)_2_PHAL via ultraviolet-visible (UV-vis) spectroscopy and photoluminescence (PL) spectroscopy. To conduct the UV-vis analysis of (DHQD)_2_PHAL and (DHQ)_2_PHAL, we dissolved 2 chiral ligands in THF at a concentration of *c* = 25 μg/ml. Two chiral ligands exhibited nearly identical spectral behaviors featuring maximum absorptions at around 290, 320, and 334 nm (Fig. 6A). THF and water, the cosolvents used in the asymmetric catalytic step, were now chosen to study both ligands’ AIE properties. Figure 6B and C shows that (DHQD)_2_PHAL and (DHQ)_2_PHAL exhibited aggregation phenomena by adjusting the irradiation power to 700 V and excitation wavelengths to 350 nm. Moreover, these 2 ligands demonstrated a similar AIE property, as their emission intensity grew progressively from 50 to 400 when the water fraction was changed from 0% to 50%. Interestingly, the emission maxima of (DHQD)_2_PHAL jumped dramatically to around 780 as another 10% water fraction was added. Compared with the (DHQD)_2_PHAL, the emission maxima of (DHQ)_2_PHAL were only raised to about 500.

Next, we investigated whether the polarity of the protonic component in the cosolvent can enhance the fluorescence intensity of chiral ligands. Both ligands with a 50% water fraction were chosen as the model for this experiment, which was inputted the same excitation wavelength and voltage for excitation as that in AIE studies. Figure 6D shows that the PL intensities of (DHQ)_2_PHAL were increased slightly as the protonic component became polar. The PL intensities of (DHQD)_2_PHAL featured a more robust growth according to the NaCl concentration. As the NaCl concentration in the pure solvent mixture rose to 0.05 g/100 ml, the intensity climbed from 360 to 386. Finally, the intensity reached 403 as the NaCl concentration was doubled. We believe that this observation has a marked potential for AIE enhancement.

**Fig. 3.**
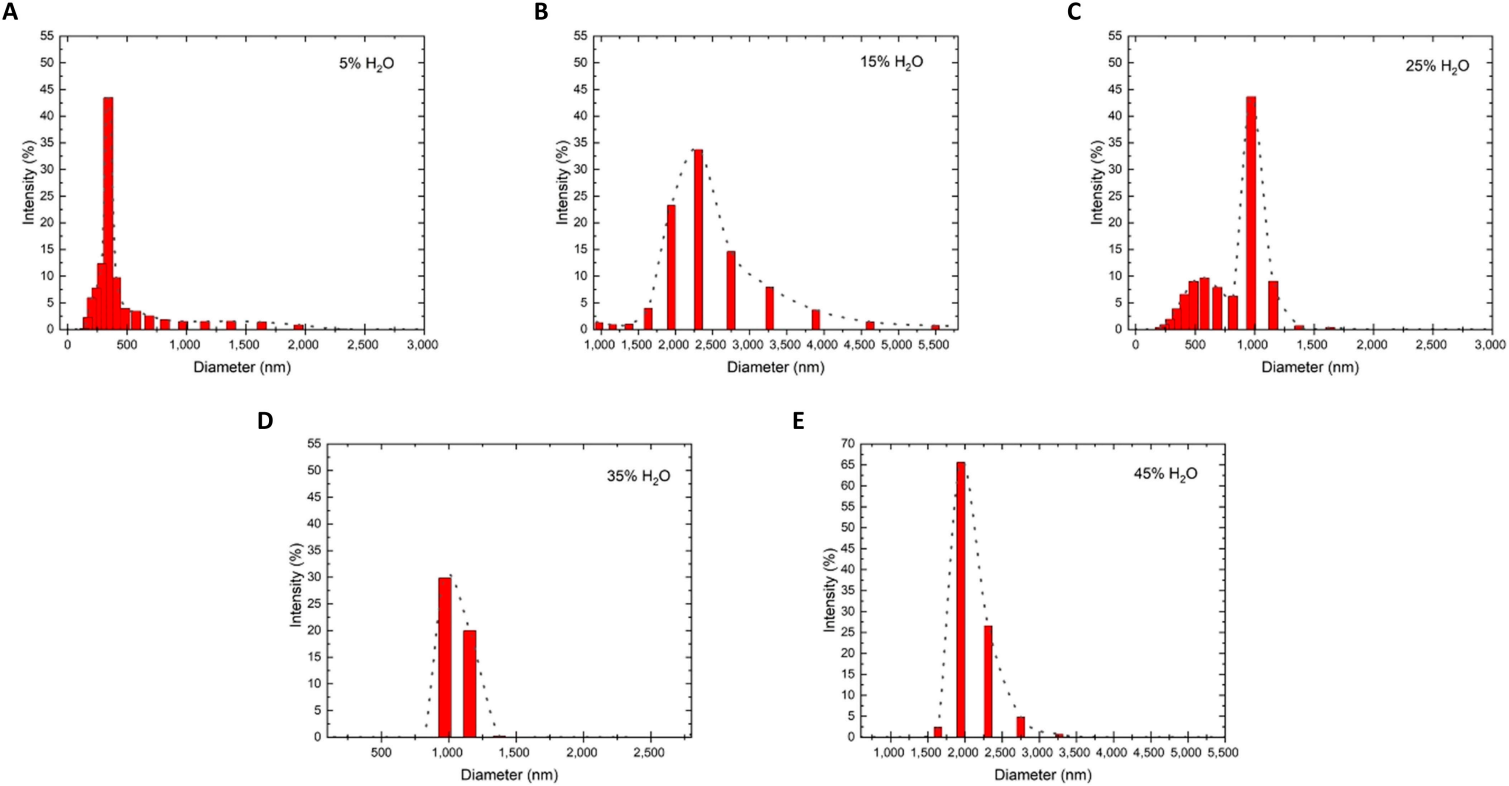
DLS particle size distribution curves of AD-β obtained in THF/water mixture (5% to 45% H_2_O, v/v).

**Fig. 4.**
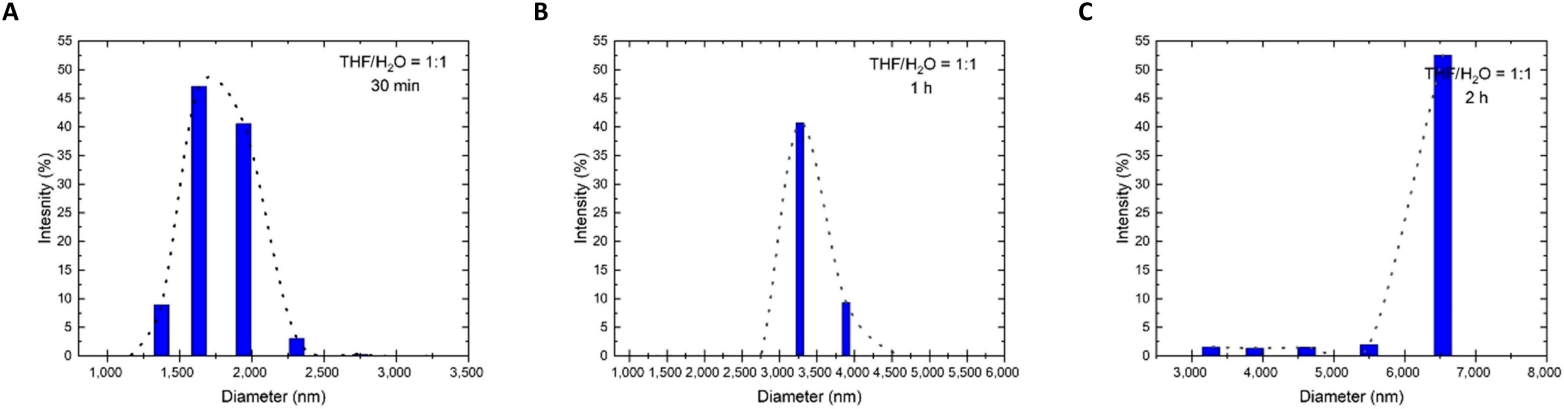
DLS particle size distribution curves of AD-β obtained in THF/water mixture (1:1 THF/H_2_O) at times of 30 min (A), 1 h (B), and 2 h (C).

Dynamic light scattering (DLS) experiments show that the size of aggregates from AD-β increased with water fraction rising from 5% to 45%, which matched the results of the emission study (Fig. 3). This result also demonstrated that increasing polarity affects the size distribution, increasing from 344 to 1,944 nm. In addition, the largest diameter size of the aggregates jumped from 1,635 to 6,540 nm in the 1:1 THF/water mixture as time went to 2 h, suggesting that the aggregate particles formed gradually (Fig. 4). This result supported that the molecular aggregation was ongoing during the dihydroxylation reaction in favor of enantioselective enhancement.

**Fig. 5.**
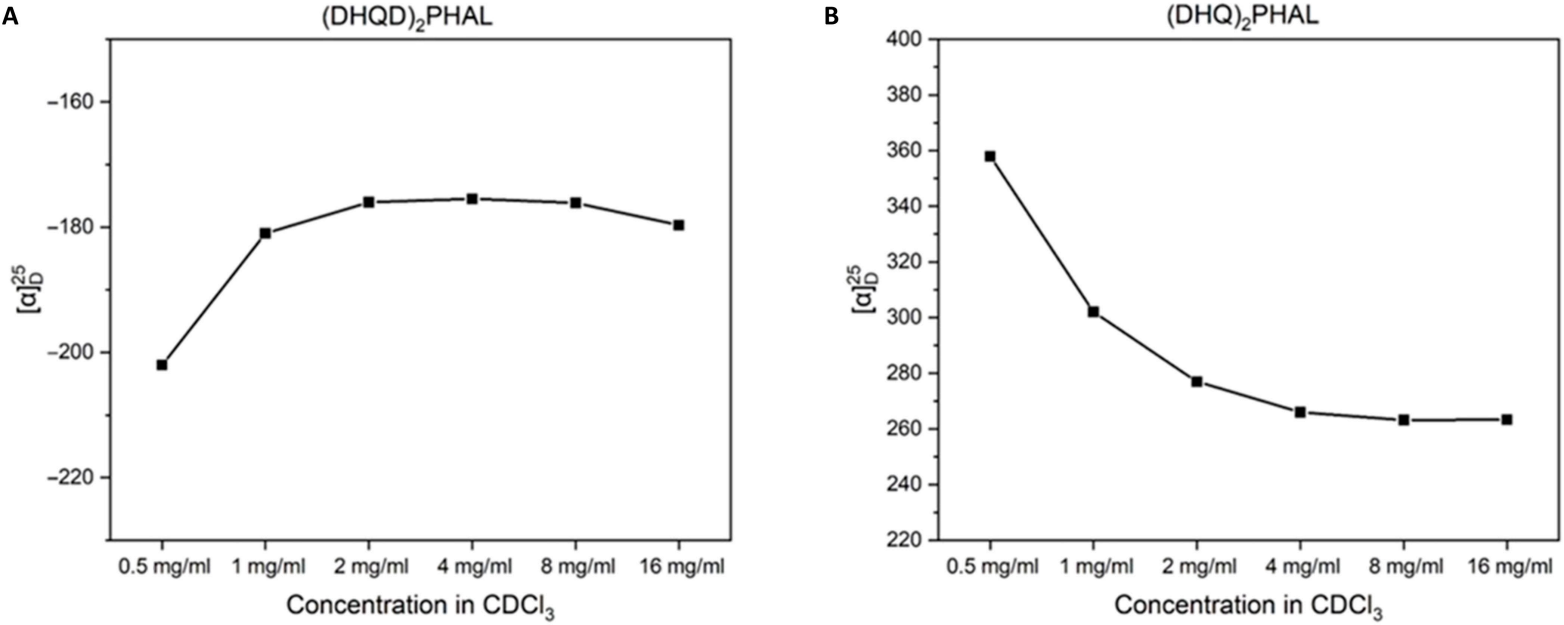
Correlation between specific optical rotation and sample concentrations.

The aggregation study was further extended by investigating the correlation between the specific optical rotation of chiral ligands’concentrations. As shown in Fig. 5, the specific rotation of (DHQD)_2_PHAL surged from −202° to −176° according to the concentration climbed from 0.5 to 2.0 mg/ml and then continuously increased to −179° as the sample was concentrated to 16 mg/ml. On the contrary, the specific rotation of (DHQ)_2_PHAL was decreased significantly from 358° to 266° as the concentration was increased from 0.5 to 4.0 mg/ml and, eventually, was remained constant at 263° as the concentration reached 16.0 mg/ml.

**Fig. 6.**
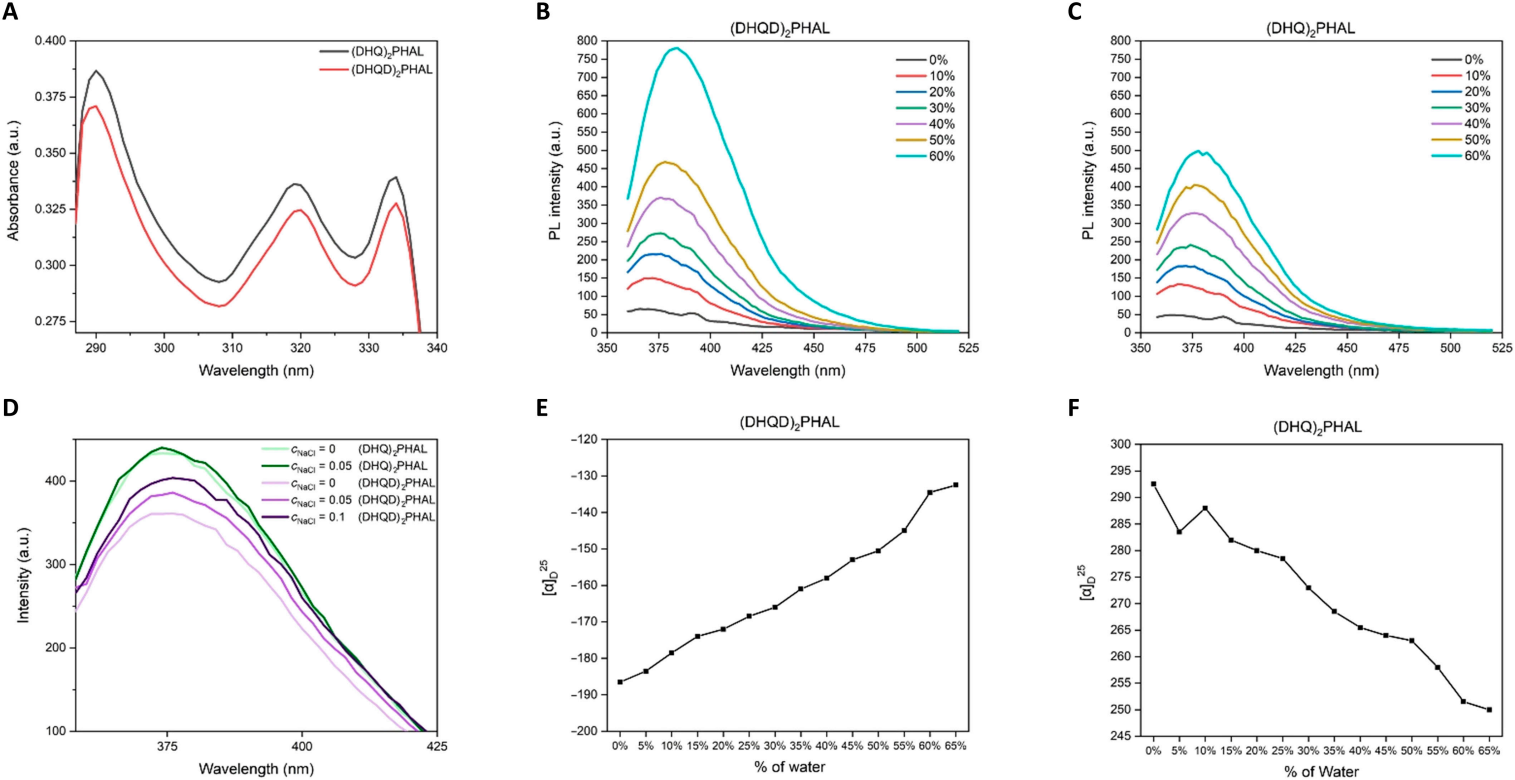
(A) UV-vis absorption of (DHQD)_2_PHAL and (DHQ)_2_PHAL in THF; *c* = 25 μg/ml PL spectra of (DHQD)_2_PHAL (B) and (DHQ)_2_PHAL (C) in THF/water mixtures with different water fractions (*fw*); *c* = 0.1 mM; *λex* = 350 nm. (D) PL spectra of (DHQD)_2_PHAL and (DHQ)_2_PHAL in THF/water mixtures at 50% water fraction with different concentrations of NaCl; *c*_chiral ligands_ = 0.1 ml, *c*_NaCl_ = g/100 ml; *λex* = 350 nm AIP of (DHQD)_2_PHAL (E) and (DHQ)_2_PHAL (F) in THF/water cosolvents; *c* = 2 mg/ml. a.u., arbitrary units.

The correlation of chiral aggregates to AIP was further explored to render optical rotation enhancement or adjustment as more polar solvent was added in cosolvents [34,35]. AIP measurements of chiral dimeric quinine ligands were conducted using a Rudolph polarimeter (Rudoph Research Analytica APIV/2W) with the sodium lamp emitting light at 589 nm as the light source. The samples were prepared at a consistent concentration (*c* = 2 mg/ml) in the cosolvents commonly used AIE and, for the present AD, were thus utilized. The average specific rotation values taken from 3 measurements were considered the most authentic results and further plotted to relation curves with specific rotation on the *y* axis. The water fraction was set at the component of 5% (v/v) on the *x* horizontal axis. As shown in Fig. 6E and F, (DHQD)_2_PHAL demonstrated a substantial increase trend in the specific optical rotation values with gradually increased water fractions, from −186.5° to −132.5° during the water fraction’s changing from 0% to 65%. In contrast, (DHQ)_2_PHAL showed an opposite trend, which featured a consistent descending trend, in which the optical rotation value dramatically fell from 292.5° to 250°.

Last but not least, the present aggregation-induced asymmetric catalysis strategy demonstrated promising results on other reactions. For example, in the same cosolvent system containing THF and H_2_O, organocatalytic asymmetric Diels–Alder reaction between 1,3-diphenylisobenzofuran and (*E*)-but-2-enal depicted opposite enantioselectivity with respect to *endo*-isomer by increasing *fw* values. The exclusive *endo*/*exo* selectivity was observed under this aggregation system that is different from the reported counterpart using the same catalyst of (*S*)-5-benzyl-2,2,3-trimethylimidazolidin-4-one (Fig. S17) [38–41].

## Discussion

In summary, we have established aggregation-induced asymmetric catalysis as a new tool providing access to chiral products. Enantioselectivity enhancement was witnessed by the Sharpless catalytic AD of alkenes by changing the ratios of cosolvents of THF and H_2_O. Through the formation of chiral aggregates, the chirality of products can even be reversed, which is extremely challenging for classical tools to achieve. The formation of chiral aggregates has been proven by AIE and, concurrently, by AIP of chiral ligands in typical AIE cosolvent systems. Aggregation was also investigated by changing the concentration of chiral ligands or by adding NaCl into the solution for PL enhancement. Together with our previous stoichiometric AIAS, which afforded more notable opposite control of chirality simply by changing ratios of THF and H_2_O, the present work is anticipated to further find more academic and industrial applications in asymmetric synthesis. We believe that the formation of aggregates from starting materials, intermediates, catalytic species, and products in organic and aqueous media would be quite general, especially when the above 4 categories of aggregates coexist during the reaction processes. This work also indicates that the biological catalysis in water systems via bioaggregates (e.g., enzyme aggregates) would be common and is worth further investigation. It will play an increasingly important role in research on biocatalysis, greener and more environmentally friendly synthesis and materials in the future.

## Materials and Methods

Unless otherwise stated, all reactions were magnetically stirred and conducted in oven-dried glassware in anhydrous solvents under Ar, applying standard Schlenk techniques. Solvents and liquid reagents, as well as solutions of solid or liquid reagents, were added via syringes, stainless steel, or polyethylene cannulas through rubber septa or through a weak Ar counterflow. Solvents were removed under reduced pressure at 40 to 65 °C using a rotavapor. All given yields are isolated yields of chromatographically and NMR spectroscopically materials. Internal standard was performed for yield measurements as well. All commercially available chemicals were used as received without further purification. Solvents were obtained as follows: THF is delivered from an Innovation Technology solvent system.

The ^1^H and ^13^C NMR spectra were recorded in CDCl_3_ or dimethyl sulfoxide (DMSO)–*d*_6_ on 400- and 500-MHz instruments with TMS as internal standard. For referencing of the ^1^H NMR spectra, the residual solvent signal (δ = 7.26 for CDCl_3_ and δ = 2.50 for DMSO-*d*_6_) were used. In the case of the ^13^C NMR spectra, the signal of solvents (δ = 7.16 for CDCl_3_ and δ = 39.52 for DMSO-*d*_6_) were used. Chemical shifts (δ) were reported in ppm with respect to TMS. Data are represented as follows: chemical shift, multiplicity (s = singlet, d = doublet, t = triplet, and m = multiplet), coupling constant (*J*, Hz), and integration. Fluorescence data were collected using the Cary Eclipse Fluorescence Spectrophotometer and Eclipse ADL program. Measurements were performed with diluted samples with 0.1 mM concentrations at 400-nm maximum excitation wavelength under electric power at 900 V. Optical rotations were measured with a Rudolph Research Analytical APIV/2W Polarimeter at the indicated temperature with a sodium lamp. Measurements were performed in a 2-ml tube with the concentration unit of grams per 100 ml in the corresponding solvents. AIP measurements of chiral dimeric quinine ligands was conducted using a Rudolph polarimeter (Rudoph Research Analytica APIV/2W) with the sodium lamp emitting light at 589 nm as the light source.

## Data Availability

All data are available in the manuscript or the Supplementary Materials.

## Supplementary Materials

Supplementary 1 General procedure of asymmetric catalytic AD and Diels–Alder reactions and analytical data, ^1^H NMR spectra, ^13^C NMR spectra, and chiral HPLC of resulting products Fig. S1. ^1^H NMR Spectra of 1a. Fig. S2. ^13^C NMR Spectra of 1a. Fig. S3. ^1^H NMR Spectra of 1b. Fig. S4. ^13^C NMR Spectra of 1b. Fig. S5. ^1^H NMR Spectra of 1c. Fig. S6. ^13^C NMR Spectra of 1c. Fig. S7. ^1^H NMR Spectra of 1d. Fig. S8. ^13^C NMR Spectra of 1d. Fig. S9. ^1^H NMR Spectra of 1e. Fig. S10. ^13^C NMR Spectra of 1e. Fig. S11. ^1^H NMR Spectra of 3a. Fig. S12. ^13^C NMR Spectra of 3a. Fig. S13. ^1^H NMR Spectra of 3b. Fig. S14. ^13^C NMR Spectra of 3b. Fig. S15. DLS particle size distribution curves of AD-β obtained in THF/Water mixture (5-5 45% H2O, v/v). Fig. S16. DLS particle size distribution curves of AD-β obtained in THF/Water mixture (1:1 THF: H2O) at times of 30 min, 1 hour, and 2 hours. Fig. S17. Aggregation-induced catalysis on organocatalytic asymmetric Diels-Alder reaction. References [*39*–*41*]
