# Supplementary material for: Aggregation-Induced Catalysis: Asymmetric Catalysis with Chiral Aggregates
Source: Research (Wash D C). 2023 Jun 9;6:0163. doi: 10.34133/research.0163 (PMC10254464; doi:10.34133/research.0163)
Supplement: Supplementary 1 — General procedure of asymmetric catalytic AD and Diels–Alder reactions and analytical data, 1H NMR spectra, 13C NMR spectra, and chiral HPLC of resulting products Fig. S1. 1H NMR Spectra of 1a. Fig. S2. 13C NMR Spectra of 1a. Fig. S3. 1H NMR Spectra of 1b. Fig. S4. 13C NMR Spectra of 1b. Fig. S5. 1H NMR Spectra of 1c. Fig. S6. 13C NMR Spectra of 1c. Fig. S7. 1H NMR Spectra of 1d. Fig. S8. 13C NMR Spectra of 1d. Fig. S9. 1H NMR Spectra of 1e. Fig. S10. 13C NMR Spectra of 1e. Fig. S11. 1H NMR Spectra of 3a. Fig. S12. 13C NMR Spectra of 3a. Fig. S13. 1H NMR Spectra of 3b. Fig. S14. 13C NMR Spectra of 3b. Fig. S15. DLS particle size distribution curves of AD-β obtained in THF/Water mixture (5-5 45% H2O, v/v). Fig. S16. DLS particle size distribution curves of AD-β obtained in THF/Water mixture (1:1 THF: H2O) at times of 30 min, 1 hour, and 2 hours. Fig. S17. Aggregation-induced catalysis on organocatalytic asymmetric Diels-Alder reaction. References [39–41] [file research.0163.f1.pdf]

Supporting Information for

Aggregation-Induced Catalysis (*AIC*): Asymmetric Catalysis via Chiral Aggregates

Yao Tang,[1,†] Yu Wang,[2,†] Qingkai Yuan,[1,†] Sai Zhang,[1] Jia-Yin Wang,[2] Shengzhou Jin,[2] Ting Xu,[2] Junyi Pan,[2] James D. McDonald[1,#] and Guigen Li[1,2,*]

[1]*Department of Chemistry and Biochemistry, Texas Tech University, Lubbock, Texas 79409-1061, USA.*
[2]*School of Chemistry and Chemical Engineering, Nanjing University, Nanjing, 210093, China*

[*]Correspondence should be addressed to Guigen Li; guigen.li@ttu.edu

[†]These authors contributed equally to this work.

[#] Undergraduate participants.

Table of Content

1. General Information S2
2. Synthetic Procedures S3
3. NMR Spectra S7
4. Chiral HPLC chromatographic analysis..S21
5. DLS-based particle size distribution of AD-β S101
6. Diels-Alder Reaction results S102
7. Reference..S103

1. General Information

Unless otherwise stated, all reactions were magnetically stirred and conducted in oven-dried glassware in anhydrous solvents under Ar. Solvents and liquid reagents, as well as solutions of solid or liquid reagents were added directly or via syringes, or micropipette. Cooling baths were prepared in Dewar vessels filled with ice/water (0 °C). Heated oil baths were used for reactions requiring elevated temperatures. Solvents were removed under reduced pressure at 40-65 °C using a rotavapor. All given yields for small molecules are isolated yields of chromatographically and NMR spectroscopically materials.

All commercially available chemicals were used as received without further purification. Solvents as follows: MeOH, EtOH, toluene, EtOAc, DCM, dioxane, hexane, acetone and THF were used without further purification.

The ^{1}H and ^{13}C NMR spectra were recorded in $CDCl_3$ on 400 MHz and 100MHz instruments with TMS as internal standard. For referencing of the ^{1}H NMR spectra, the residual solvent signal (δ = 7.26 for $CDCl_3$) were used. In the case of the ^{13}C NMR spectra, the signal of solvents (δ = 77.06 ± 0.03 for $CDCl_3$) were used. Chemical shifts(δ) were reported in ppm with respect to TMS. Data are represented as follows: chemical shift, multiplicity (s = singlet, d = doublet, t = triplet, m = multiplet), coupling constant (*J*, Hz), and integration. Fluorescence spectra were collected by Agilent Technologies Cary Eclipse Fluorescence Spectrophotometer G9800A and Eclipse ADL program. Optical rotation was determined by the Rudolph polarimeter (Rudolph Research Analytical APIV/2W). Products were identified by HPLC (Dionex Ultimate 3000) using chiral column OD-H and OJ-H (siz3 4.6mm × 250 mmL) from Daicel Chiral Technologies Co., LTD.

2. Synthetic Procedures

2.1 Synthesis of (E)-(3-methoxyprop-1-en-1-yl)benzene

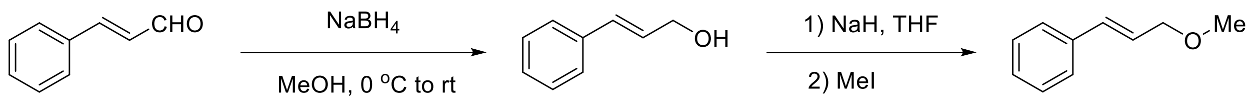

To an oven-dried 100 mL, round bottle flask with a magnetic stirrer, cinnamaldehyde (1.26 mL, 10.0 mmol, 1eq.) was dissolved in 20 mL MeOH at 0 °C, Sodium borohydride (0.46 g, 12.0 mmol, 1.2eq.) was added portion, and the resulting solution was stirred at room temperature for 2 hours. The reaction was quenched by adding 50 mL H_2O, and the solvent was removed by vacuum. The residue was dissolved with 20 mL DCM, and the aqueous layer was washed with 20 mL DCM twice. The combined organic layer was then washed with brine and dried by $MgSO_4$. Evaporated solvent to afford the (E)-3-phenylprop-2-en-1-ol (1.27 g, 95%) as colorless liquid without further purification. ^{1}H NMR (400 MHz, CHLOROFORM-*D*) δ 7.41 – 7.36 (m, 2H), 7.35 – 7.28 (m, 2H), 7.27 – 7.22 (m, 1H), 6.61 (dt, *J* = 15.9, 1.5 Hz, 1H), 6.36 (dt, *J* = 15.9, 5.7 Hz, 1H), 4.31 (dd, *J* = 5.7, 1.5 Hz, 2H).

To an oven-dried 100 mL, round bottle flask with a magnetic stirrer, (E)-3-phenylprop-2-en-1-ol (1.0 g, 7.4 mmol, 1 eq.) was dissolved in 15 mL anhydrous THF under a nitrogen atmosphere 0 °C. Sodium hydride (60% w/ oil suspension, 14.8 mmol, 2eq.) was added portion, and the resulting solution was stirred at 0 °C for 15 minutes. Iodomethane (1.4 mL, 22.2 mmol, 3eq.) was added dropwise, and the reaction was kept stirring for another 15 minutes and then stirred at room temperature overnight. The reaction was quenched by 5 mL NH_4Cl at 0 °C, and the solvent was removed by vacuum. The residue was dissolved with 20 mL ethyl acetate, and the aqueous layer was washed with 20 mL ethyl acetate twice. The combined organic layer was then washed with brine and dried by $MgSO_4$. The organic phase was concentrated under reduced pressure, and the product can be obtained by flash chromatography (Hexane/Ethyl acetate = 10:1) as a colorless liquid (0.88 g, 80%). ^{1}H NMR (400 MHz, CHLOROFORM-*D*) δ 7.45 – 7.41 (m, 2H), 7.38 – 7.32 (m, 2H), 7.30 – 7.25 (m, 1H), 6.65 (dt, *J* = 15.9, 1.6 Hz, 1H), 6.37 – 6.28 (m, 1H), 4.12 (dd, *J* = 6.0, 1.5 Hz, 2H), 3.42 (s, 3H).

2.2 General procedures for asymmetric dihydroxylation (AD) reaction[1]

To a 10 mL reaction tube equipped with a magnetic stirrer was charged with various ratio solvent mixtures (THF/H2O, total volume 2 mL) and 0.28 g AD-mix-α or AD-mix-β. The solution was stirred at room temperature for 10 minutes, and 0.2 mmol styrene was added at once. The resulting mixture was stirred vigorously for 24 hours until the TLC indicated styrene was completely consumed. Anhydrous sodium sulfite was added to quench the reaction and allowed to stir for another hour. The aqueous layers were washed with 5 mL ethyl acetate twice, and the combined organic layer was concentrated to determine the yield and subsequently purified by flash column chromatography to obtain the pure product for HPLC analysis.

(R)-1-phenylethane-1,2-diol

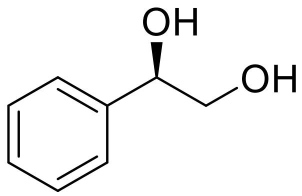

1a

^{1}H NMR (400 MHz, CHLOROFORM-*D*) δ 7.39 – 7.25 (m, 5H), 4.79 (dd, *J* = 8.2, 3.5 Hz, 1H), 3.78 – 3.59 (m, 2H), 2.70 (s, 2H). **^{13}C NMR** (101 MHz, CHLOROFORM-*D*) δ 140.56, 128.65, 128.12, 126.17, 74.80, 68.17.

(R)-1-(o-tolyl)ethane-1,2-diol

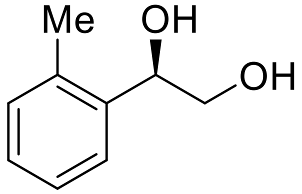

1b

^{1}H NMR (400 MHz, CHLOROFORM-*D*) δ 7.45 (dd, *J* = 7.2, 1.9 Hz, 1H), 7.24 – 7.10 (m, 3H), 5.03 (dd, *J* = 8.6, 3.2 Hz, 1H), 3.72 – 3.54 (m, 2H), 2.96 (s, 2H), 2.31 (s, 3H). **^{13}C NMR** (101 MHz, CHLOROFORM-*D*) δ 138.52, 134.85, 130.54, 127.85, 126.41, 125.74, 71.54, 67.01, 19.12.

(R)-1-(m-tolyl)ethane-1,2-diol

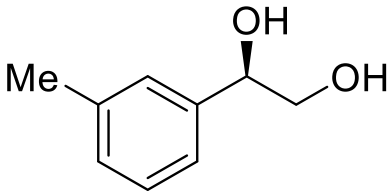

1c

^{1}H NMR (400 MHz, CHLOROFORM-*D*) δ 7.20 (t, *J* = 7.5 Hz, 1H), 7.12 – 7.06 (m, 3H), 4.71 (dd, *J* = 8.6, 3.3 Hz, 1H), 4.32 (s, 2H), 3.68 – 3.53 (m, 2H), 2.31 (s, 3H). **^{13}C NMR** (101 MHz, CHLOROFORM-*D*) δ 140.64, 138.15, 128.67, 128.47, 126.91, 123.28, 74.88, 68.11, 21.50.

(R)-1-(p-tolyl)ethane-1,2-diol

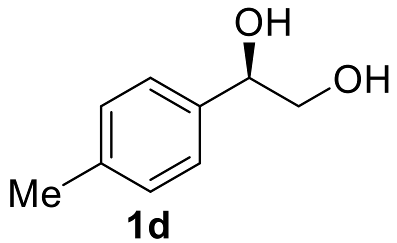

1d

^{1}**H NMR** (400 MHz, CHLOROFORM-*D*) δ 7.22 (d, *J* = 8.1 Hz, 2H), 7.14 (d, *J* = 7.9 Hz, 2H), 4.74 (dd, *J* = 8.3, 3.5 Hz, 1H), 3.72 – 3.57 (m, 2H), 2.92 (s, 2H), 2.33 (s, 3H). ^{13}**C NMR** (101 MHz, CHLOROFORM-*D*) δ 137.81, 137.59, 129.30, 126.12, 74.67, 68.16.

(R)-1-(4-methoxyphenyl)ethane-1,2-diol

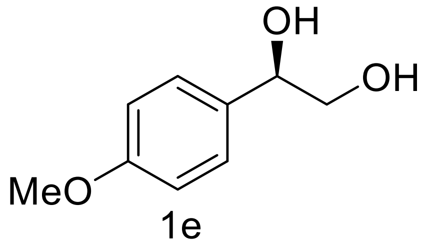

1e

^{1}**H NMR** (400 MHz, CHLOROFORM-*D*) δ 7.25 (d, *J* = 8.6 Hz, 2H), 6.87 (d, *J* = 8.7 Hz, 2H), 4.73 (dd, *J* = 8.2, 3.6 Hz, 1H), 3.78 (s, 3H), 3.70 – 3.58 (m, 2H), 2.76 (s, 2H). ^{13}**C NMR** (101 MHz, CHLOROFORM-*D*) δ 159.46, 132.71, 127.45, 114.03, 74.39, 68.14.

(R)-1-(4-chlorophenyl)ethane-1,2-diol

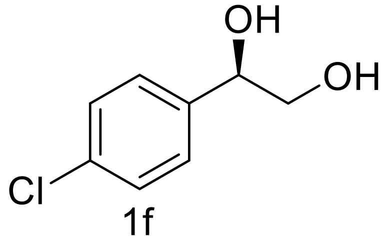

1f

^{1}**H NMR** (400 MHz, CHLOROFORM-*D*) δ 7.34 – 7.29 (m, 2H), 7.29 – 7.24 (m, 2H), 4.76 (dd, *J* = 8.2, 3.4 Hz, 1H), 3.75 – 3.54 (m, 2H), 2.70 (s, 2H). ^{13}**C NMR** (101 MHz, CHLOROFORM-*D*) δ 138.96, 133.84, 128.80, 127.54, 74.09, 68.00.

(R)-1-(2,6-dimethylphenyl)ethane-1,2-diol

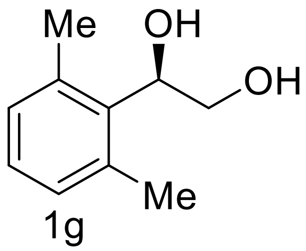

1g

^{1}H NMR (400 MHz, CHLOROFORM-*D*) δ 7.06 (dd, *J* = 8.4, 6.5 Hz, 1H), 6.98 (d, *J* = 7.5 Hz, 2H), 5.27 (dd, *J* = 9.9, 3.8 Hz, 1H), 3.96 (ddd, *J* = 10.7, 9.8, 0.9 Hz, 1H), 3.61 (ddd, *J* = 11.6, 3.7, 1.6 Hz, 1H), 2.64 (s, 2H), 2.43 (s, 6H). **^{13}C NMR** (101 MHzU8, CHLOROFORM-*D*) δ 136.81, 135.52, 129.54, 127.73, 72.91, 64.58, 20.99.

(1R,2R)-1-phenylpropane-1,2-diol

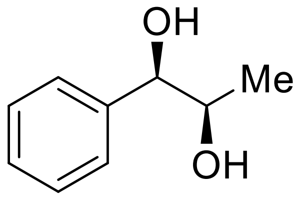

3a

^{1}H NMR (400 MHz, CHLOROFORM-*D*) δ 7.31 (qd, *J* = 7.1, 3.3 Hz, 5H), 4.33 (d, *J* = 7.4 Hz, 1H), 3.83 (dd, *J* = 7.3, 6.2 Hz, 1H), 2.93 (s, 2H), 1.02 (d, *J* = 6.3 Hz, 3H). **^{13}C NMR** (101 MHz, CHLOROFORM-*D*) δ 141.12, 128.62, 128.26, 126.95, 79.61, 72.33, 18.85.

(1R,2R)-3-methoxy-1-phenylpropane-1,2-diol

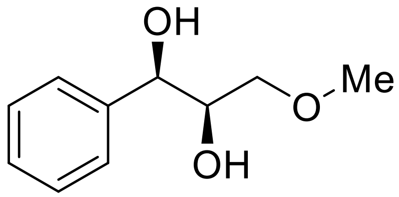

3b

^{1}H NMR (400 MHz, CHLOROFORM-*D*) δ 7.32 (d, *J* = 4.3 Hz, 4H), 7.30 – 7.24 (m, 1H), 4.61 (d, *J* = 6.8 Hz, 1H), 3.77 (ddd, *J* = 6.8, 5.7, 3.2 Hz, 1H), 3.53 (s, 2H), 3.31 – 3.27 (m, 1H), 3.26 (s, 3H), 3.21 (dd, *J* = 9.9, 5.8 Hz, 1H). **^{13}C NMR** (101 MHz, CHLOROFORM-*D*) δ 140.82, 128.53, 128.05, 126.84, 74.83, 73.53, 59.20.

3. NMR Spectra

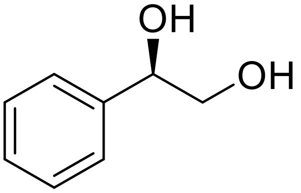

1a

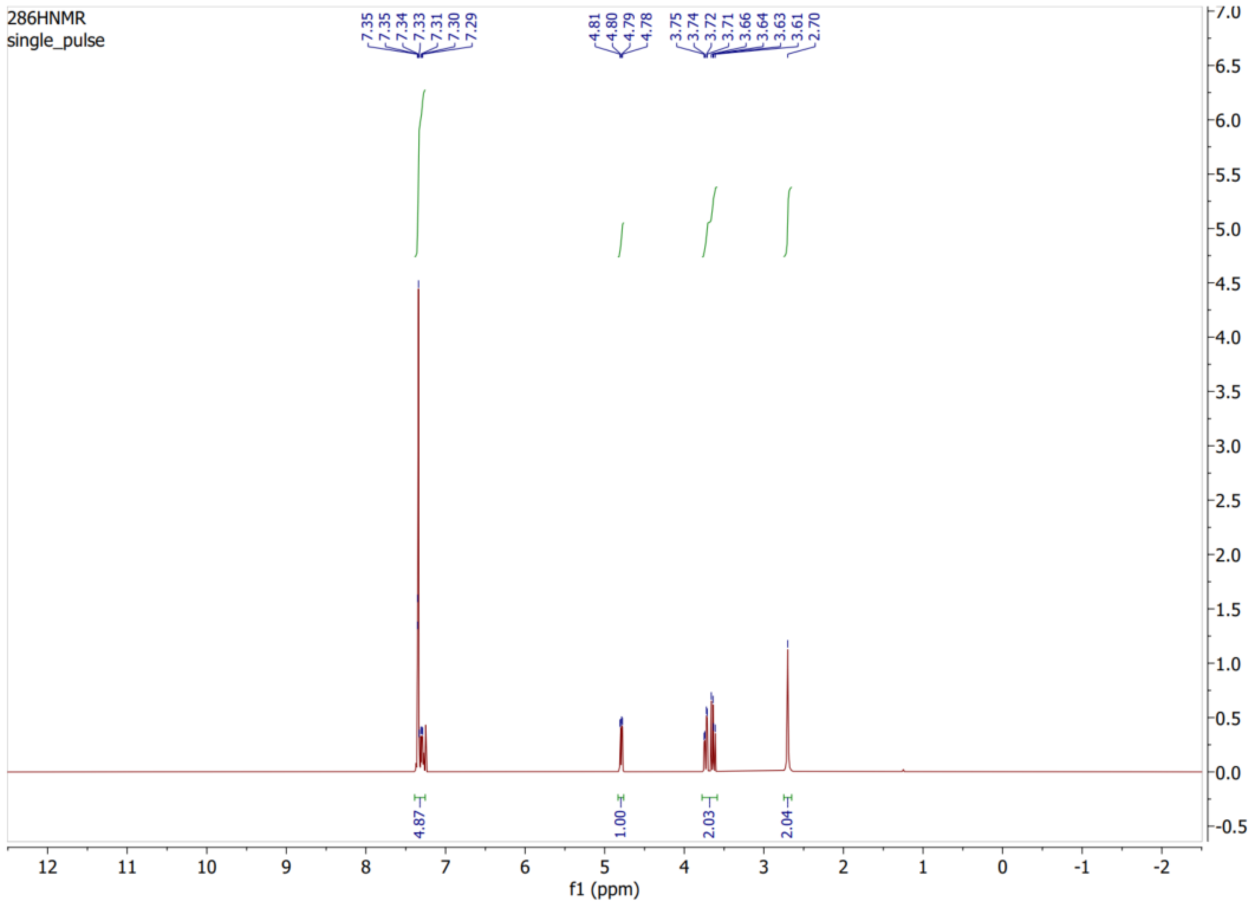

Figure S1. ^{1}H NMR Spectra of 1a

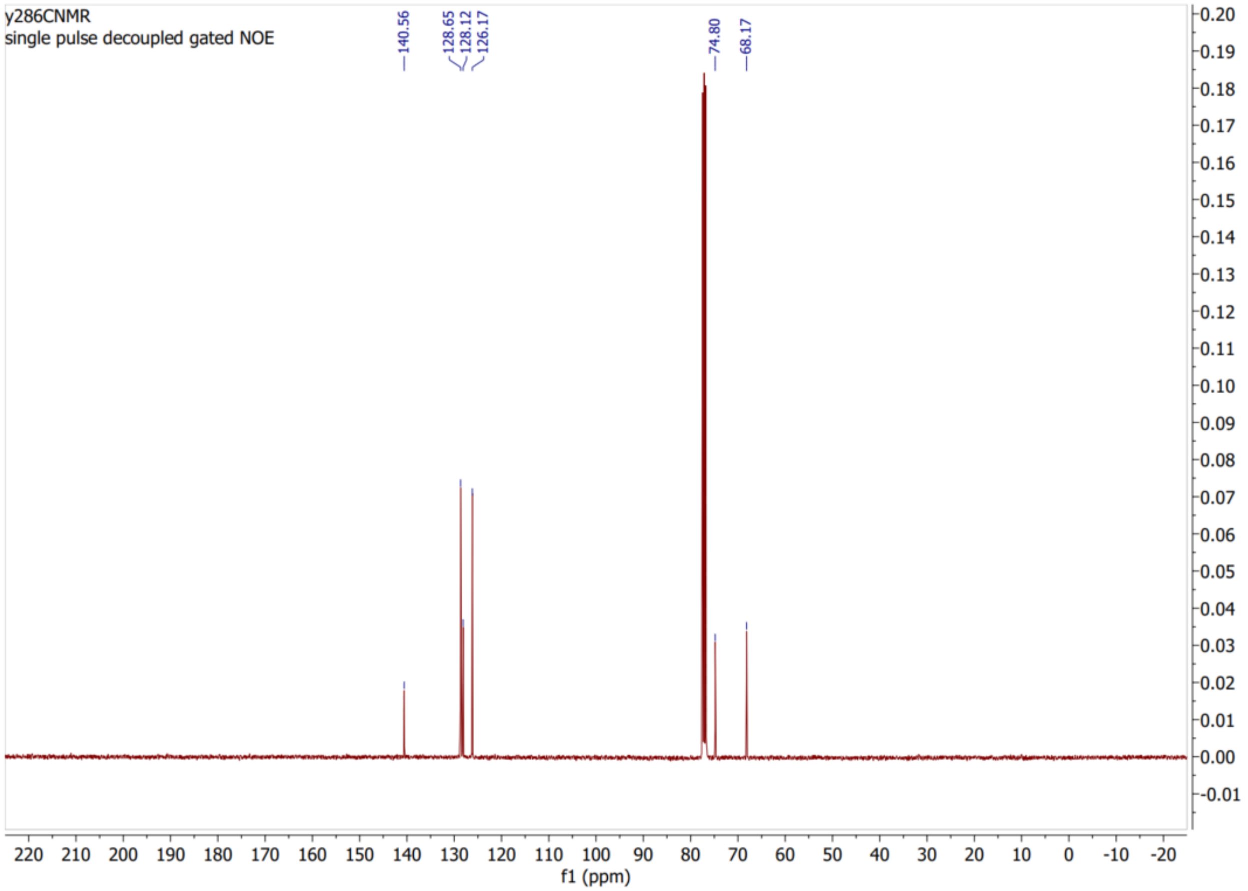

Figure S2. ^{13}**C NMR Spectra of 1a**

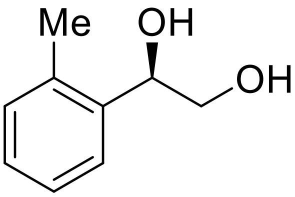

1b

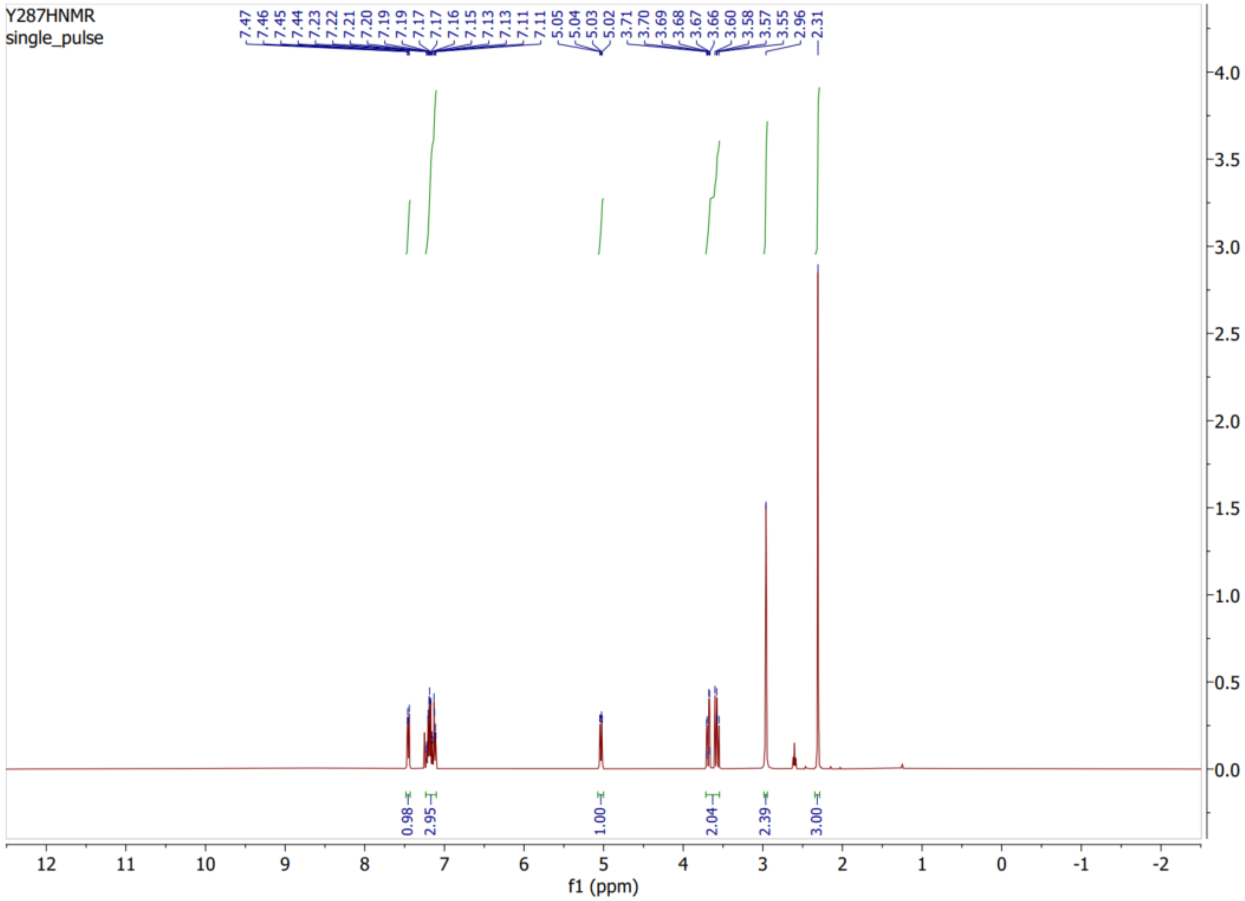

Figure S3. ^{1}**H NMR Spectra of 1b**

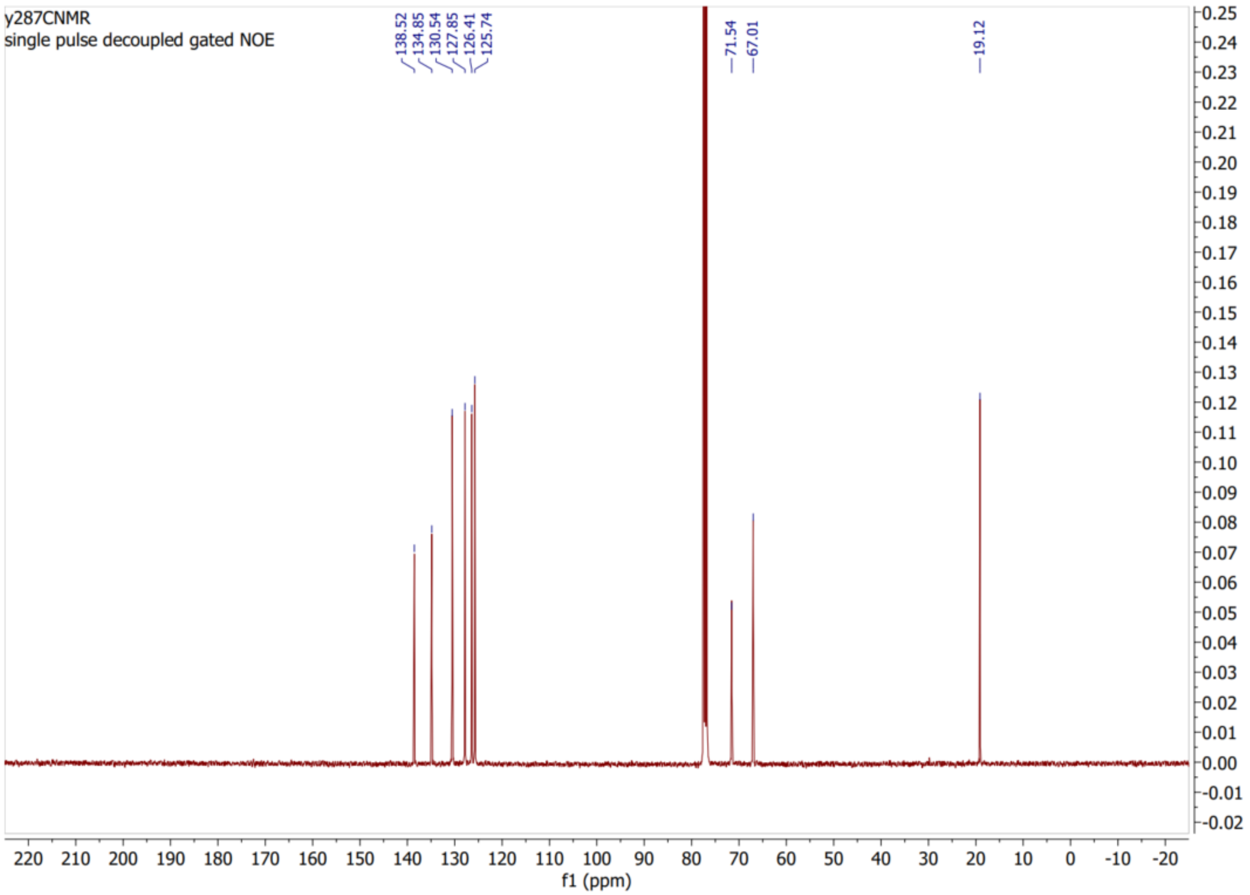

Figure S4. ^{13}**C NMR Spectra of 1b**

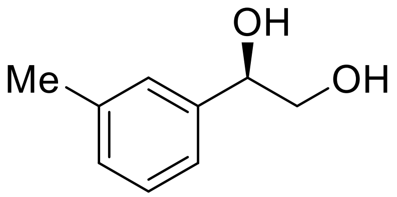

1c

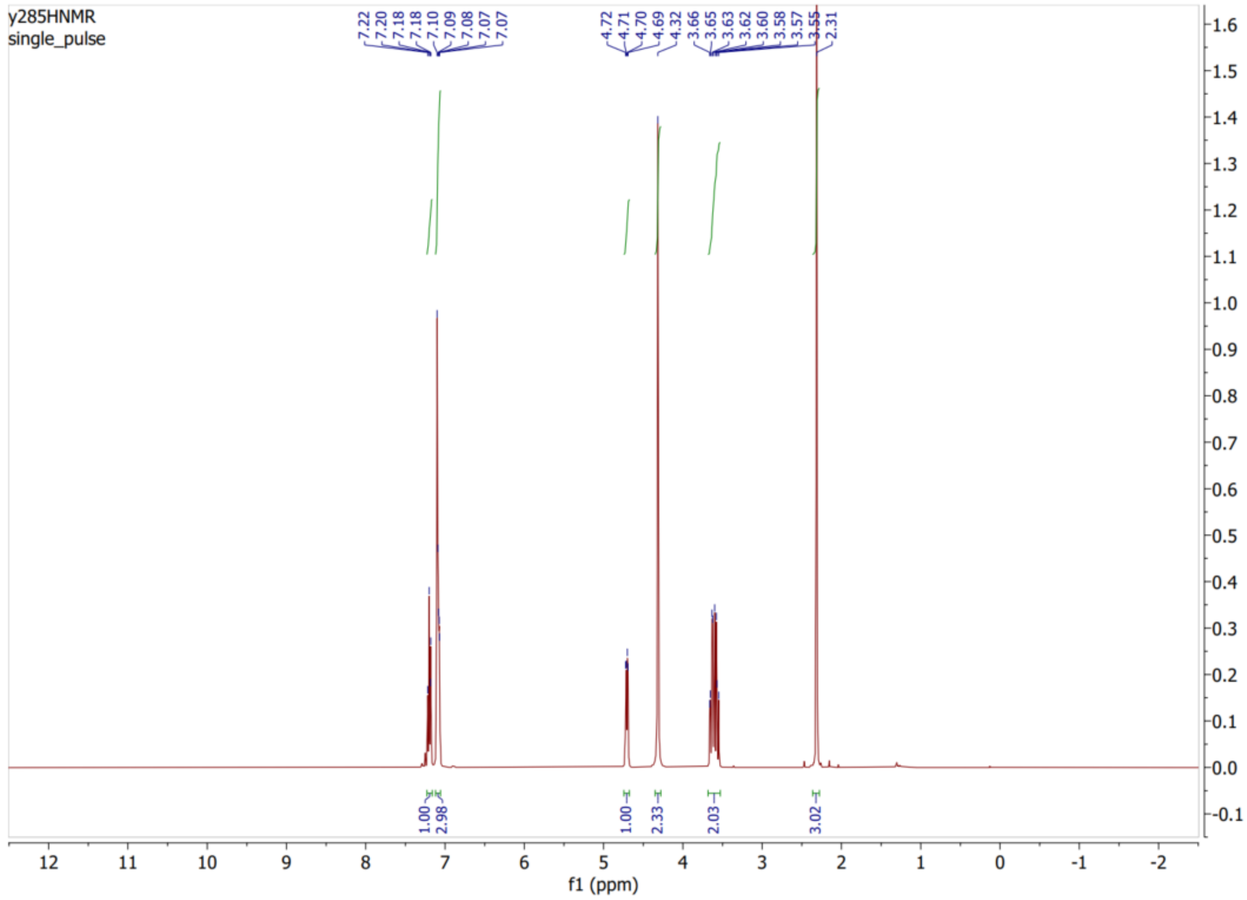

Figure S5. [1]**H NMR Spectra of 1c**

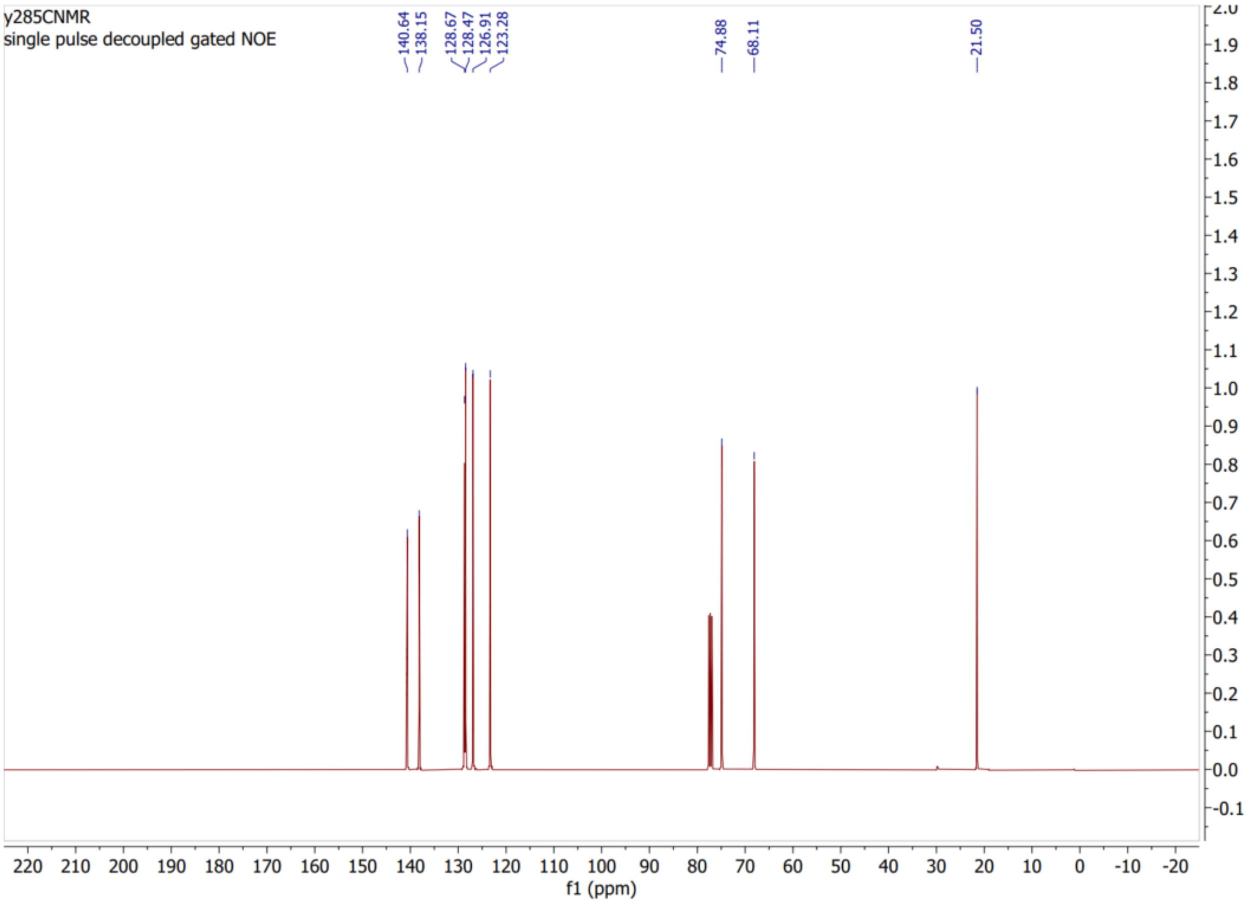

Figure S6. ^{13}**C NMR Spectra of 1c**

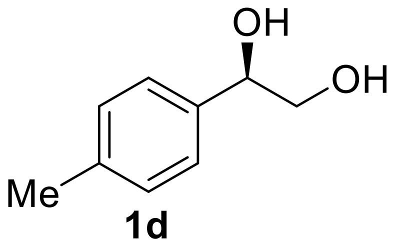

1d

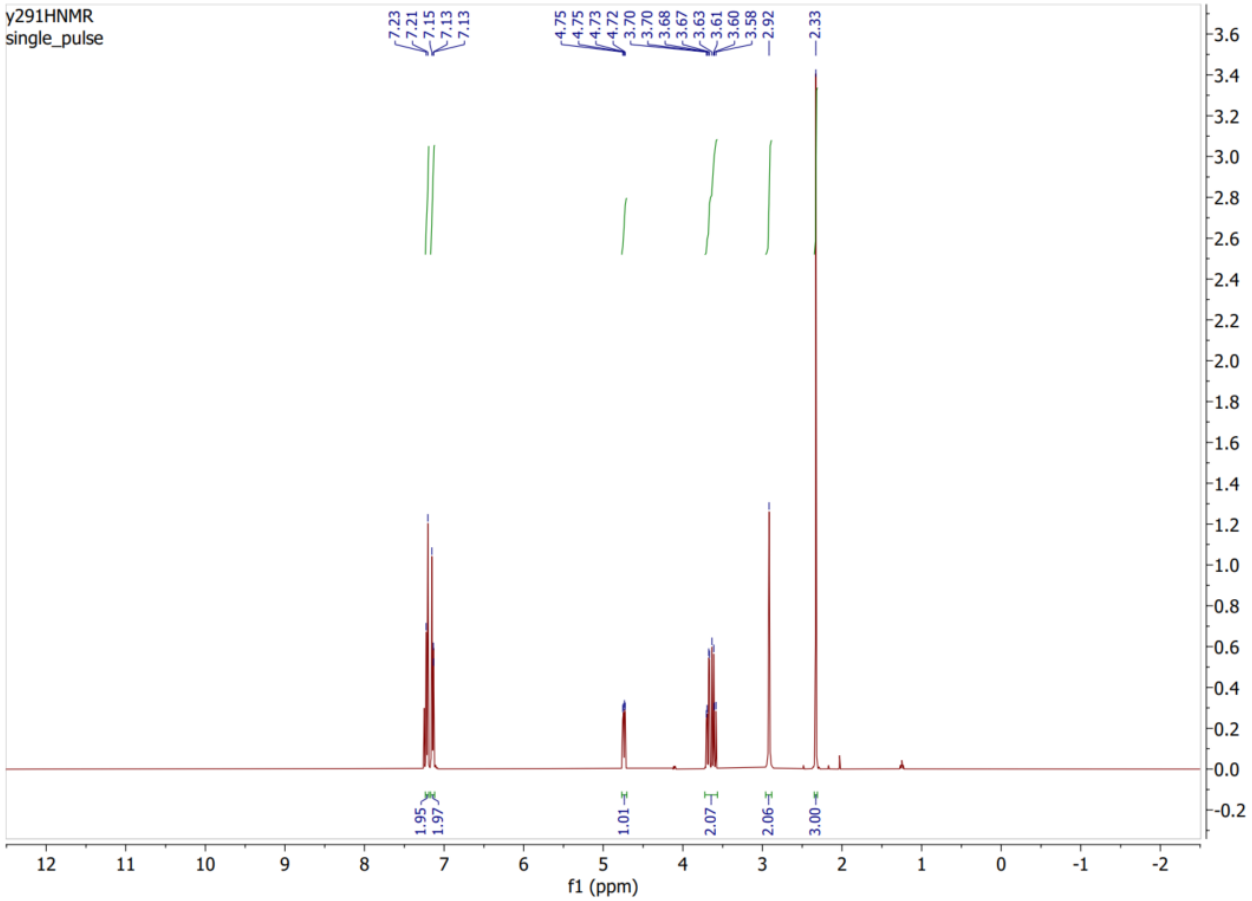

Figure S7. ^{1}H NMR Spectra of 1d

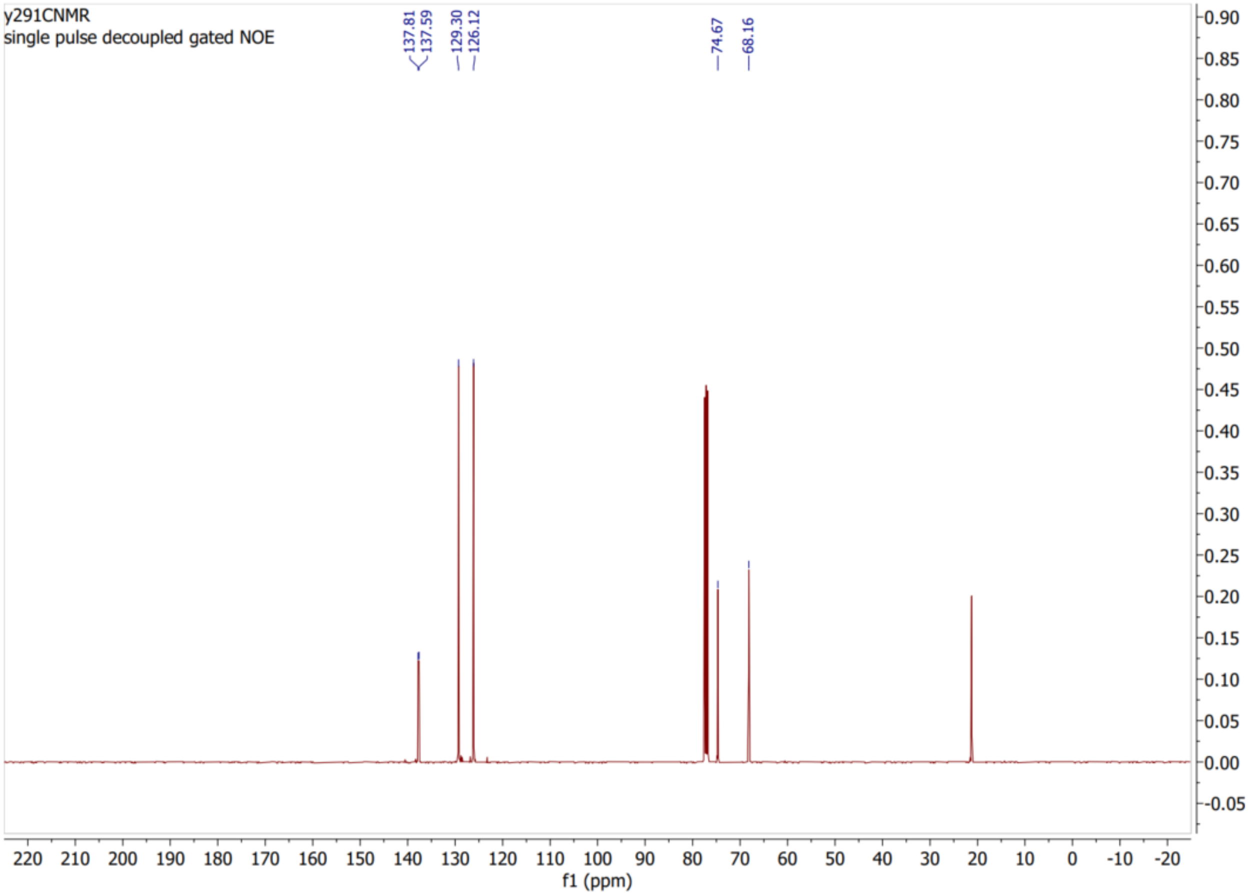

Figure S8. ^{13}C NMR Spectra of 1d

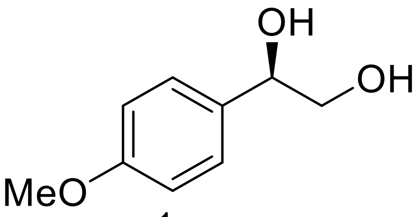

1e

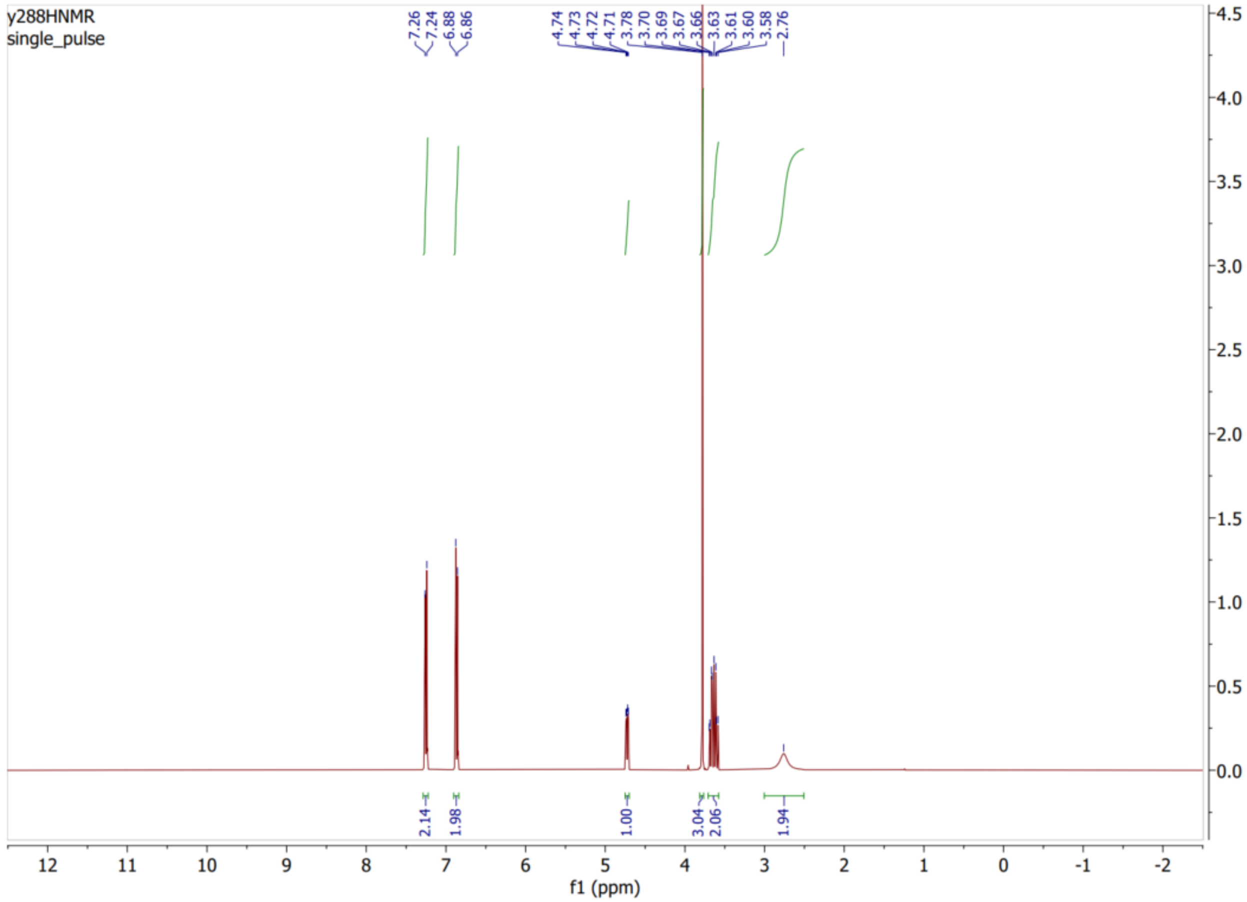

Figure S9. ^{1}H NMR Spectra of 1e

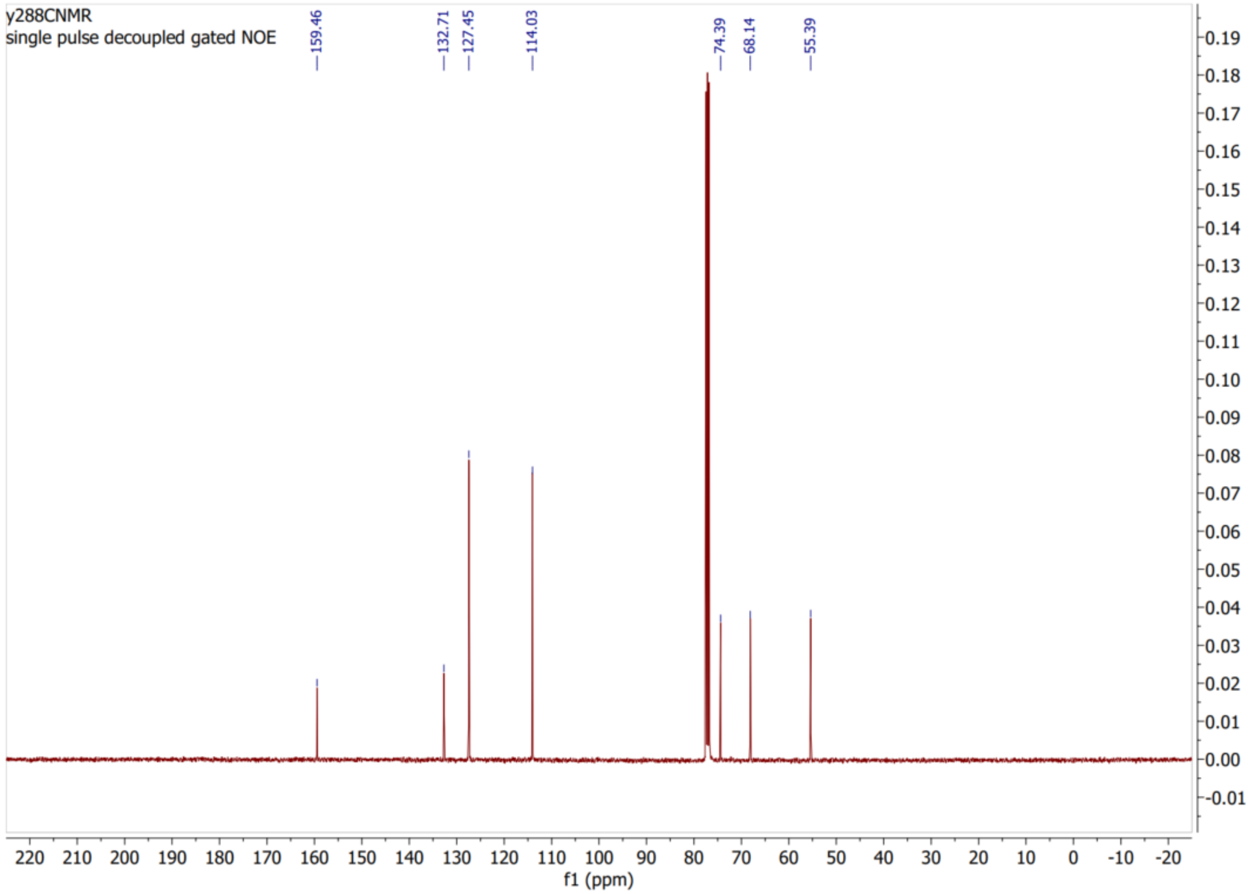

Figure S10. ^{13}**C NMR Spectra of 1e**

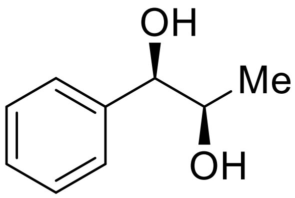

3a

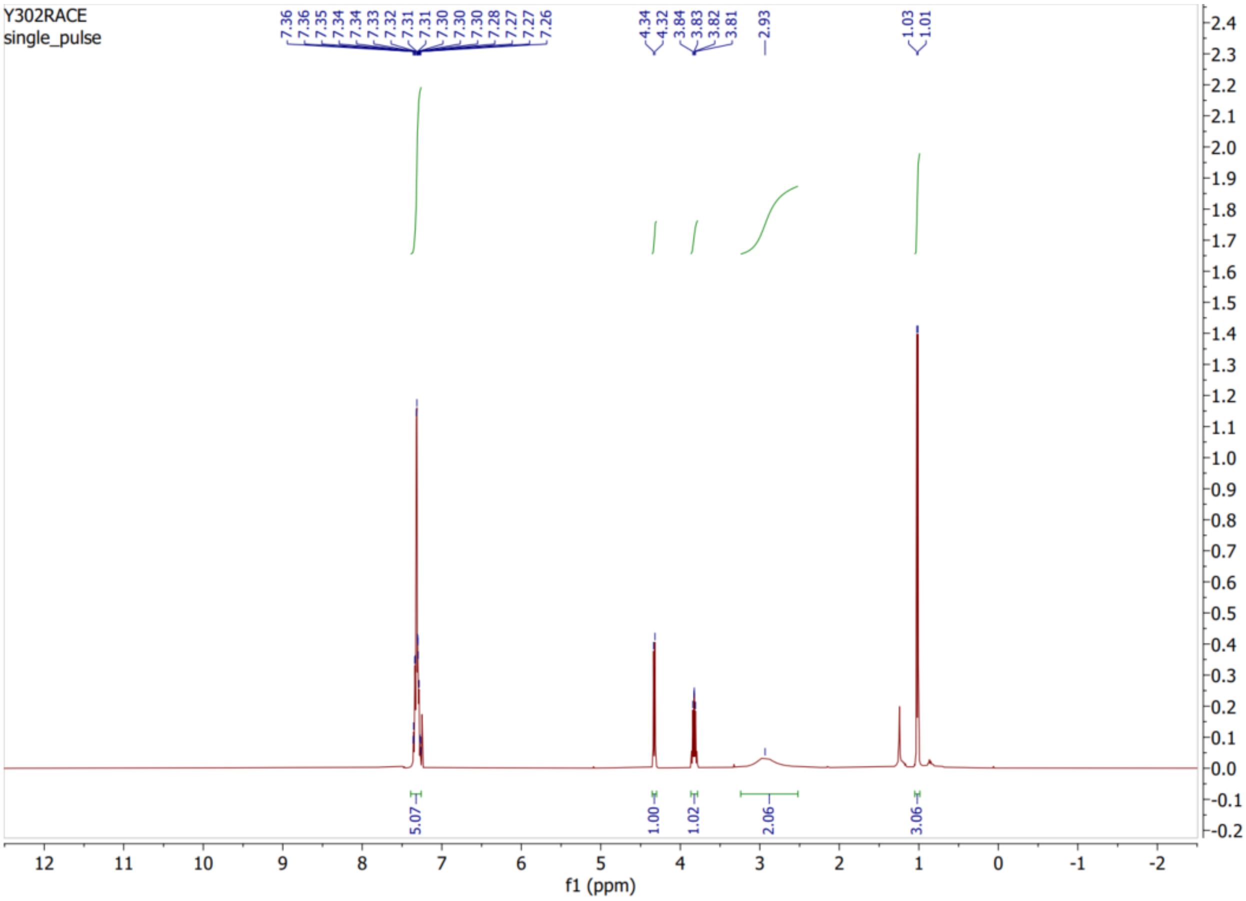

Figure S11. **^{1}H NMR Spectra of 3a**

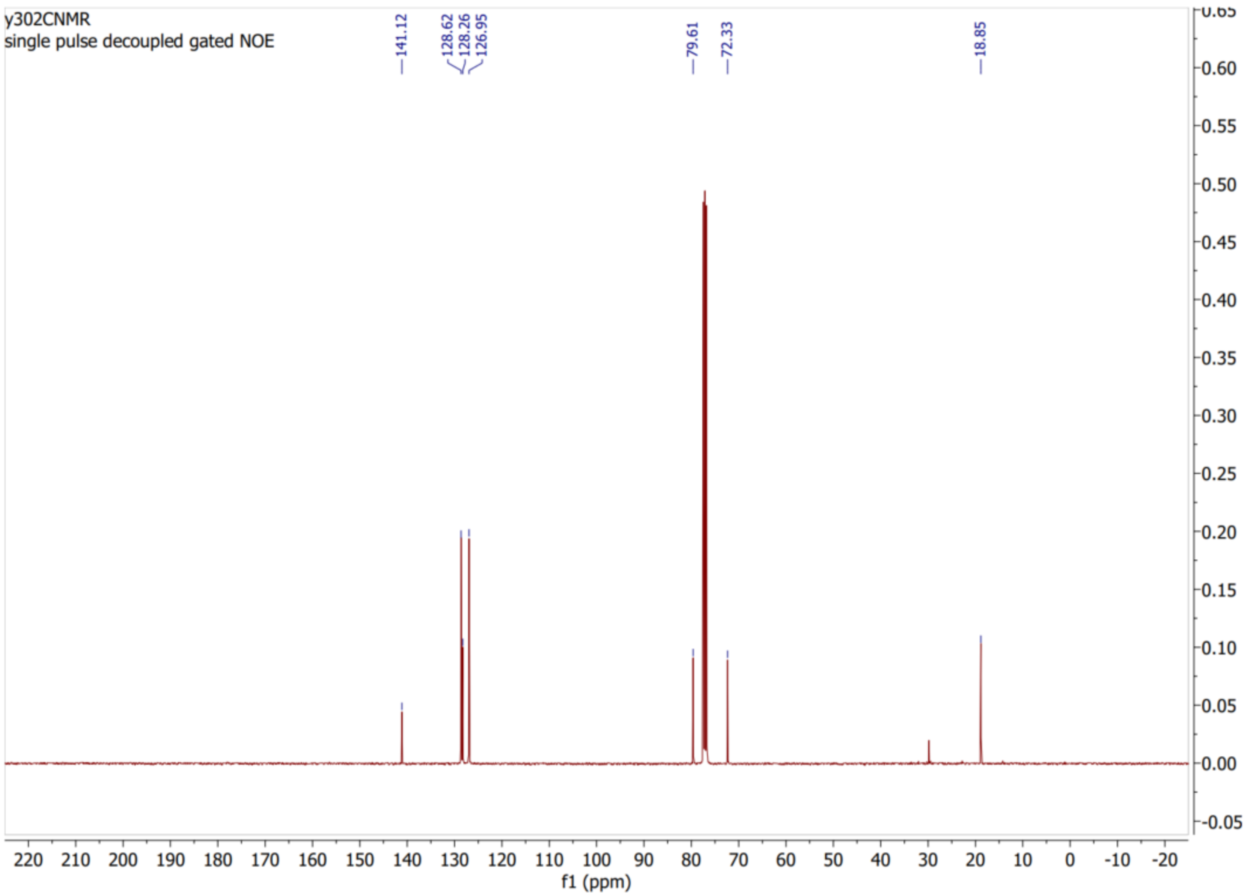

Figure S12. ^{13}**C NMR Spectra of 3a**

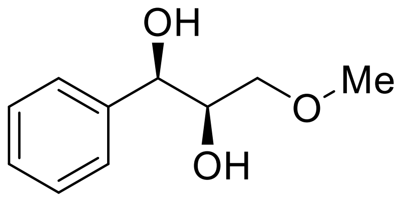

3b

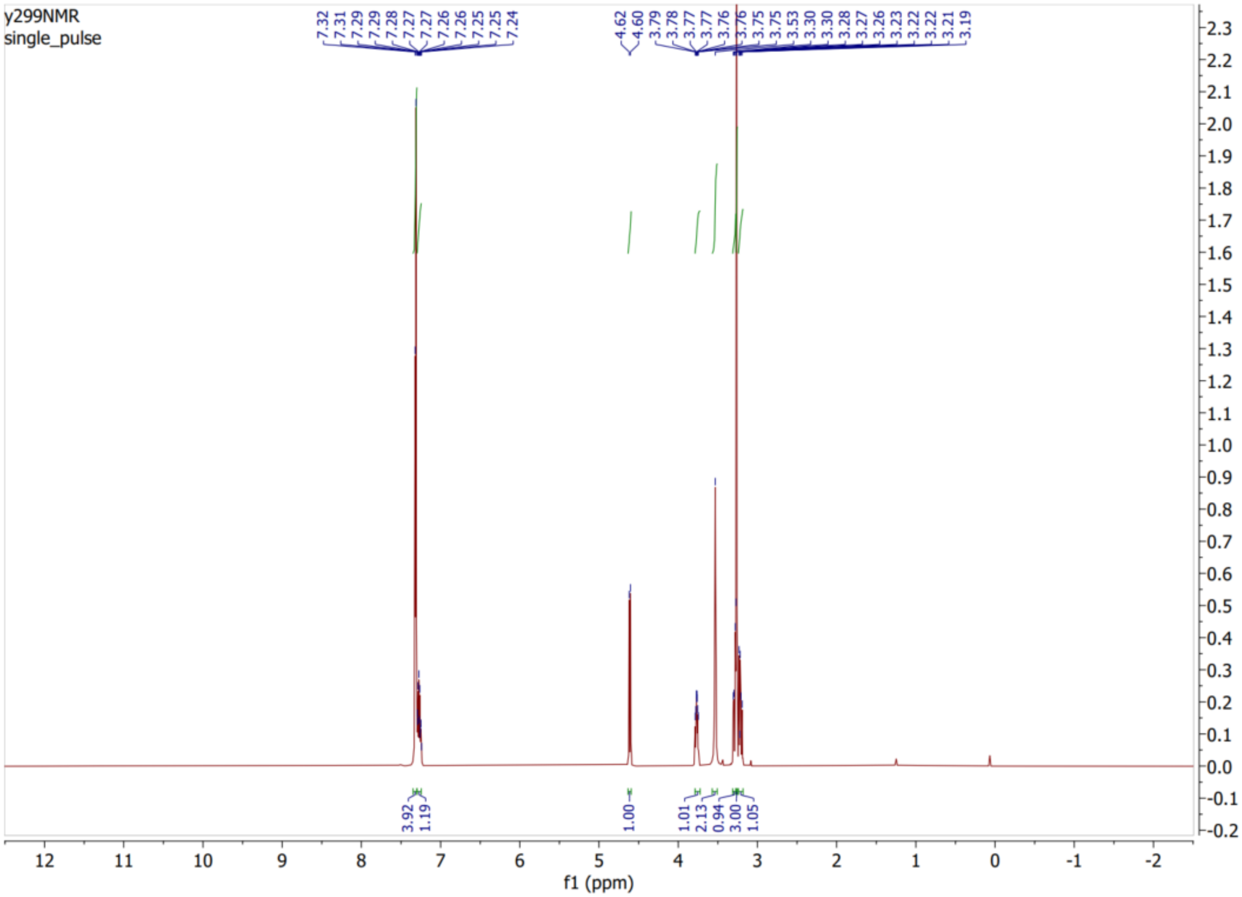

Figure S13. [1]**H NMR Spectra of 3b**

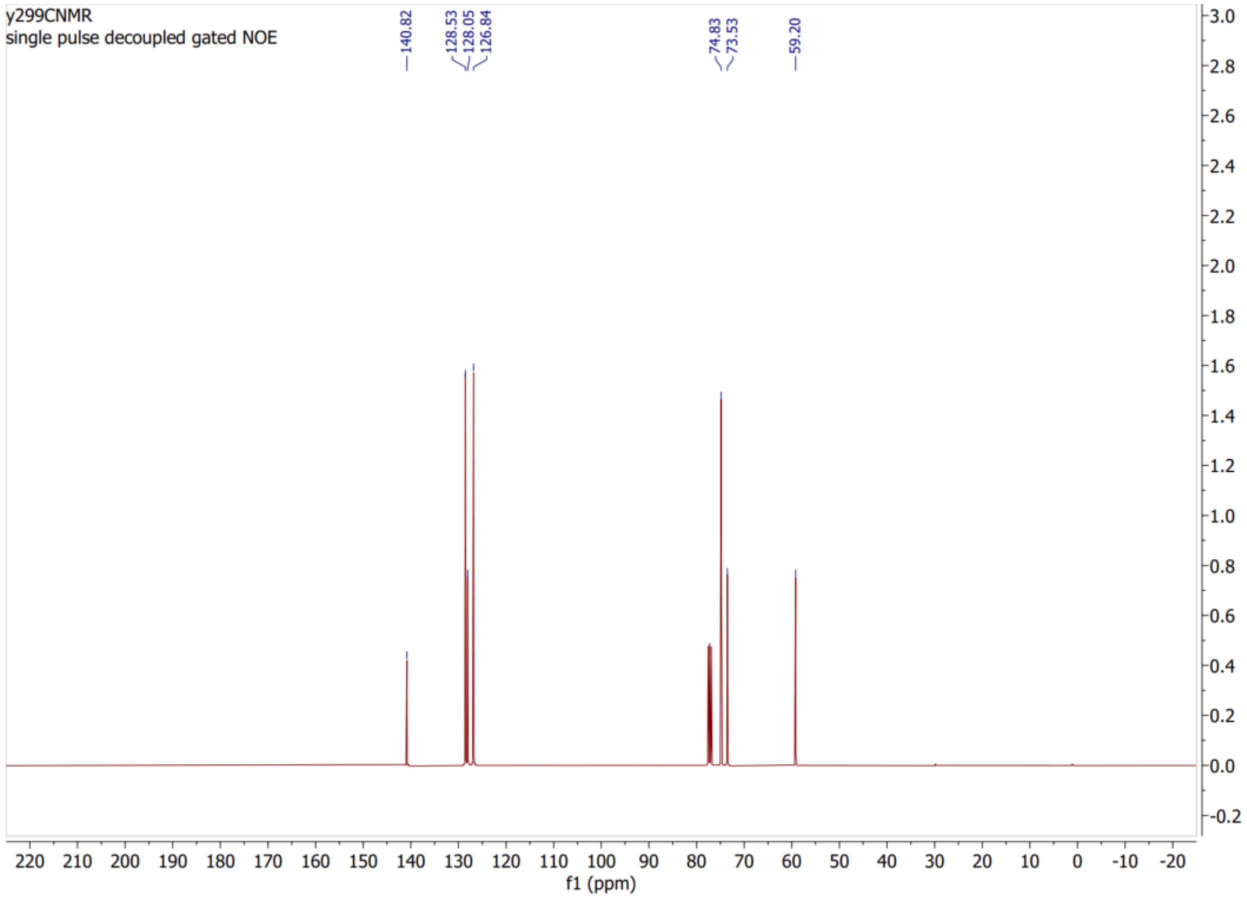

Figure S14. ^{13}C NMR Spectra of 3b

4. Chiral HPLC chromatographic analysis[2]

(R)-1-phenylethane-1,2-diol

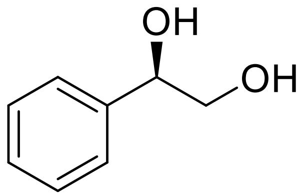

1a

- Column: Chiralpak OD-H.
- Condition: 10% *i*PrOH/Hexane at 0.5 mL/min, enantiomeric excess determined at 210 nm; 15.6 min (R), 16.8 min (S).

THF:H_2O =4.5:1; %ee = 58%

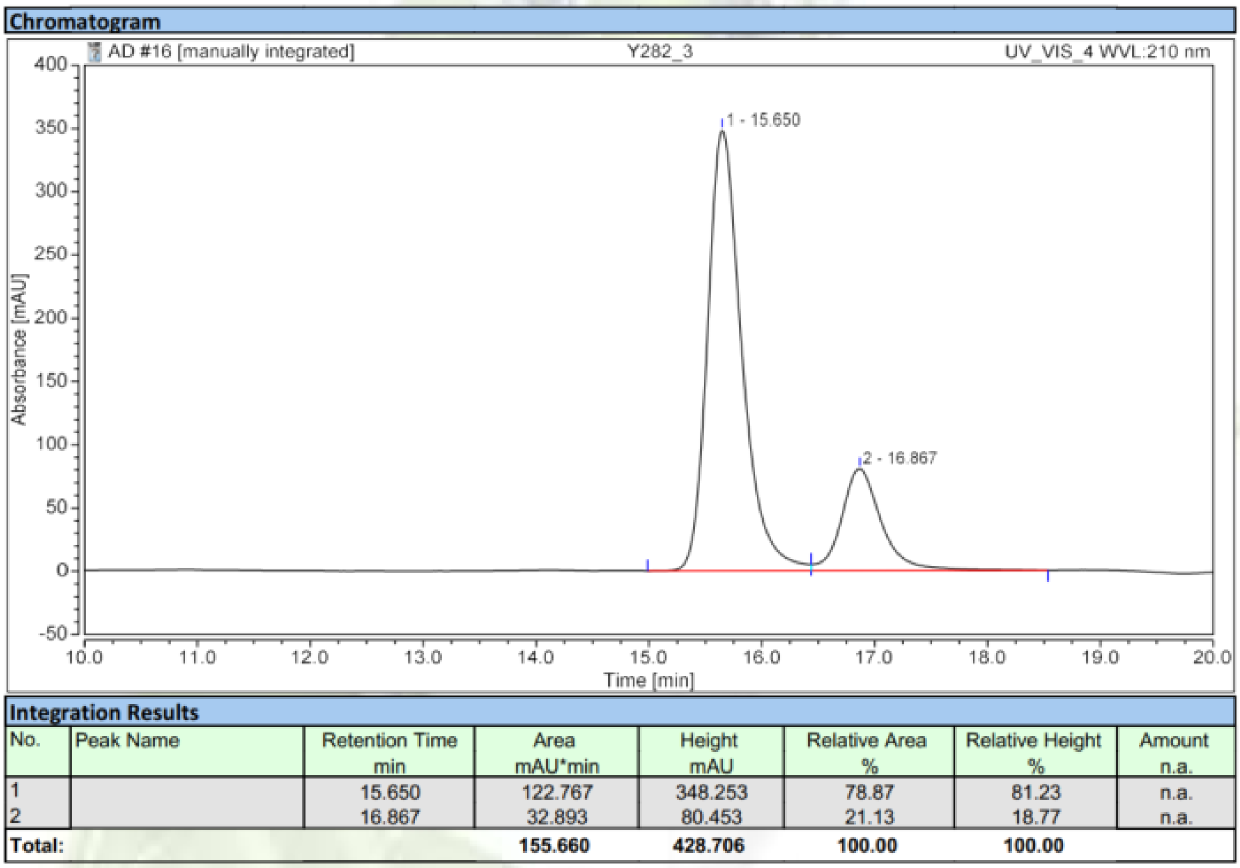

Integration Results

No.	Peak Name	Retention Time min	Area mAU*min	Height mAU	Relative Area %	Relative Height %	Amount n.a.
1		15.650	122.767	348.253	78.87	81.23	n.a.
2		16.867	32.893	80.453	21.13	18.77	n.a.
**Total:**			**155.660**	**428.706**	**100.00**	**100.00**	

THF:H_2O =3:1; %ee = 56%

Chromatogram

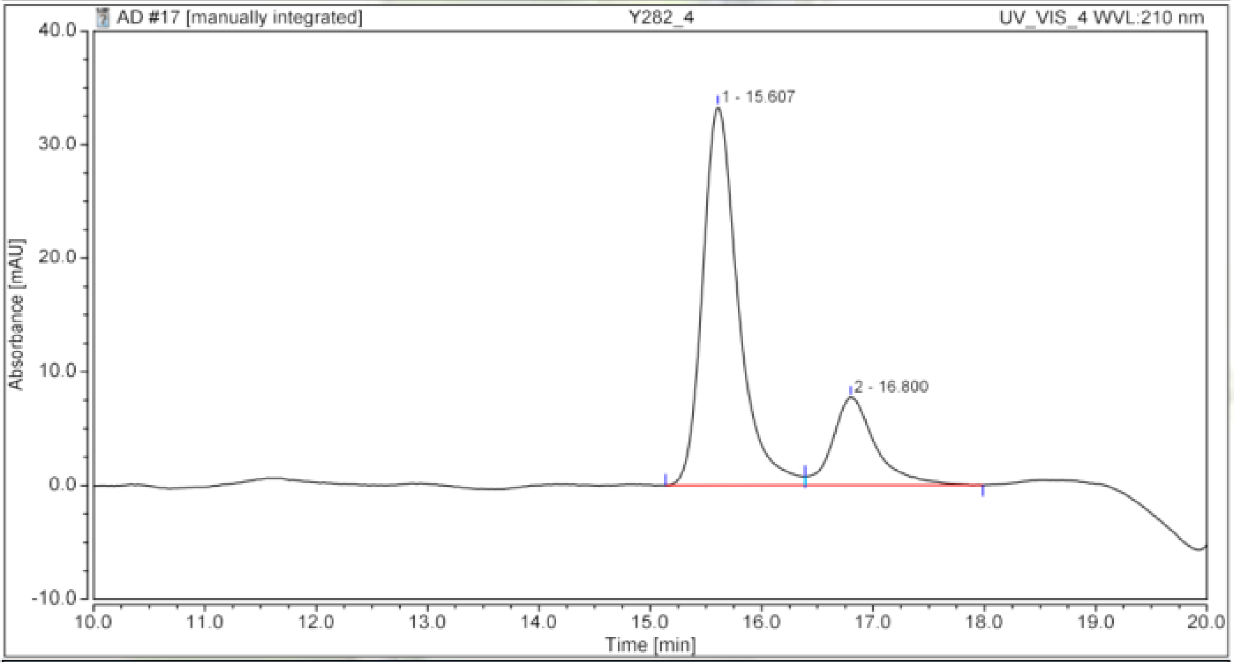

Integration Results

No.	Peak Name	Retention Time min	Area mAU*min	Height mAU	Relative Area %	Relative Height %	Amount n.a.
1		15.607	12.308	33.293	78.21	81.17	n.a.
2		16.800	3.429	7.722	21.79	18.83	n.a.
**Total:**			**15.737**	**41.015**	**100.00**	**100.00**	

THF:H_2O =1.5:1; %ee = 56%

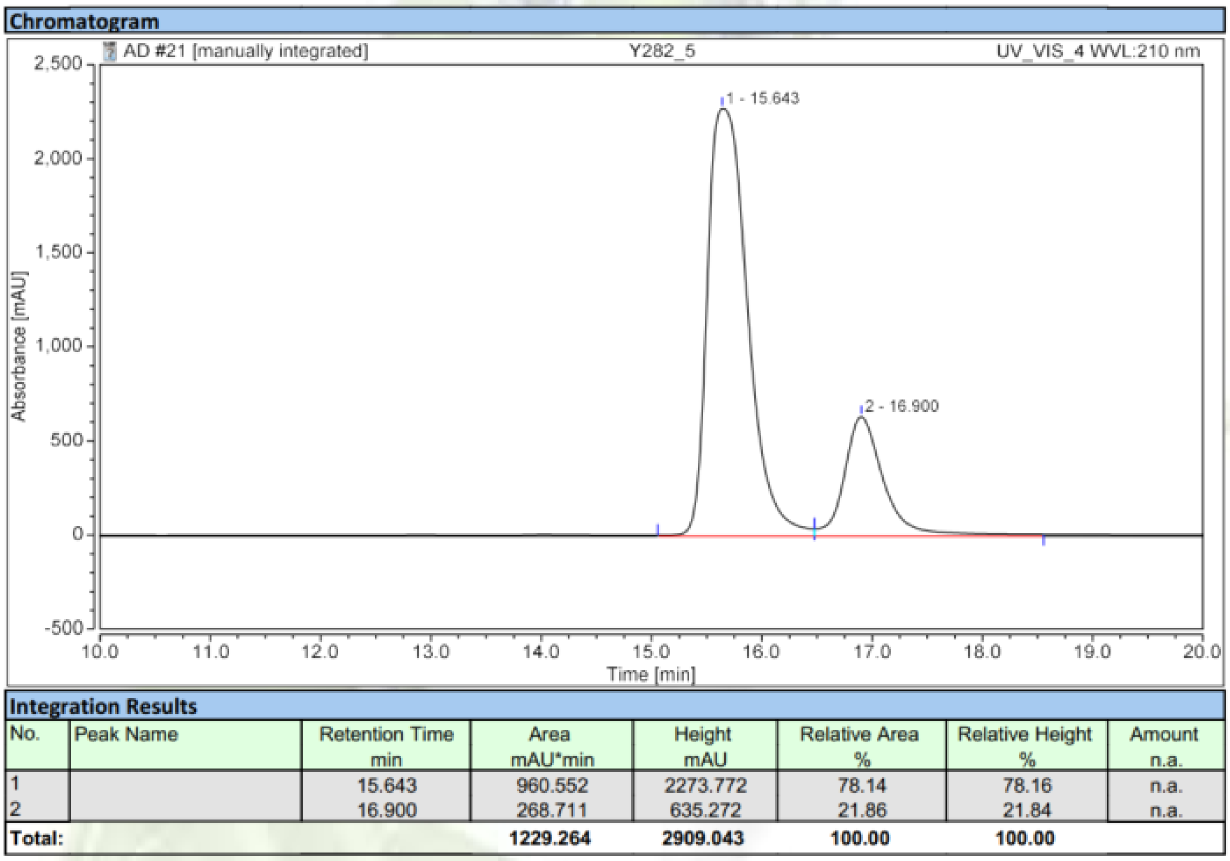

Integration Results

No.	Peak Name	Retention Time min	Area mAU*min	Height mAU	Relative Area %	Relative Height %	Amount n.a.
1		15.643	960.552	2273.772	78.14	78.16	n.a.
2		16.900	268.711	635.272	21.86	21.84	n.a.
**Total:**			**1229.264**	**2909.043**	**100.00**	**100.00**	

THF:H_2O =1:1; %ee = 72%

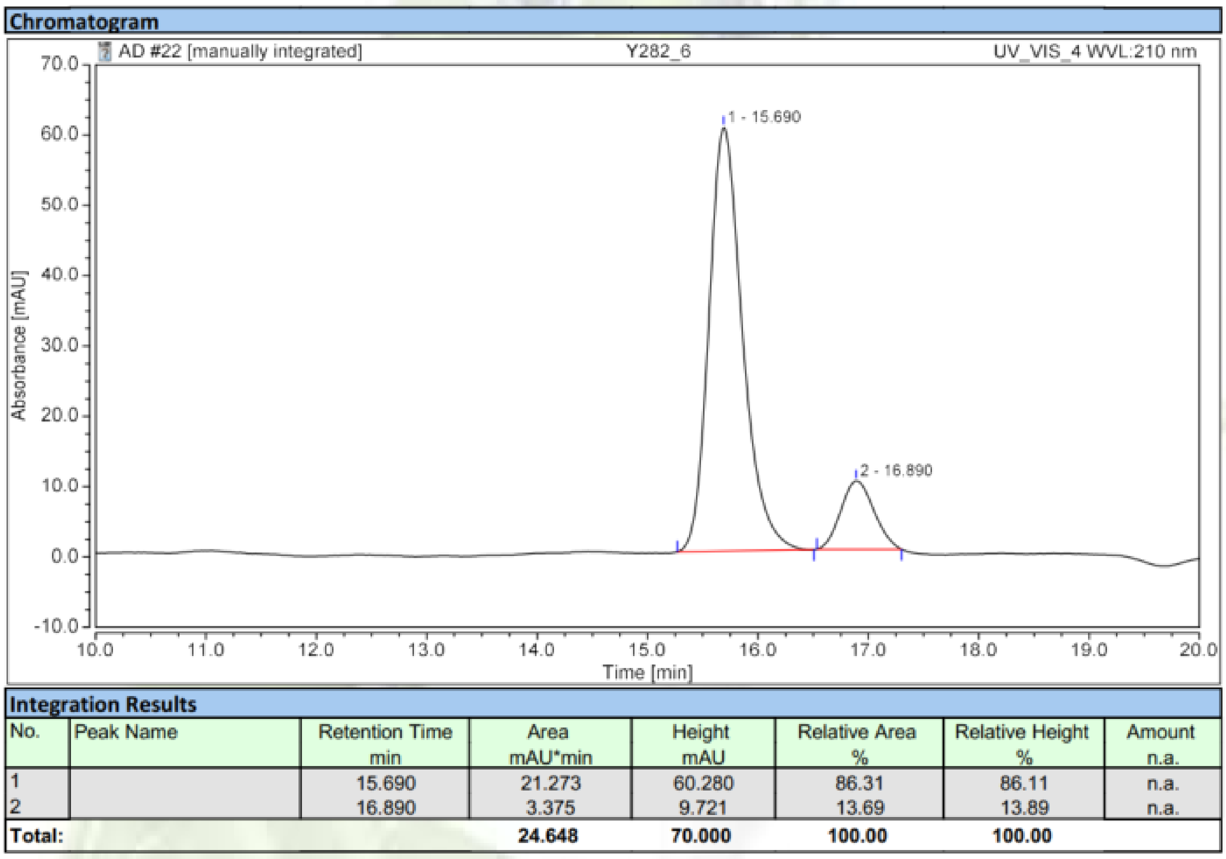

Integration Results

No.	Peak Name	Retention Time min	Area mAU*min	Height mAU	Relative Area %	Relative Height %	Amount n.a.
1		15.690	21.273	60.280	86.31	86.11	n.a.
2		16.890	3.375	9.721	13.69	13.89	n.a.
**Total:**			**24.648**	**70.000**	**100.00**	**100.00**	

THF:H_2O =1:1.5; %ee = 78%

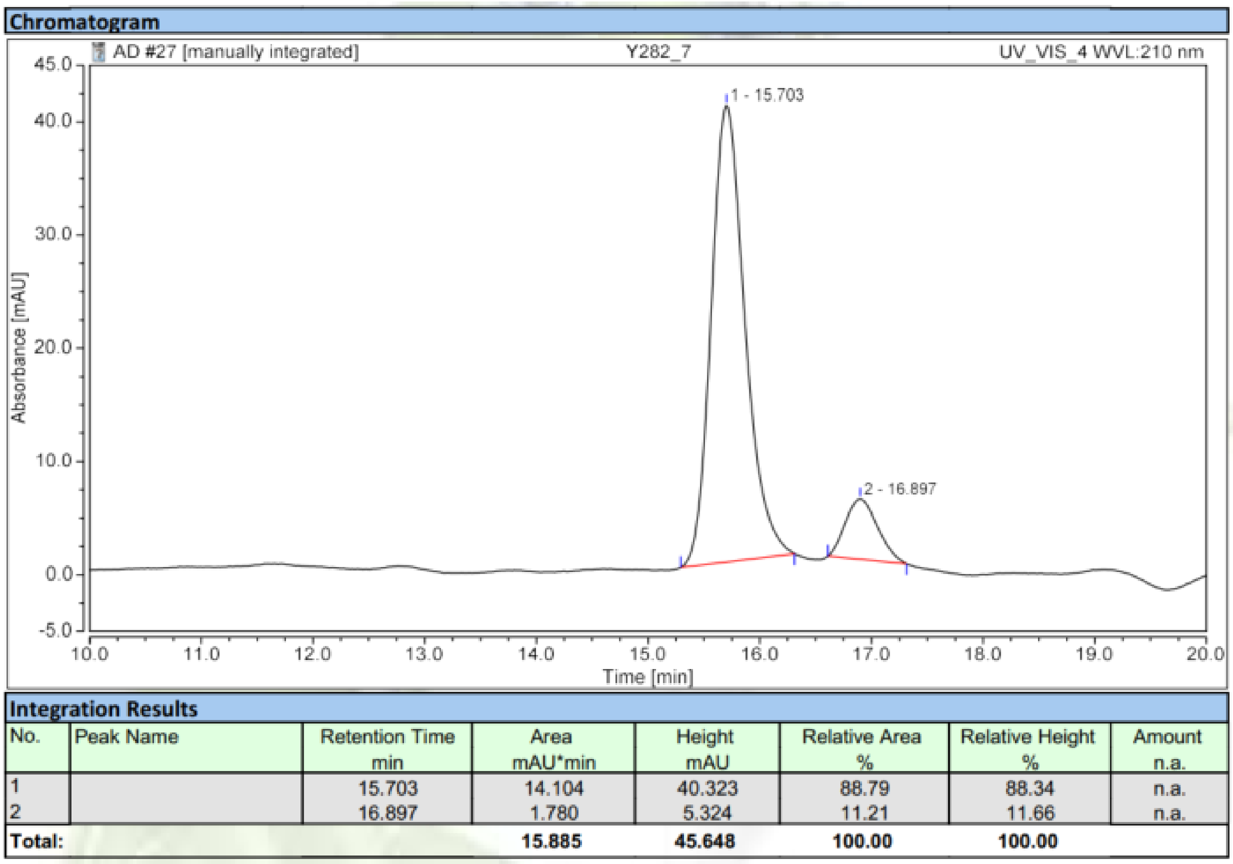

Integration Results

No.	Peak Name	Retention Time min	Area mAU*min	Height mAU	Relative Area %	Relative Height %	Amount n.a.
1		15.703	14.104	40.323	88.79	88.34	n.a.
2		16.897	1.780	5.324	11.21	11.66	n.a.
**Total:**			**15.885**	**45.648**	**100.00**	**100.00**	

THF:H_2O =1:3; %ee = 82%

Chromatogram

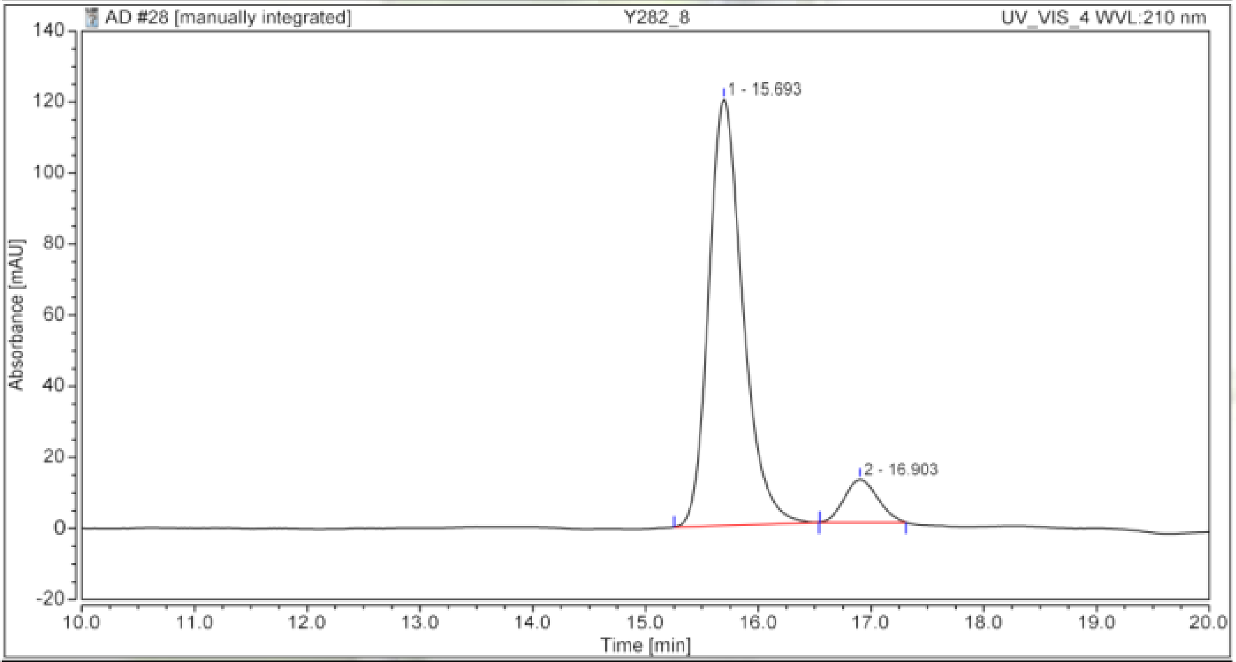

Integration Results

No.	Peak Name	Retention Time min	Area mAU*min	Height mAU	Relative Area %	Relative Height %	Amount n.a.
1		15.693	41.945	119.858	91.02	90.86	n.a.
2		16.903	4.138	12.056	8.98	9.14	n.a.
**Total:**			**46.083**	**131.915**	**100.00**	**100.00**	

THF:H_2O =1:4.5; %ee = 82%

Chromatogram

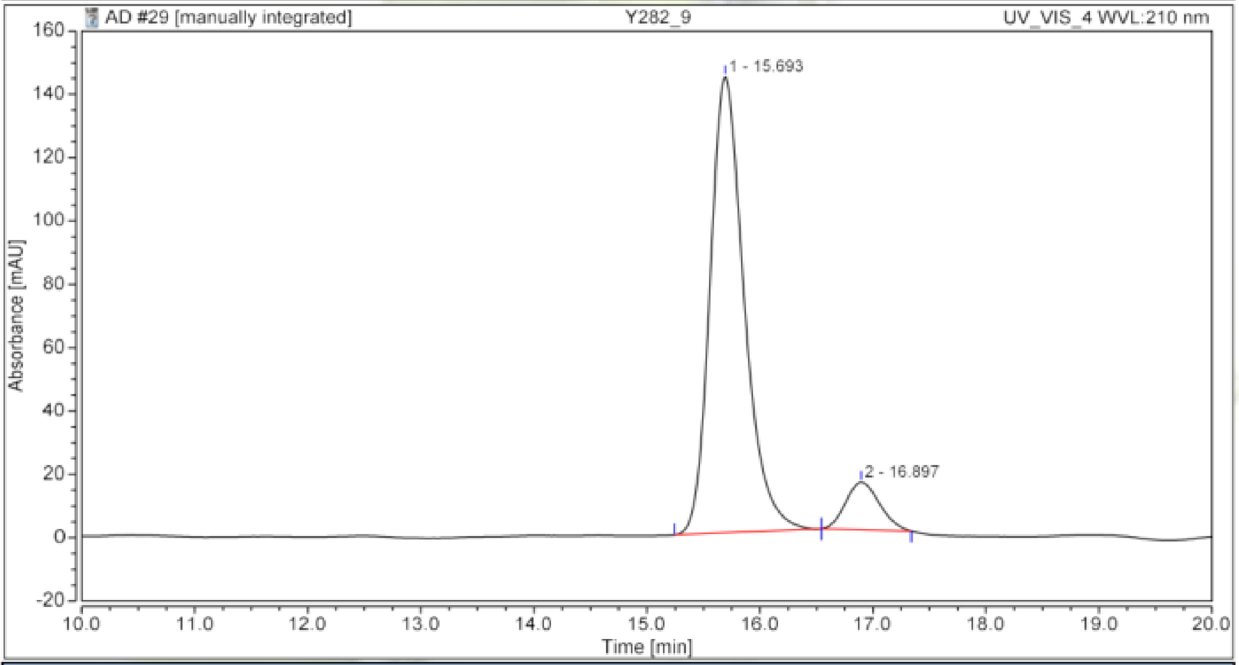

Integration Results

No.	Peak Name	Retention Time min	Area mAU*min	Height mAU	Relative Area %	Relative Height %	Amount n.a.
1		15.693	50.342	144.008	90.57	90.55	n.a.
2		16.897	5.243	15.033	9.43	9.45	n.a.
**Total:**			**55.585**	**159.041**	**100.00**	**100.00**	

THF:H_2O =1:6; %ee = 80%

Chromatogram

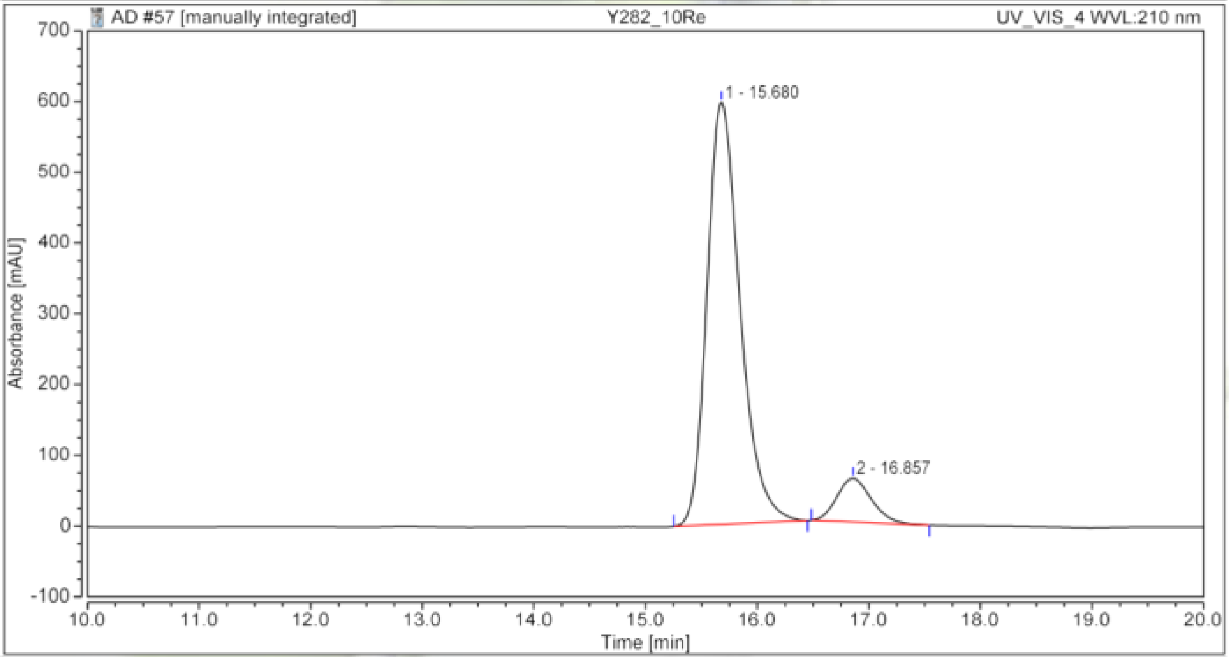

Integration Results

No.	Peak Name	Retention Time min	Area mAU*min	Height mAU	Relative Area %	Relative Height %	Amount n.a.
1		15.680	204.117	596.212	90.25	90.64	n.a.
2		16.857	22.062	61.547	9.75	9.36	n.a.
**Total:**			**226.178**	**657.759**	**100.00**	**100.00**	

(R)-1-(o-tolyl)ethane-1,2-diol

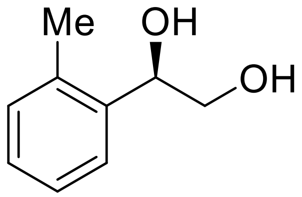

1b

- Column: Chiralpak OD-H.
- Condition: 10% *i*PrOH/Hexane at 0.5 mL/min, enantiomeric excess determined at 210 nm; 14.3 min (R), 17.0 min (S).

THF:H_2O =4.5:1; %ee = 70%

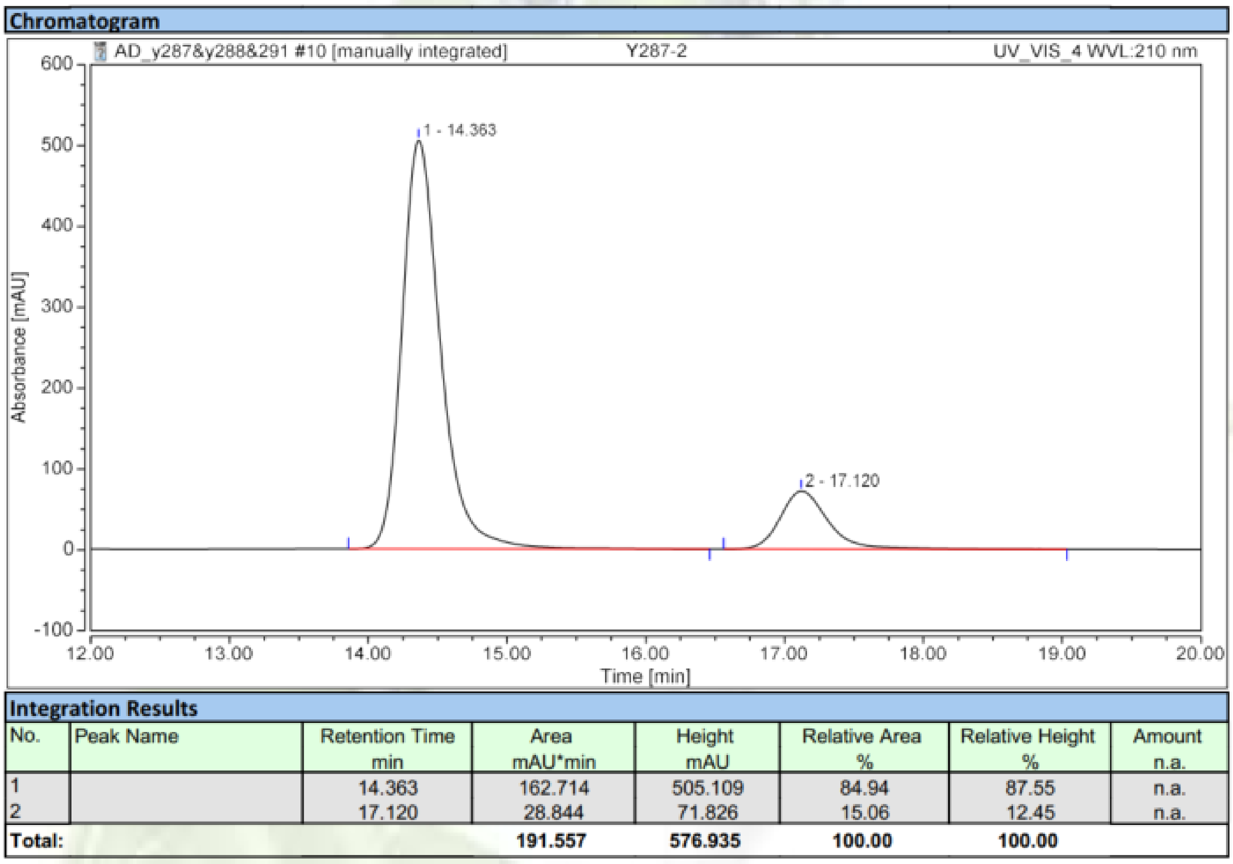

Integration Results

No.	Peak Name	Retention Time min	Area mAU*min	Height mAU	Relative Area %	Relative Height %	Amount n.a.
1		14.363	162.714	505.109	84.94	87.55	n.a.
2		17.120	28.844	71.826	15.06	12.45	n.a.
**Total:**			**191.557**	**576.935**	**100.00**	**100.00**	

THF:H_2O =3:1; %ee = 64%

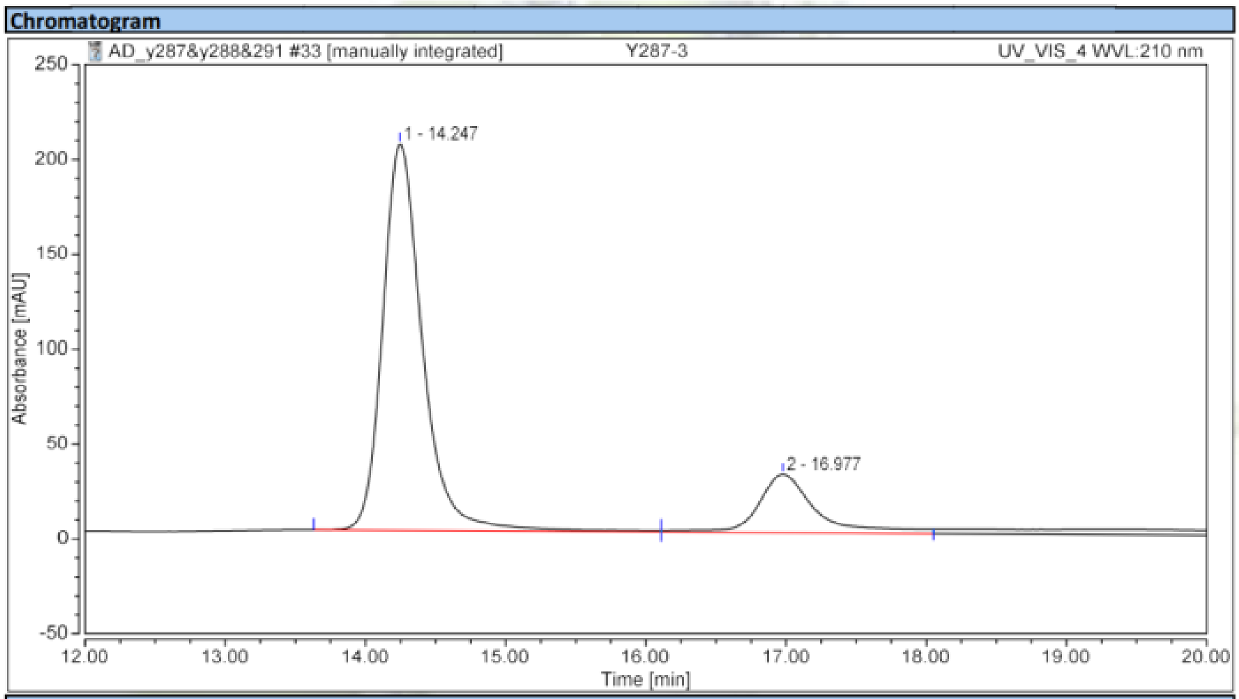

Integration Results

No.	Peak Name	Retention Time min	Area mAU*min	Height mAU	Relative Area %	Relative Height %	Amount n.a.
1		14.247	67.013	203.651	82.07	86.89	n.a.
2		16.977	14.641	30.738	17.93	13.11	n.a.
**Total:**			**81.654**	**234.389**	**100.00**	**100.00**	

THF:H_2O =1.5:1; %ee = 70%

Chromatogram

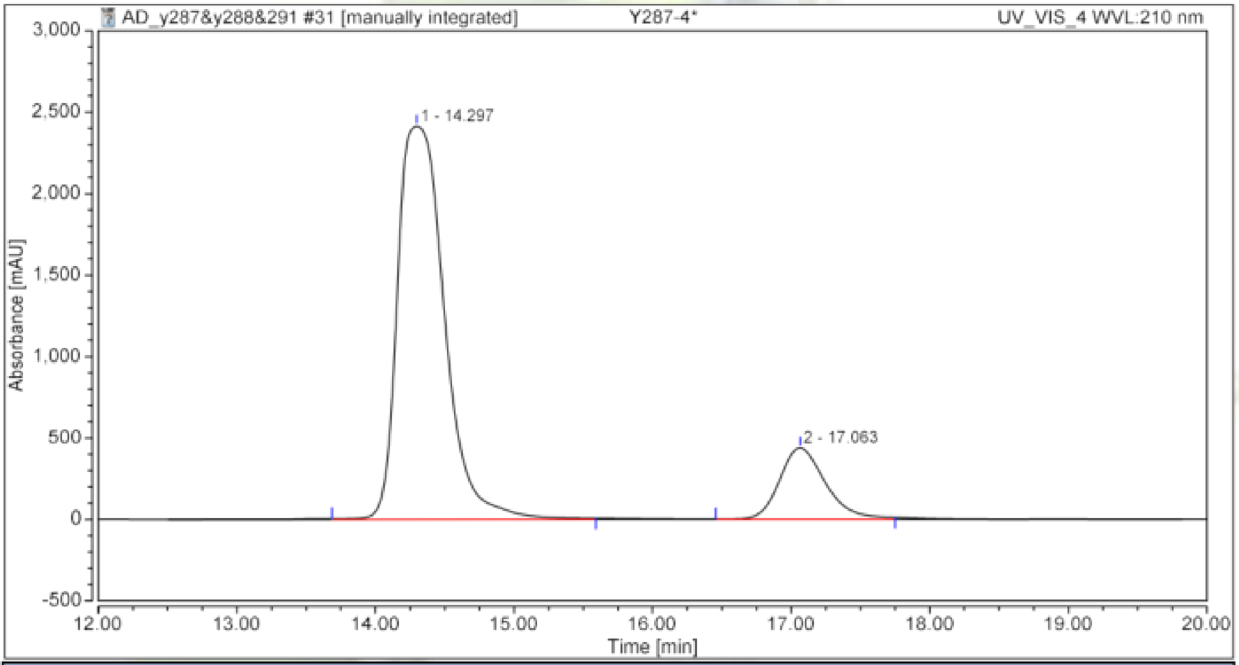

Integration Results

No.	Peak Name	Retention Time<br>min	Area<br>mAU*min	Height<br>mAU	Relative Area<br>%	Relative Height<br>%	Amount<br>n.a.
1		14.297	945.836	2414.958	84.82	84.63	n.a.
2		17.063	169.263	438.541	15.18	15.37	n.a.
**Total:**			**1115.100**	**2853.499**	**100.00**	**100.00**	

THF:H_2O =1:1; %ee = 72%

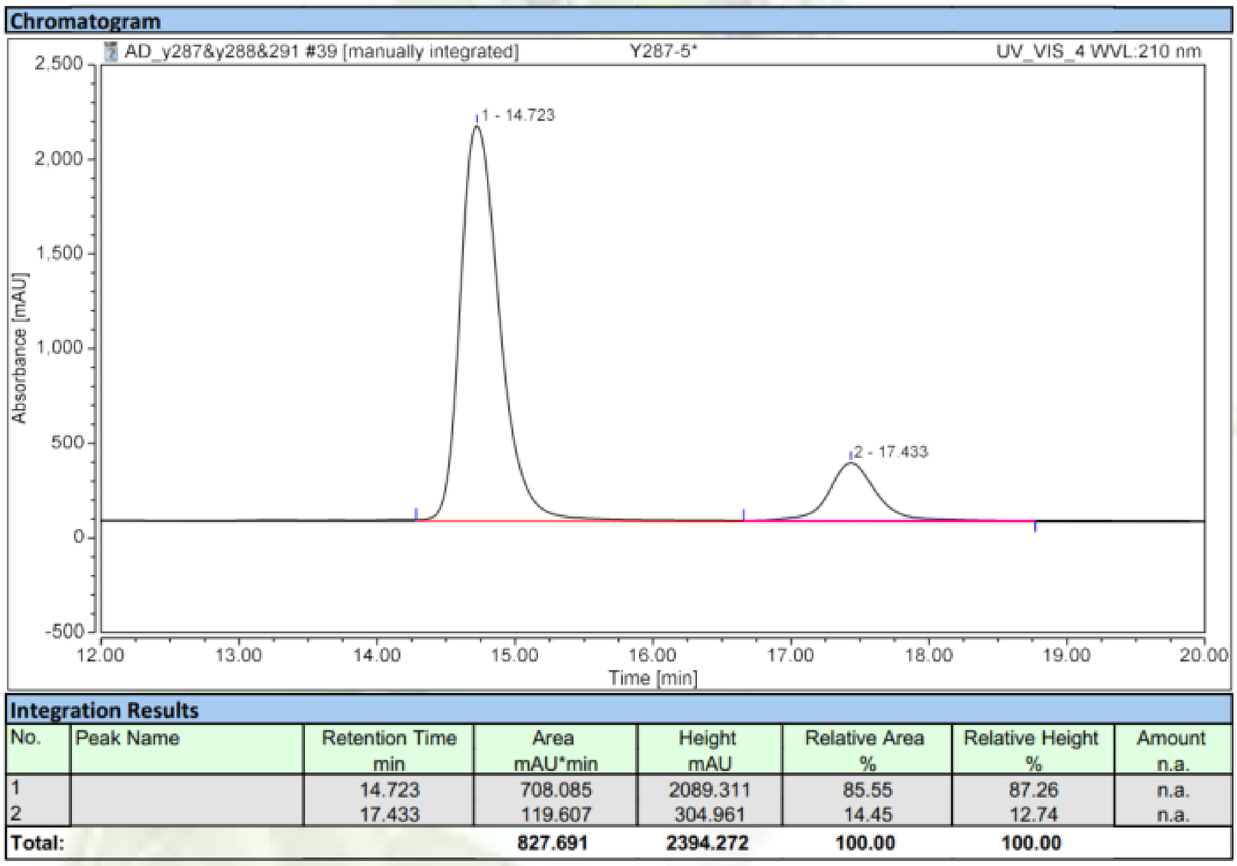

Integration Results

No.	Peak Name	Retention Time min	Area mAU*min	Height mAU	Relative Area %	Relative Height %	Amount n.a.
1		14.723	708.085	2089.311	85.55	87.26	n.a.
2		17.433	119.607	304.961	14.45	12.74	n.a.
**Total:**			**827.691**	**2394.272**	**100.00**	**100.00**	

THF:H_2O =1:1.5; %ee = 74%

Chromatogram

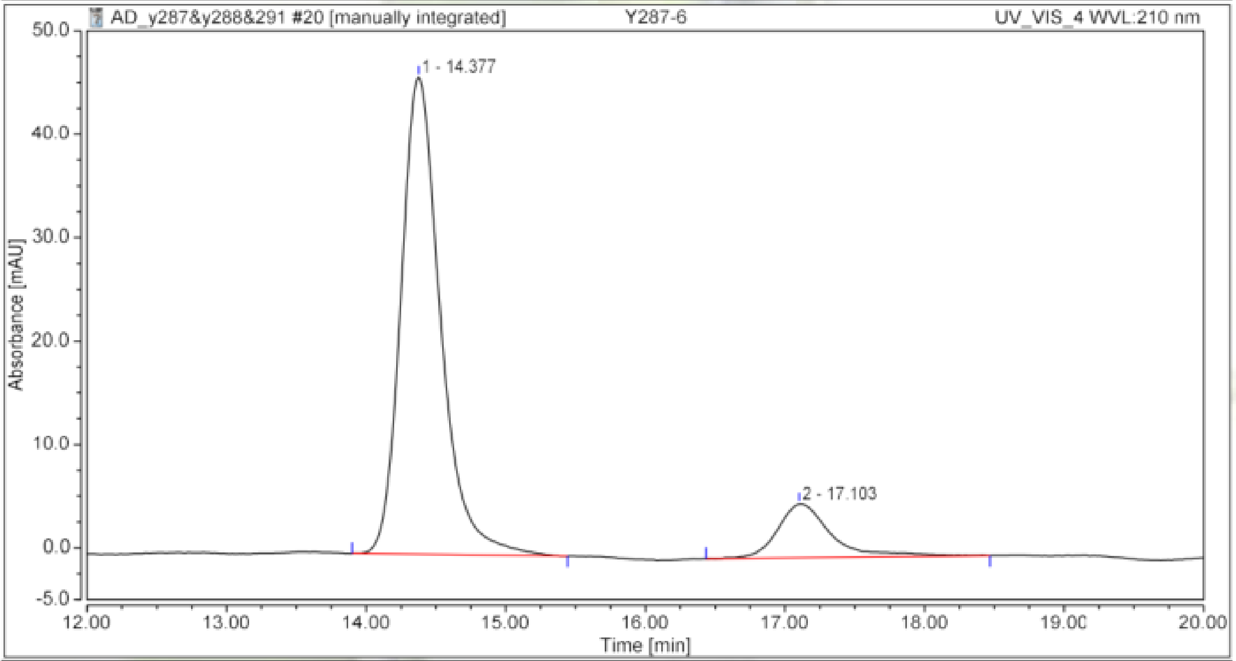

Integration Results

No.	Peak Name	Retention Time min	Area mAU*min	Height mAU	Relative Area %	Relative Height %	Amount n.a.
1		14.377	15.134	46.147	86.71	89.87	n.a.
2		17.103	2.320	5.203	13.29	10.13	n.a.
Total:			17.454	51.350	100.00	100.00	

THF:H_2O =1:3; %ee = 82%

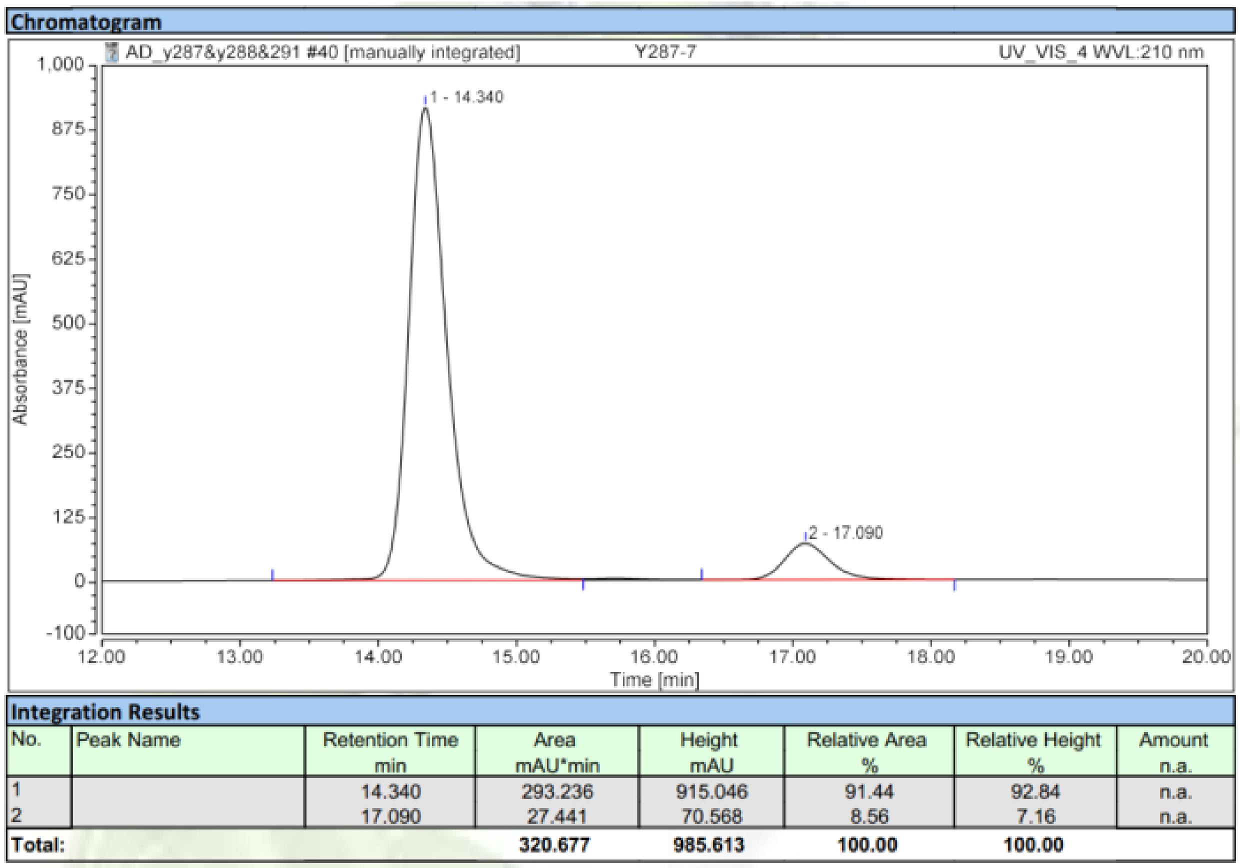

Integration Results

No.	Peak Name	Retention Time min	Area mAU*min	Height mAU	Relative Area %	Relative Height %	Amount n.a.
1		14.340	293.236	915.046	91.44	92.84	n.a.
2		17.090	27.441	70.568	8.56	7.16	n.a.
Total:			320.677	985.613	100.00	100.00	

THF:H_2O =1:4.5; %ee = 84%

Chromatogram

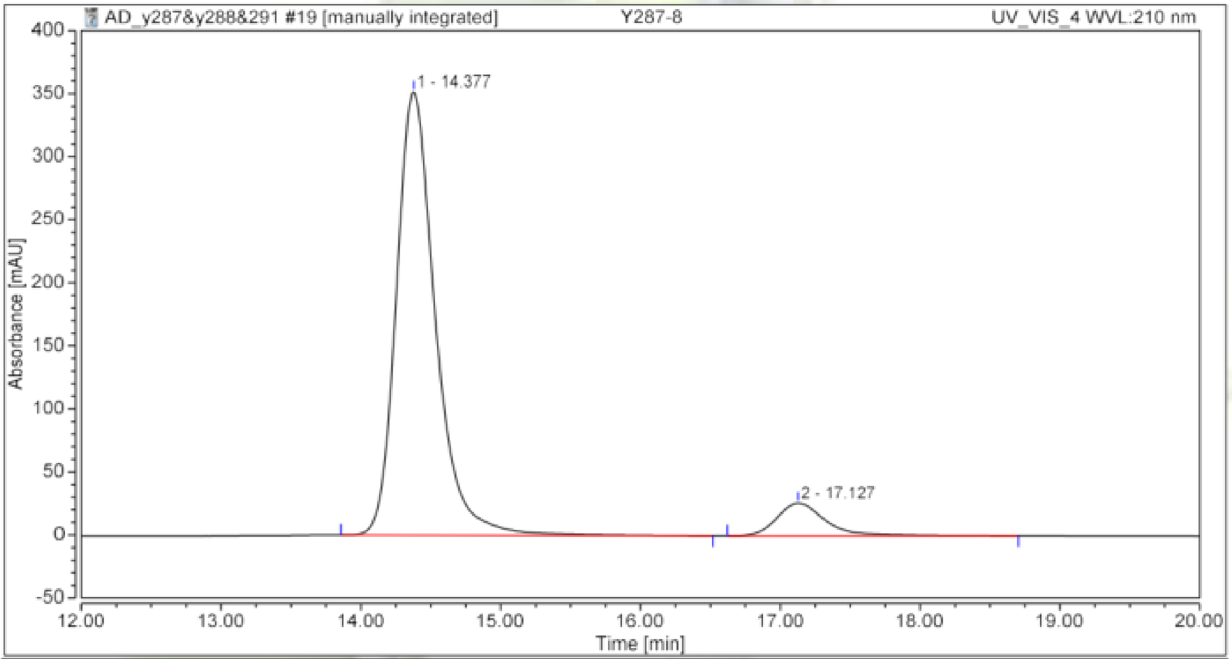

Integration Results

No.	Peak Name	Retention Time min	Area mAU*min	Height mAU	Relative Area %	Relative Height %	Amount n.a.
1		14.377	113.876	351.627	91.51	93.15	n.a.
2		17.127	10.565	25.864	8.49	6.85	n.a.
**Total:**			**124.441**	**377.491**	**100.00**	**100.00**	

THF:H_2O =1:6; %ee = 82%

Chromatogram

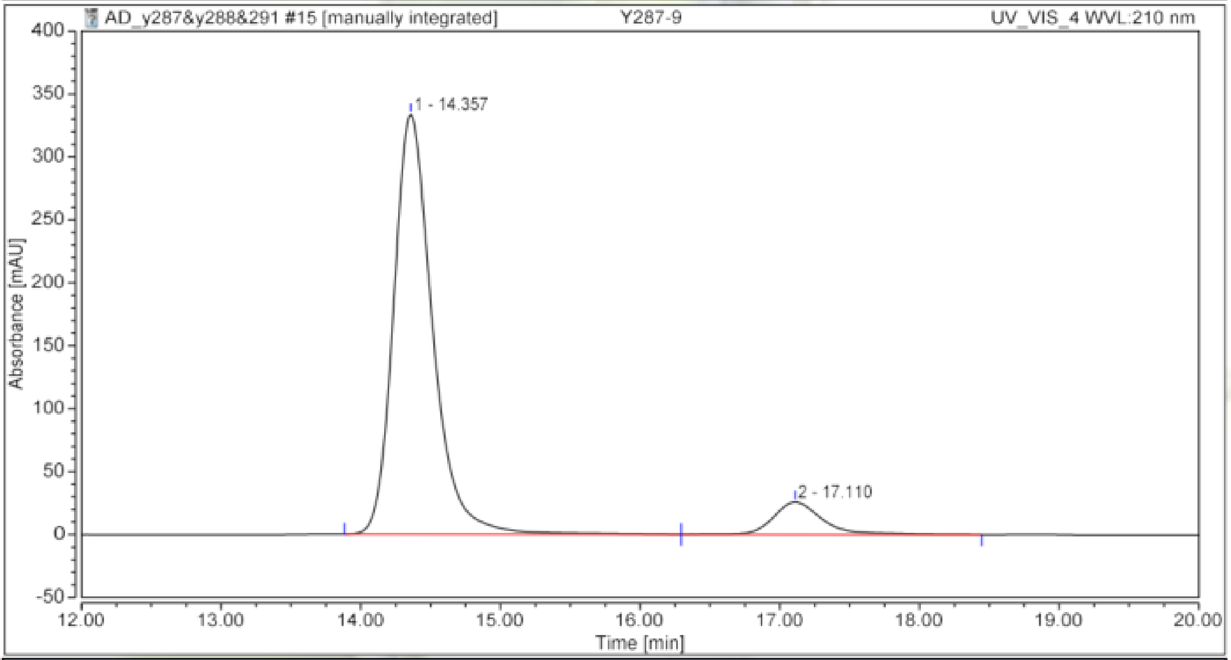

Integration Results

No.	Peak Name	Retention Time min	Area mAU*min	Height mAU	Relative Area %	Relative Height %	Amount n.a.
1		14.357	107.935	333.430	91.15	92.82	n.a.
2		17.110	10.479	25.785	8.85	7.18	n.a.
**Total:**			**118.414**	**359.215**	**100.00**	**100.00**	

(R)-1-(m-tolyl)ethane-1,2-diol

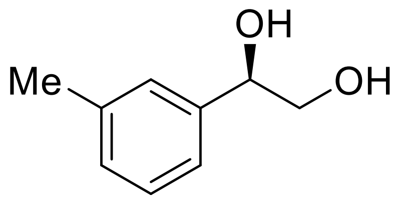

1c

- Column: Chiralpak OD-H.
- Condition: 10% *i*PrOH/Hexane at 0.5 mL/min, enantiomeric excess determined at 210 nm; 14.4 min (R), 15.7 min (S).

THF:H_2O =4.5:1; %ee = 72%

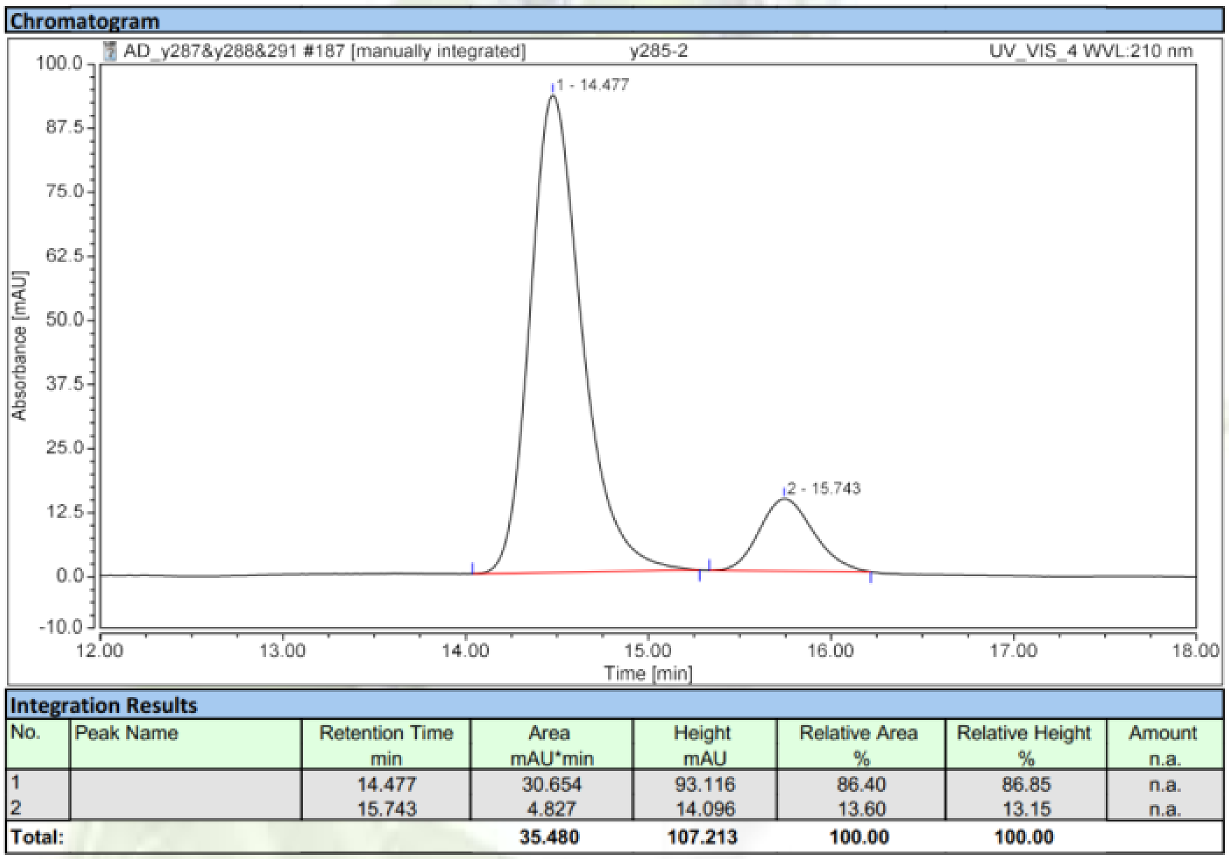

Integration Results

No.	Peak Name	Retention Time min	Area mAU*min	Height mAU	Relative Area %	Relative Height %	Amount n.a.
1		14.477	30.654	93.116	86.40	86.85	n.a.
2		15.743	4.827	14.096	13.60	13.15	n.a.
**Total:**			**35.480**	**107.213**	**100.00**	**100.00**	

THF:H_2O =3:1; %ee = 76%

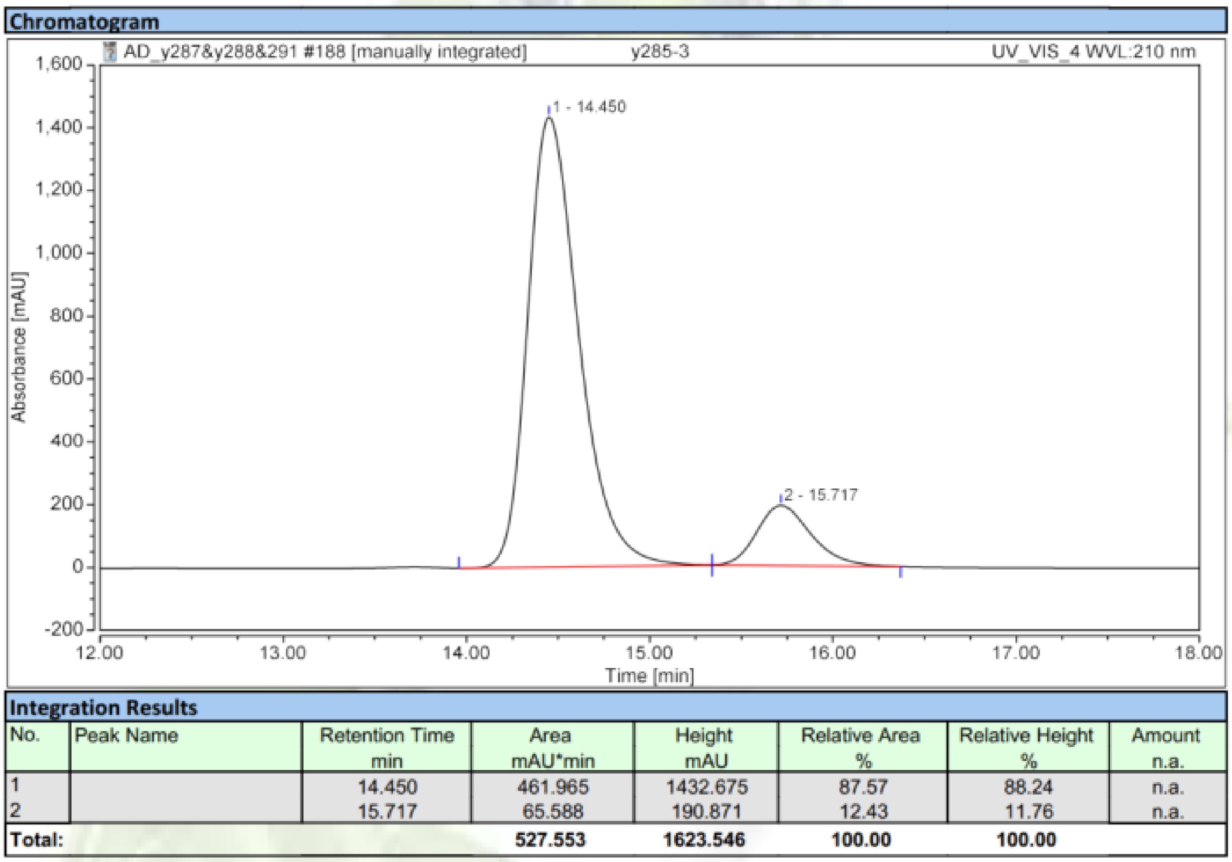

Integration Results

No.	Peak Name	Retention Time min	Area mAU*min	Height mAU	Relative Area %	Relative Height %	Amount n.a.
1		14.450	461.965	1432.675	87.57	88.24	n.a.
2		15.717	65.588	190.871	12.43	11.76	n.a.
**Total:**			**527.553**	**1623.546**	**100.00**	**100.00**	

THF:H_2O =1.5:1; %ee = 78%

Chromatogram

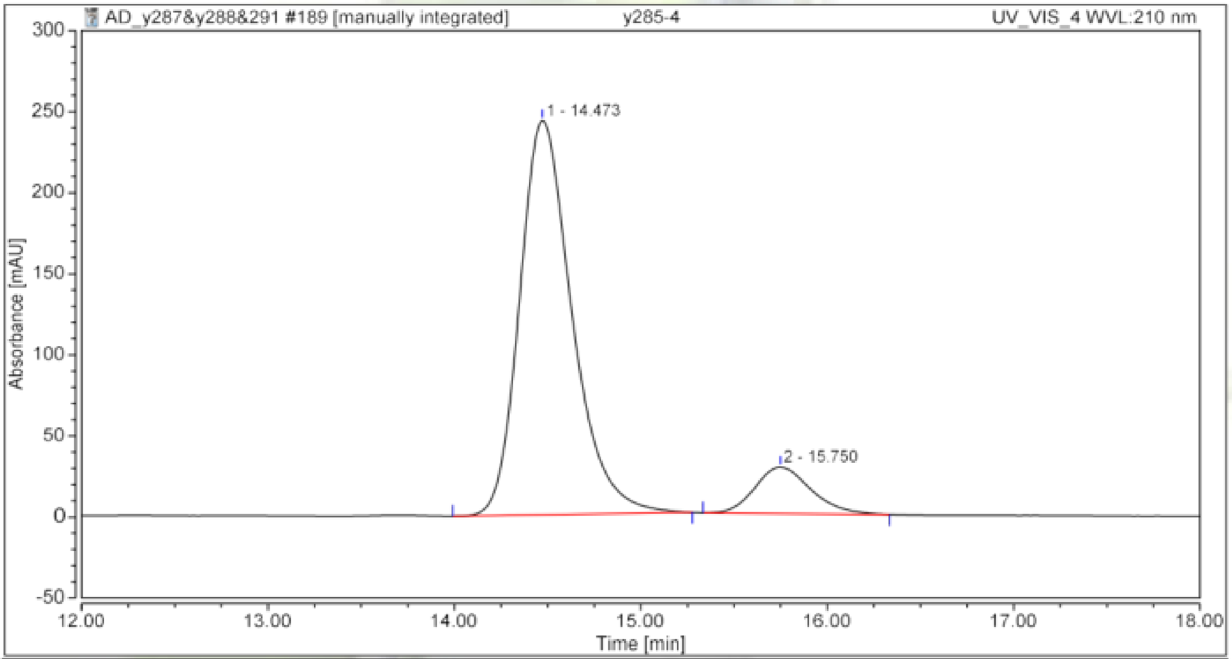

Integration Results

No.	Peak Name	Retention Time min	Area mAU*min	Height mAU	Relative Area %	Relative Height %	Amount n.a.
1		14.473	79.180	243.136	88.85	89.47	n.a.
2		15.750	9.937	28.612	11.15	10.53	n.a.
Total:			89.117	271.748	100.00	100.00	

THF:H_2O =1:1; %ee = 82%

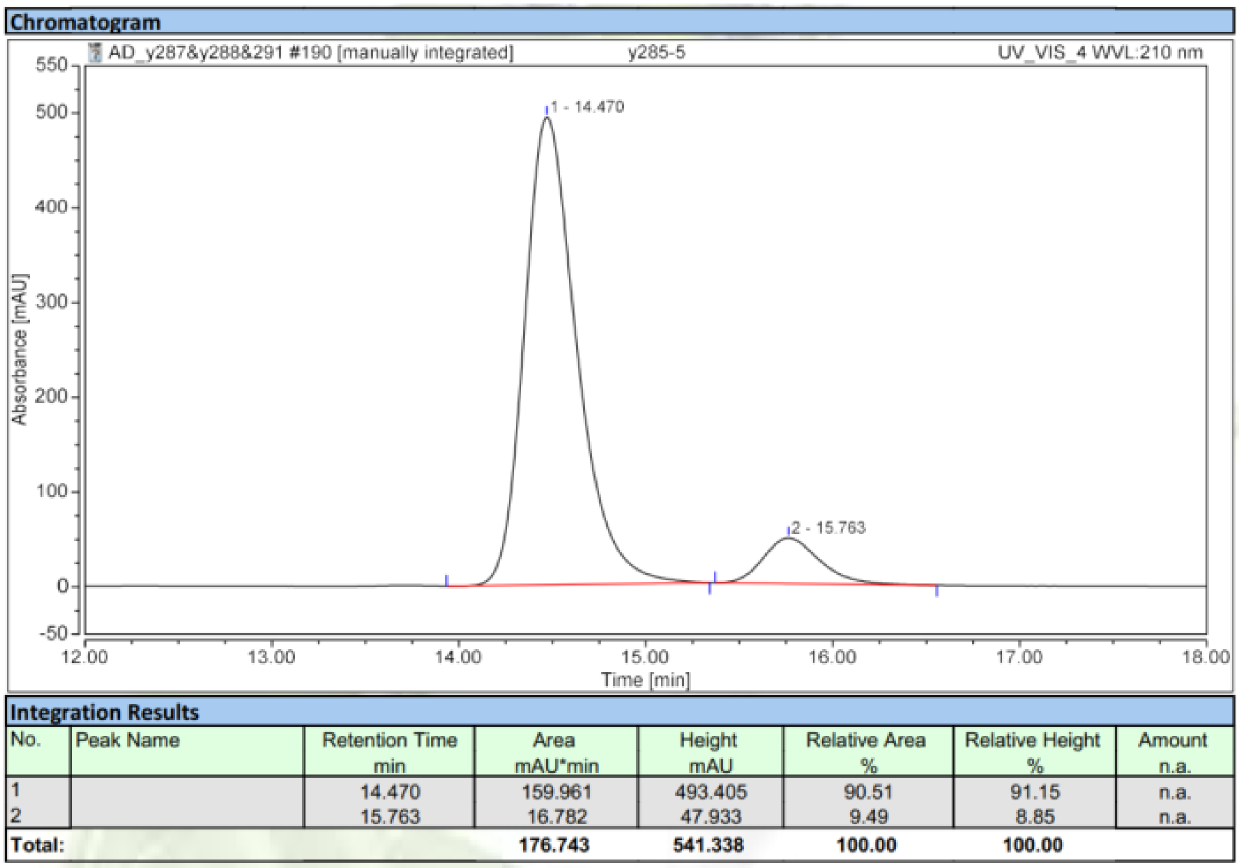

Integration Results

No.	Peak Name	Retention Time min	Area mAU*min	Height mAU	Relative Area %	Relative Height %	Amount n.a.
1		14.470	159.961	493.405	90.51	91.15	n.a.
2		15.763	16.782	47.933	9.49	8.85	n.a.
**Total:**			**176.743**	**541.338**	**100.00**	**100.00**	

THF:H_2O =1:1.5; %ee = 82%

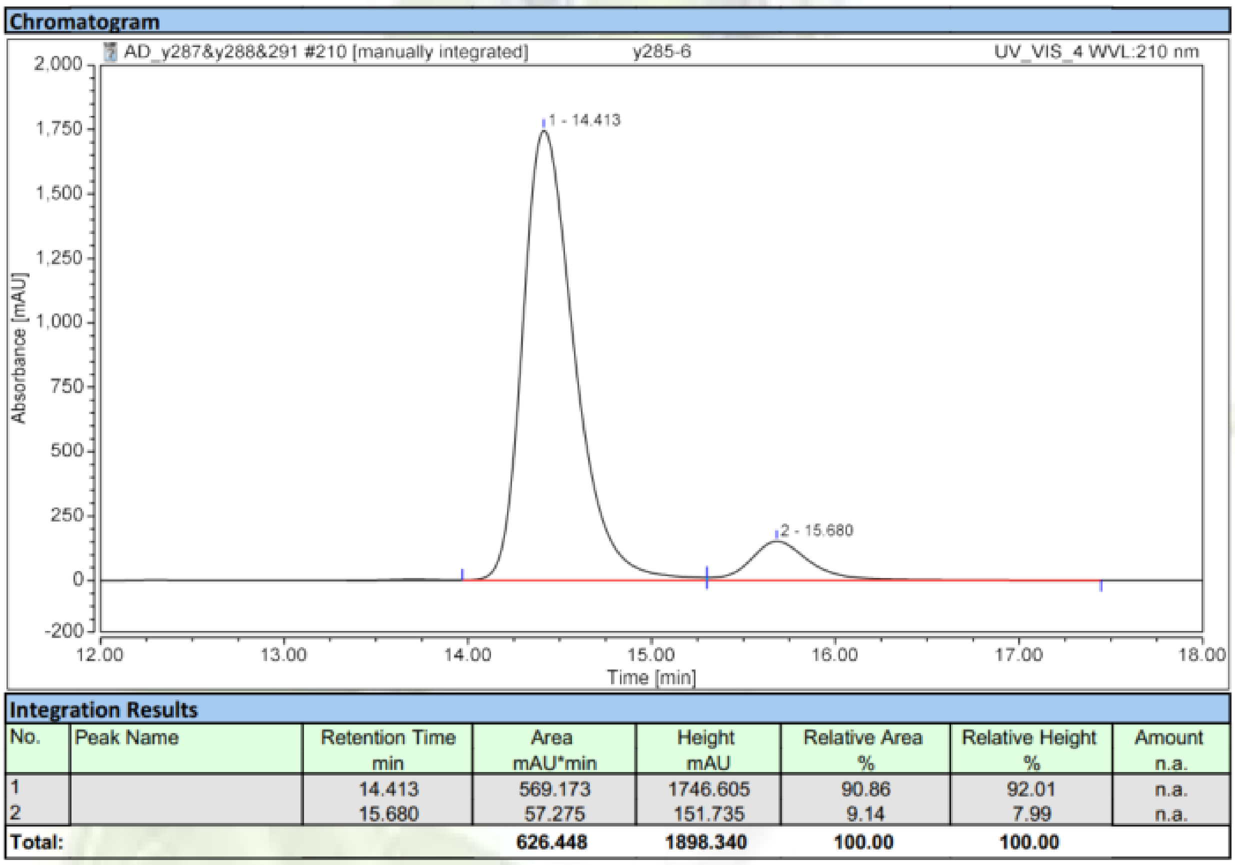

Integration Results

No.	Peak Name	Retention Time min	Area mAU*min	Height mAU	Relative Area %	Relative Height %	Amount n.a.
1		14.413	569.173	1746.605	90.86	92.01	n.a.
2		15.680	57.275	151.735	9.14	7.99	n.a.
**Total:**			**626.448**	**1898.340**	**100.00**	**100.00**	

THF:H_2O =1:3; %ee = 84%

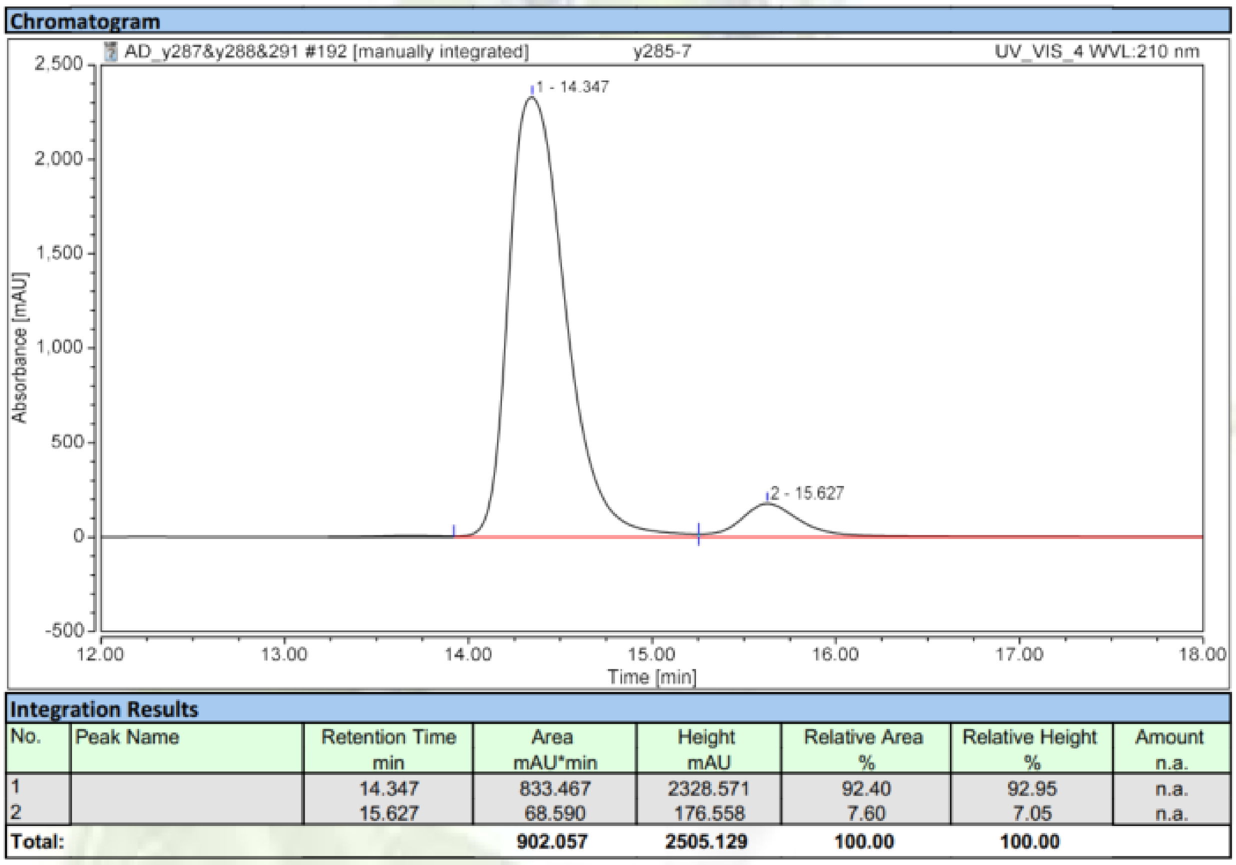

Integration Results

No.	Peak Name	Retention Time min	Area mAU*min	Height mAU	Relative Area %	Relative Height %	Amount n.a.
1		14.347	833.467	2328.571	92.40	92.95	n.a.
2		15.627	68.590	176.558	7.60	7.05	n.a.
**Total:**			**902.057**	**2505.129**	**100.00**	**100.00**	

THF:H_2O =1:4.5; %ee = 84%

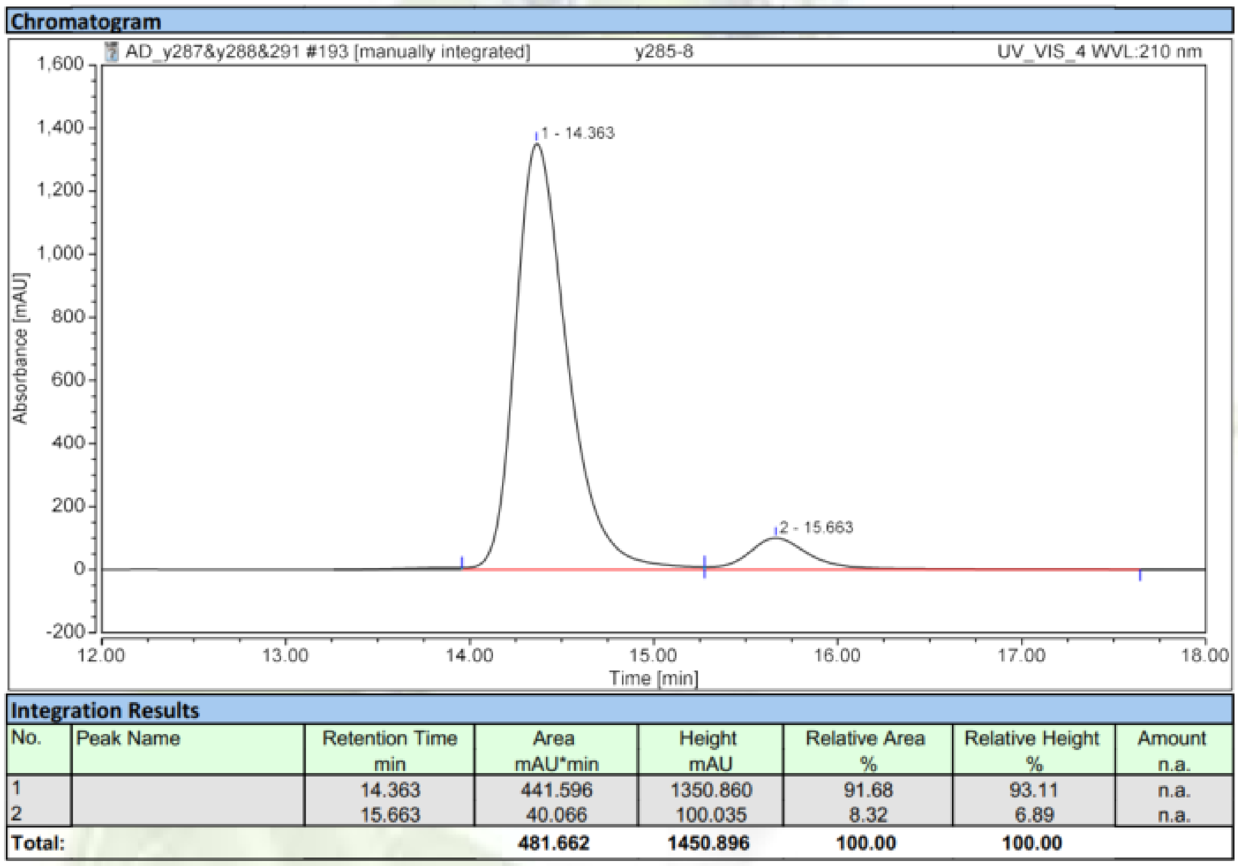

Integration Results

No.	Peak Name	Retention Time min	Area mAU*min	Height mAU	Relative Area %	Relative Height %	Amount n.a.
1		14.363	441.596	1350.860	91.68	93.11	n.a.
2		15.663	40.066	100.035	8.32	6.89	n.a.
**Total:**			**481.662**	**1450.896**	**100.00**	**100.00**	

THF:H_2O =1:6; %ee = 80%

Chromatogram

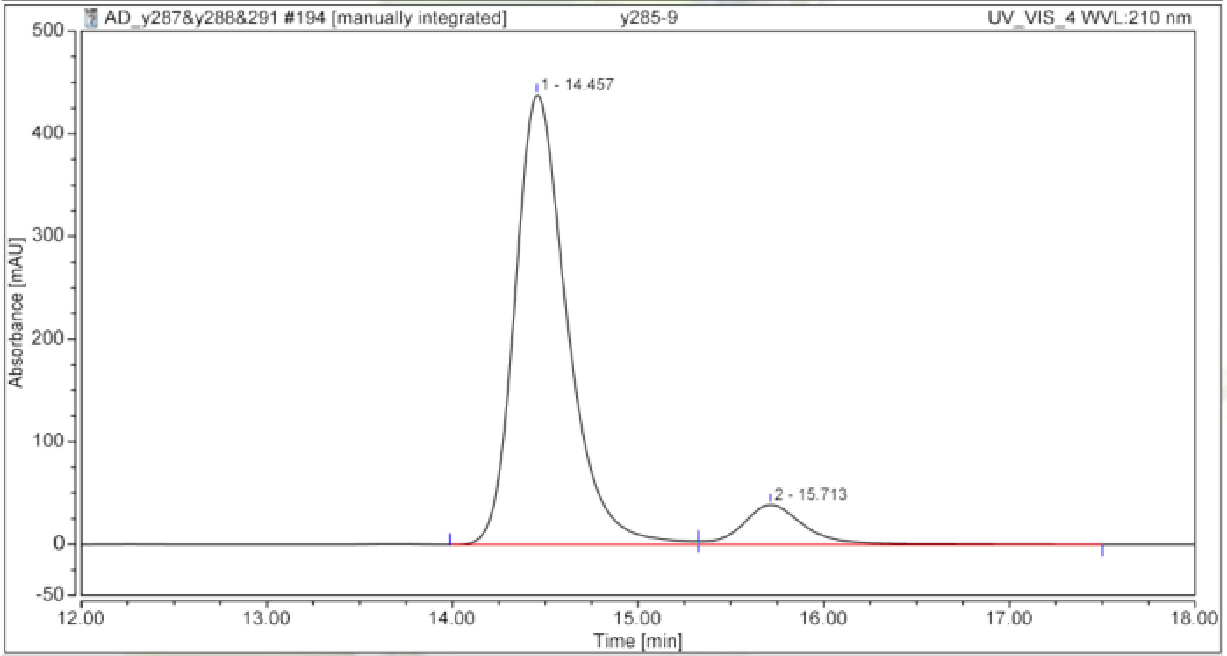

Integration Results

No.	Peak Name	Retention Time min	Area mAU*min	Height mAU	Relative Area %	Relative Height %	Amount n.a.
1		14.457	142.921	438.388	90.17	91.80	n.a.
2		15.713	15.577	39.140	9.83	8.20	n.a.
**Total:**			**158.498**	**477.528**	**100.00**	**100.00**	

(R)-1-(p-tolyl)ethane-1,2-diol

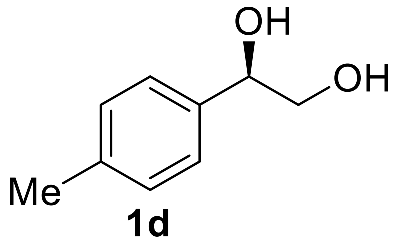

1d

- Column: Chiralpak OD-H.
- Condition: 10% *i*PrOH/Hexane at 0.5 mL/min, enantiomeric excess determined at 210 nm; 14.5 min (R), 16.0 min (S).

THF:H_2O =4.5:1; %ee = 72%

Chromatogram

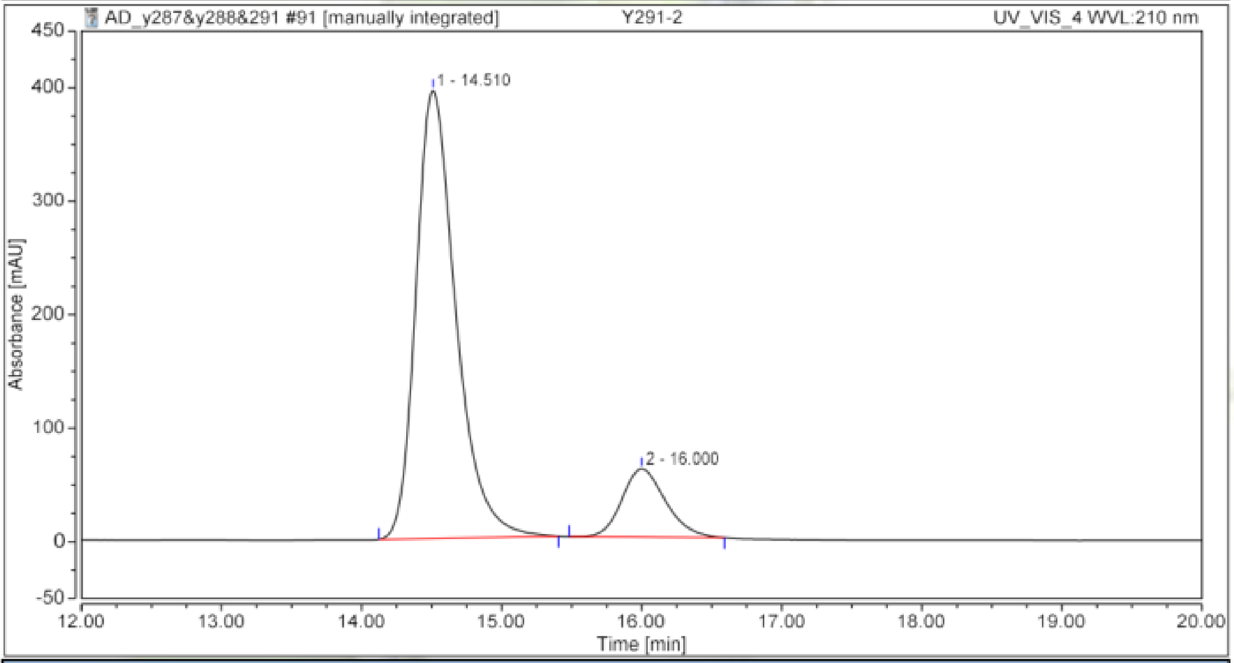

Integration Results

No.	Peak Name	Retention Time min	Area mAU*min	Height mAU	Relative Area %	Relative Height %	Amount n.a.
1		14.510	130.243	394.557	85.71	86.77	n.a.
2		16.000	21.721	60.155	14.29	13.23	n.a.
**Total:**			**151.964**	**454.712**	**100.00**	**100.00**	

THF:H_2O =3:1; %ee = 74%

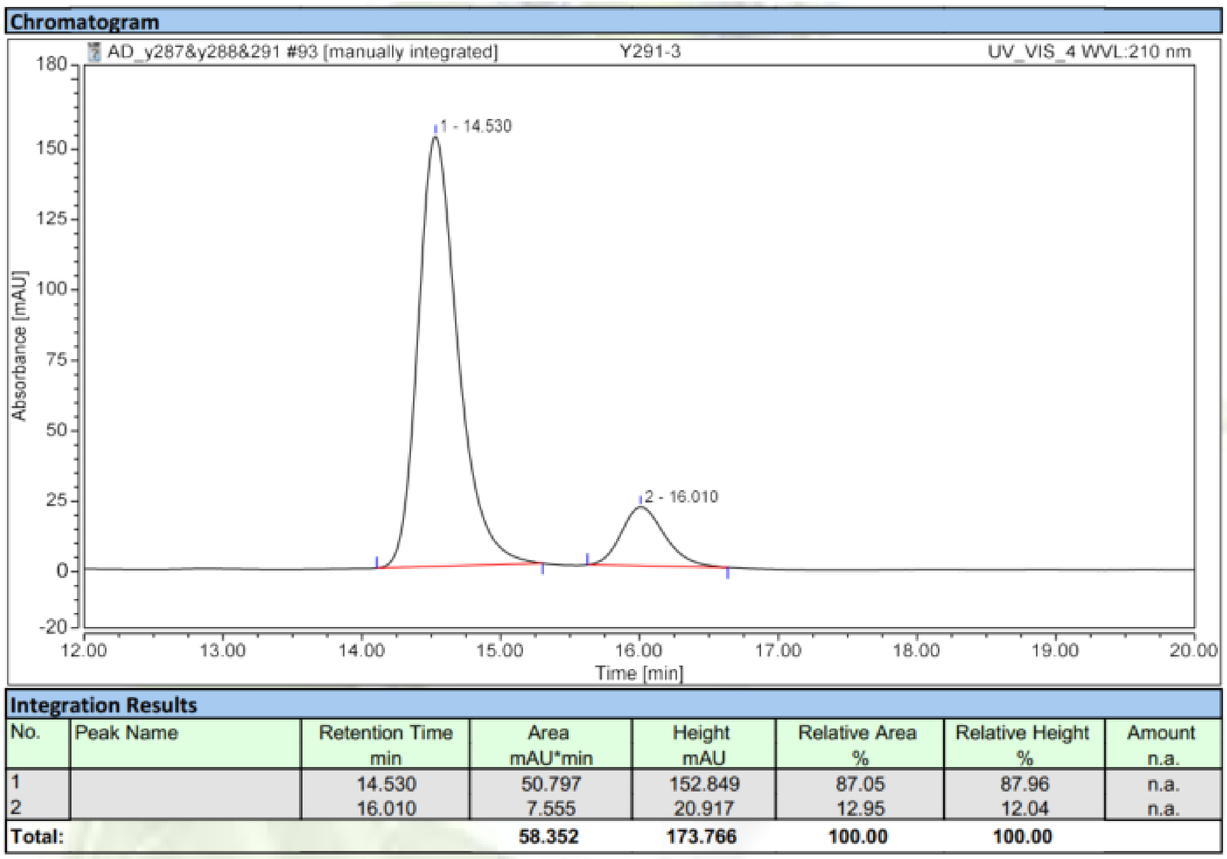

Integration Results

No.	Peak Name	Retention Time min	Area mAU*min	Height mAU	Relative Area %	Relative Height %	Amount n.a.
1		14.530	50.797	152.849	87.05	87.96	n.a.
2		16.010	7.555	20.917	12.95	12.04	n.a.
**Total:**			**58.352**	**173.766**	**100.00**	**100.00**	

THF:H_2O =1.5:1; %ee = 78%

Chromatogram

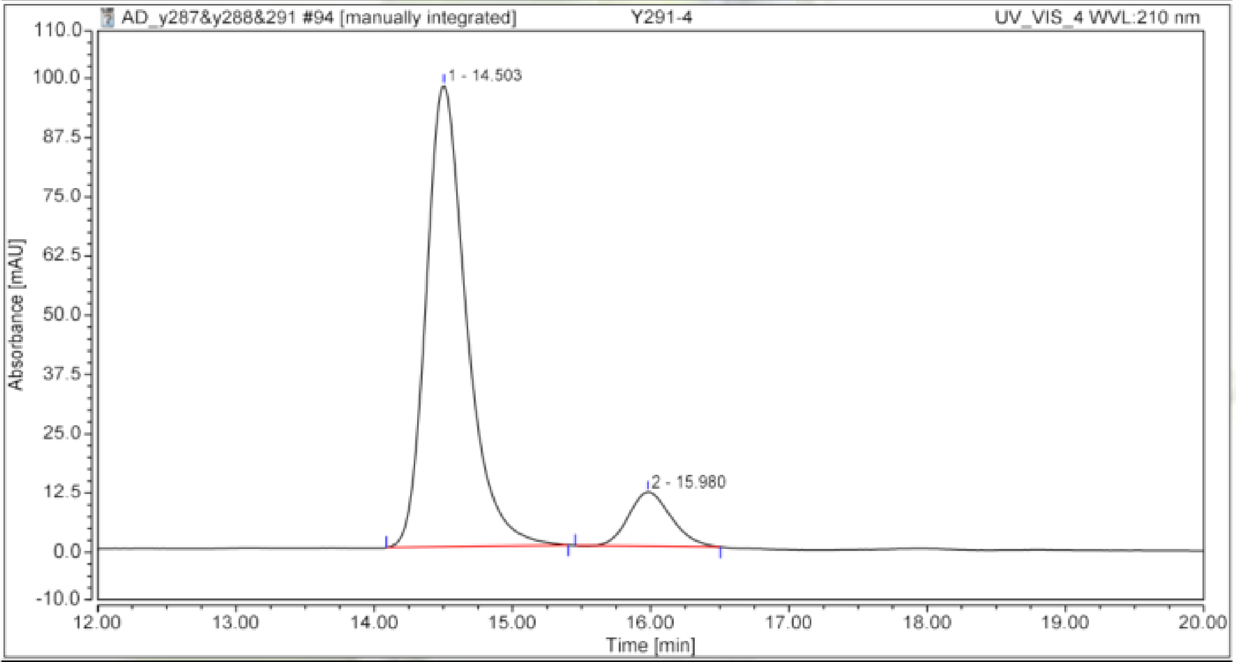

Integration Results

No.	Peak Name	Retention Time min	Area mAU*min	Height mAU	Relative Area %	Relative Height %	Amount n.a.
1		14.503	32.922	97.290	89.01	89.53	n.a.
2		15.980	4.067	11.375	10.99	10.47	n.a.
**Total:**			**36.989**	**108.664**	**100.00**	**100.00**	

THF:H_2O =1:1; %ee = 80%

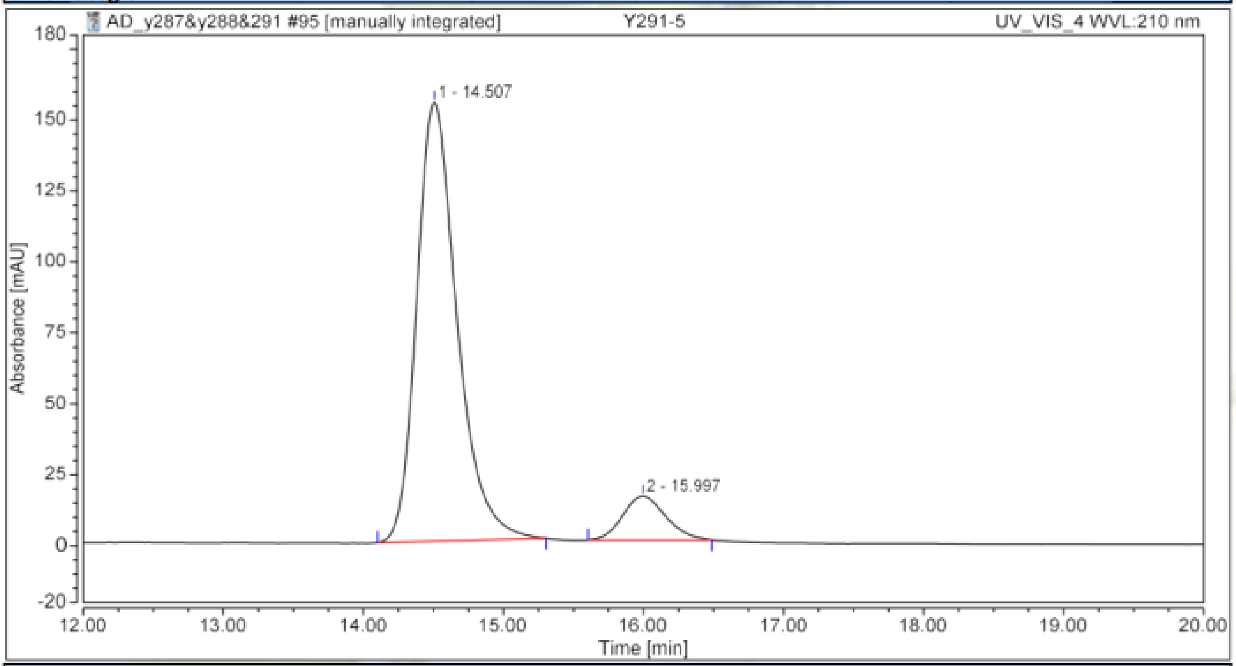

Integration Results

No.	Peak Name	Retention Time min	Area mAU*min	Height mAU	Relative Area %	Relative Height %	Amount n.a.
1		14.507	51.559	154.612	90.40	90.94	n.a.
2		15.997	5.478	15.407	9.60	9.06	n.a.
**Total:**			**57.038**	**170.019**	**100.00**	**100.00**	

THF:H_2O =1:1.5; %ee = 84%

Chromatogram

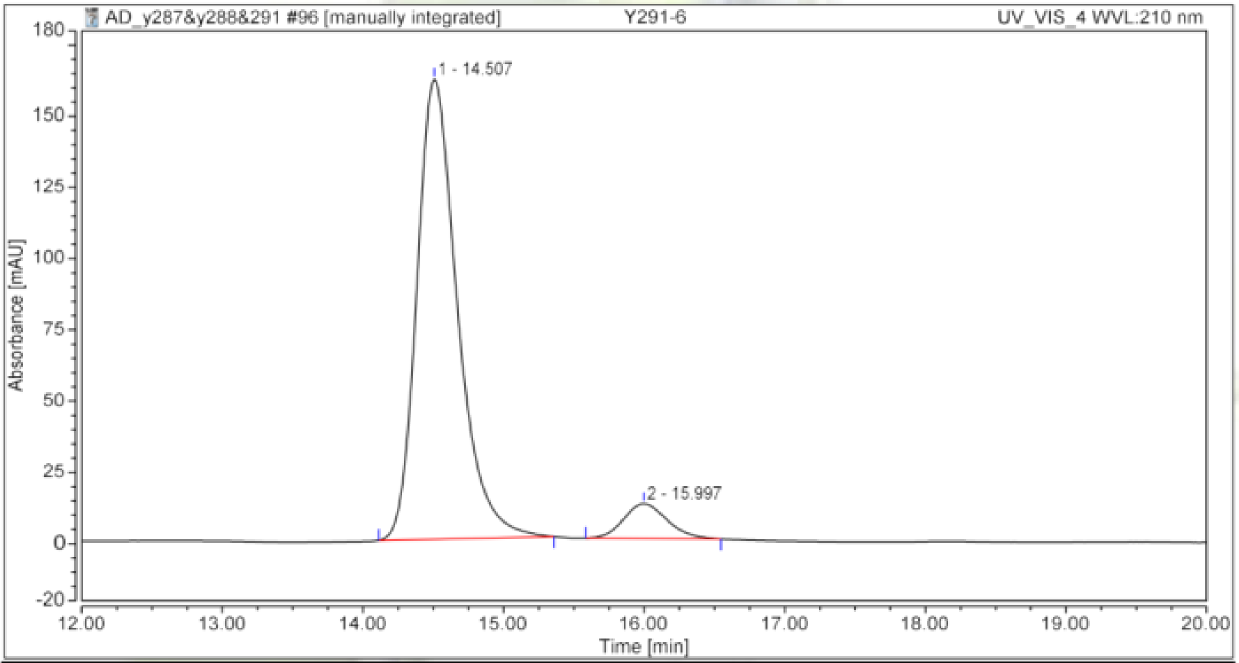

Integration Results

No.	Peak Name	Retention Time min	Area mAU*min	Height mAU	Relative Area %	Relative Height %	Amount n.a.
1		14.507	53.956	161.384	92.49	92.96	n.a.
2		15.997	4.384	12.213	7.51	7.04	n.a.
**Total:**			**58.340**	**173.598**	**100.00**	**100.00**	

THF:H_2O =1:3; %ee = 84%

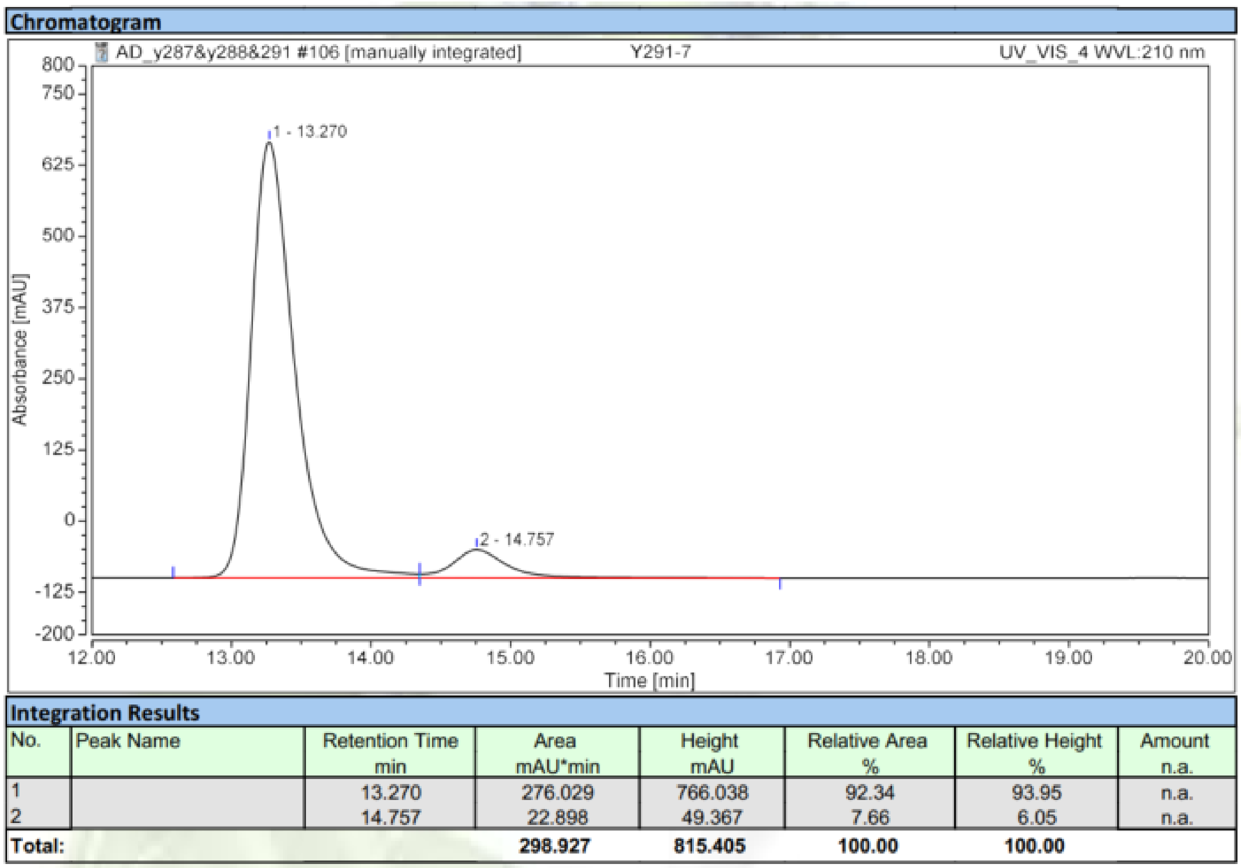

Integration Results

No.	Peak Name	Retention Time min	Area mAU*min	Height mAU	Relative Area %	Relative Height %	Amount n.a.
1		13.270	276.029	766.038	92.34	93.95	n.a.
2		14.757	22.898	49.367	7.66	6.05	n.a.
**Total:**			**298.927**	**815.405**	**100.00**	**100.00**	

THF:H_2O =1:4.5; %ee = 86%

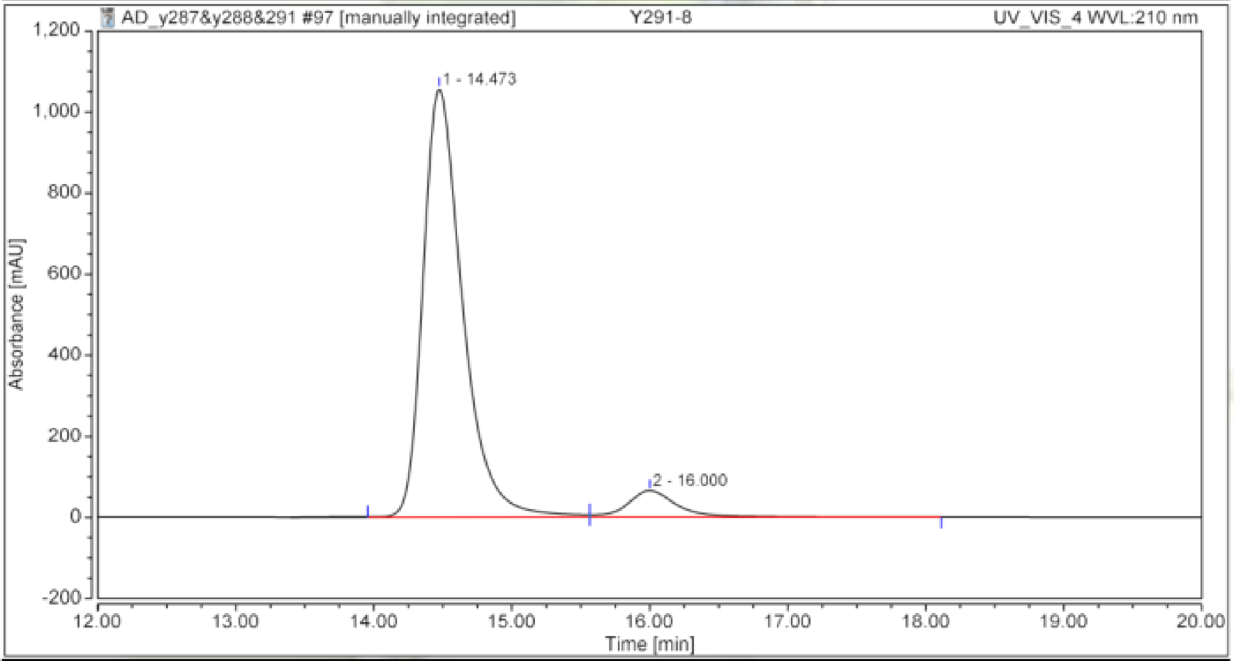

Integration Results

No.	Peak Name	Retention Time min	Area mAU*min	Height mAU	Relative Area %	Relative Height %	Amount n.a.
1		14.473	351.932	1056.162	92.71	94.18	n.a.
2		16.000	27.690	65.308	7.29	5.82	n.a.
Total:			379.623	1121.470	100.00	100.00	

THF:H_2O =1:6; %ee = 88%

Chromatogram

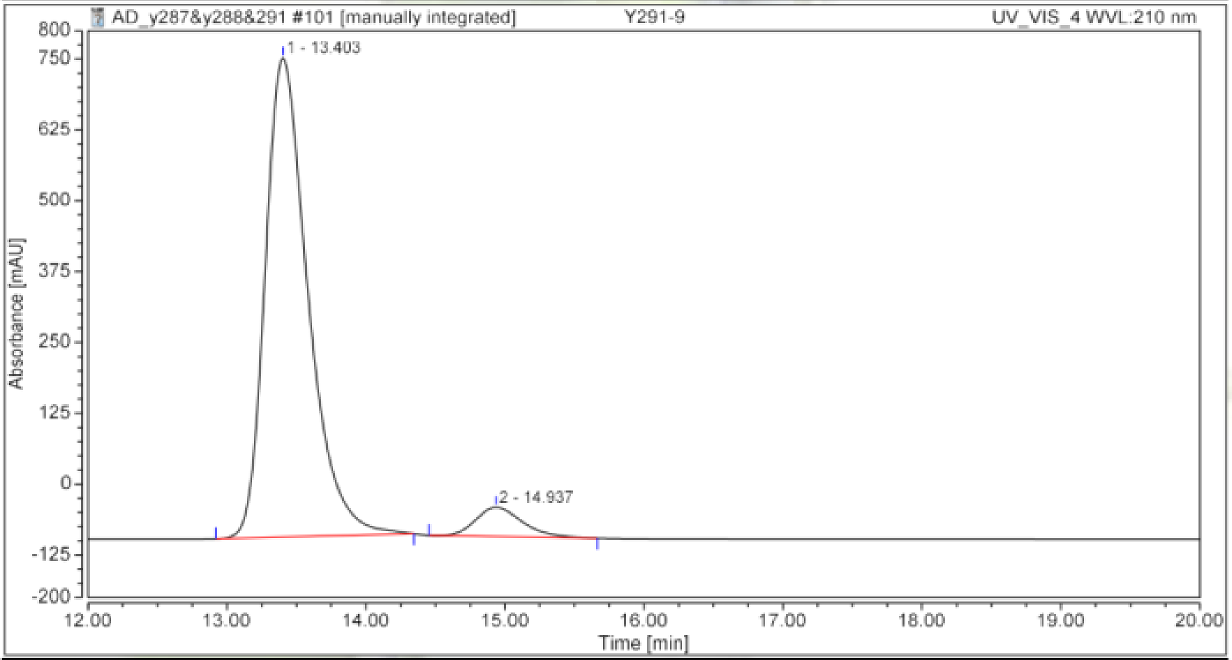

Integration Results

No.	Peak Name	Retention Time min	Area mAU*min	Height mAU	Relative Area %	Relative Height %	Amount n.a.
1		13.403	301.548	845.065	93.81	94.28	n.a.
2		14.937	19.884	51.280	6.19	5.72	n.a.
**Total:**			**321.432**	**896.345**	**100.00**	**100.00**	

(R)-1-(4-methoxyphenyl)ethane-1,2-diol

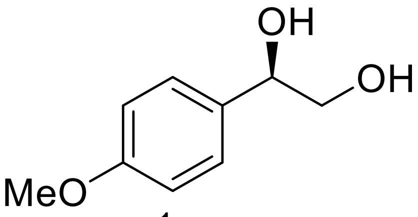

1e

- Column: Chiralpak OD-H.
- Condition: 8% *i*PrOH/Hexane at 0.5 mL/min, enantiomeric excess determined at 210 nm; 25.2 min (R), 27.9 min (S).

THF:H_2O =4.5:1; %ee = 72%

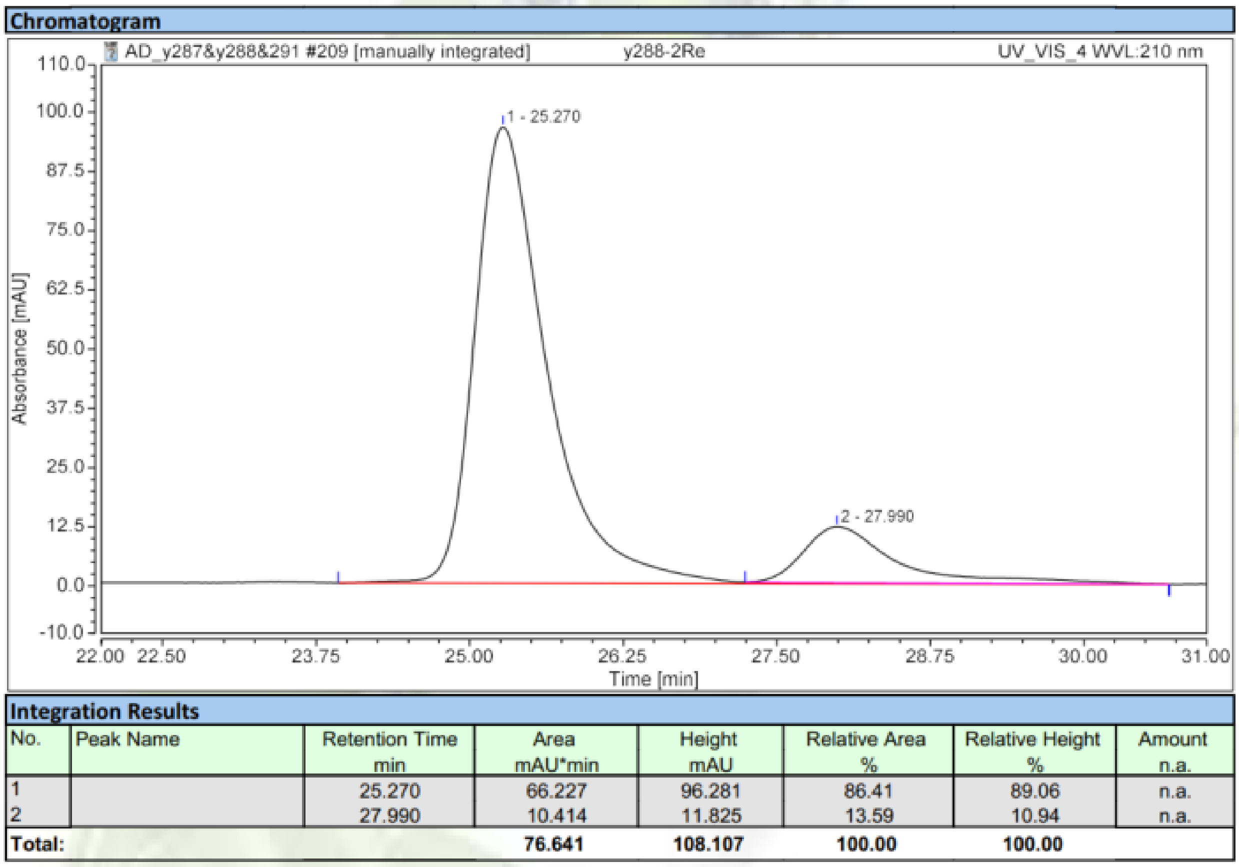

Integration Results

No.	Peak Name	Retention Time min	Area mAU*min	Height mAU	Relative Area %	Relative Height %	Amount n.a.
1		25.270	66.227	96.281	86.41	89.06	n.a.
2		27.990	10.414	11.825	13.59	10.94	n.a.
**Total:**			**76.641**	**108.107**	**100.00**	**100.00**	

THF:H_2O =3:1; %ee = 78%

Chromatogram

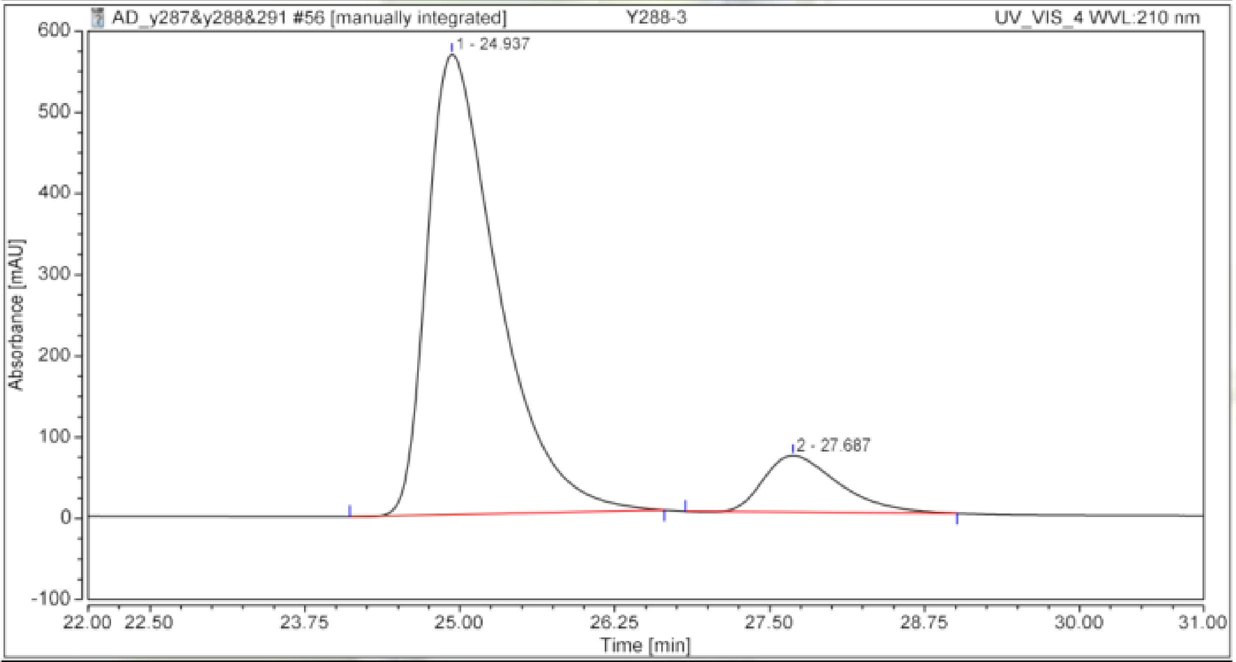

Integration Results

No.	Peak Name	Retention Time min	Area mAU*min	Height mAU	Relative Area %	Relative Height %	Amount n.a.
1		24.937	374.668	566.266	88.54	89.08	n.a.
2		27.687	48.515	69.444	11.46	10.92	n.a.
**Total:**			**423.183**	**635.711**	**100.00**	**100.00**	

THF:H_2O =1.5:1; %ee = 82%

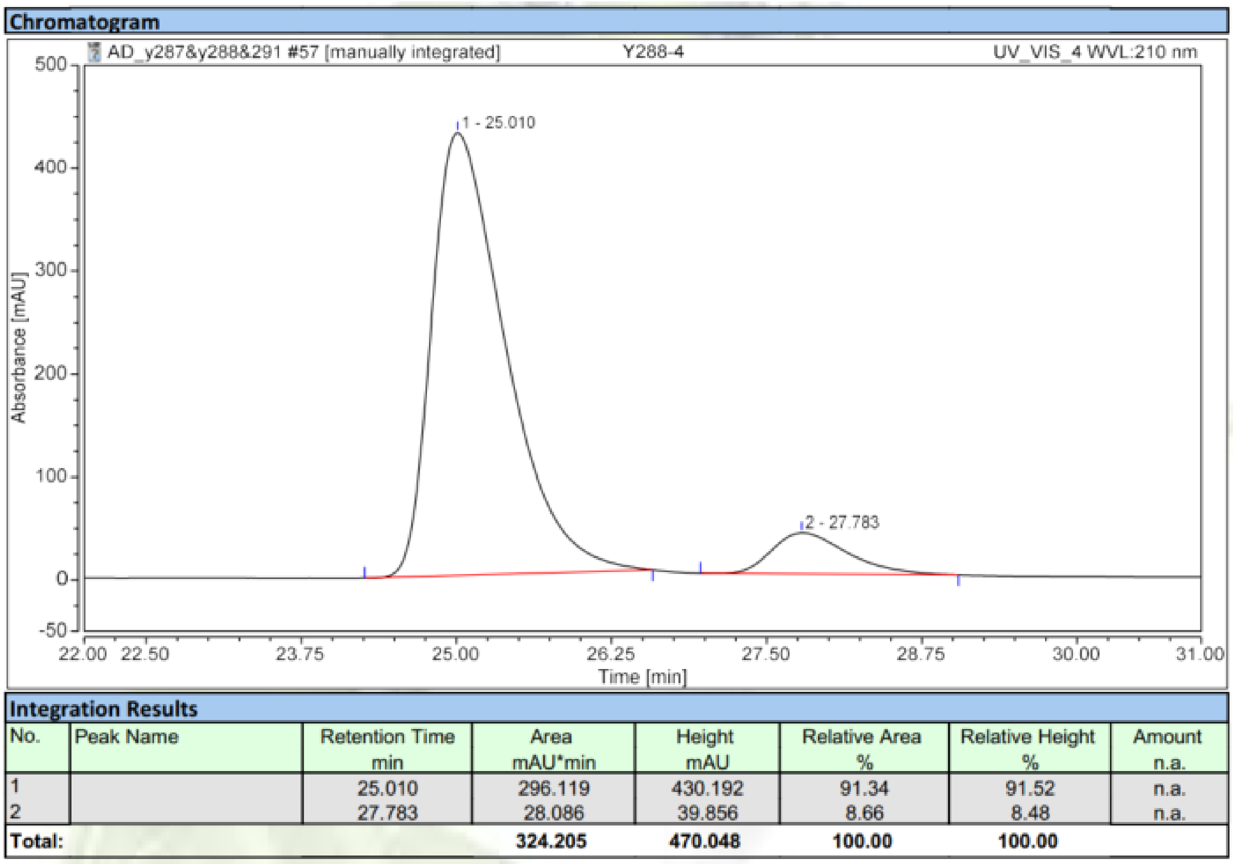

Integration Results

No.	Peak Name	Retention Time min	Area mAU*min	Height mAU	Relative Area %	Relative Height %	Amount n.a.
1		25.010	296.119	430.192	91.34	91.52	n.a.
2		27.783	28.086	39.856	8.66	8.48	n.a.
**Total:**			**324.205**	**470.048**	**100.00**	**100.00**	

THF:H_2O =1:1; %ee = 84%

Chromatogram

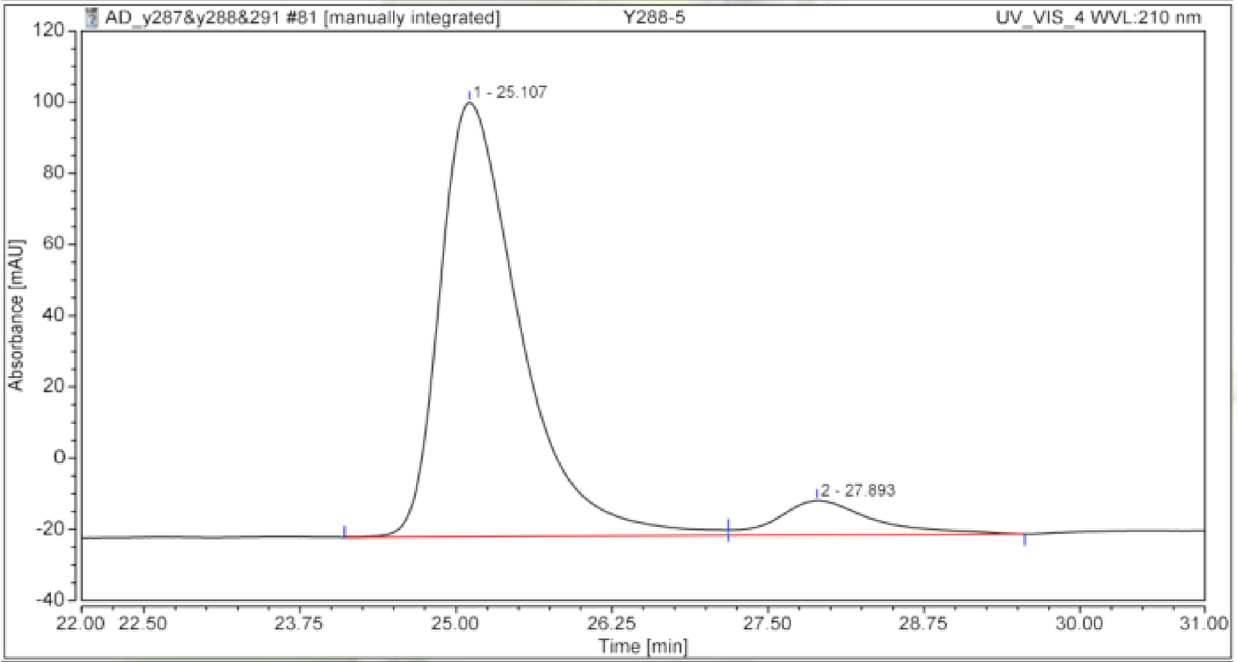

Integration Results

No.	Peak Name	Retention Time min	Area mAU*min	Height mAU	Relative Area %	Relative Height %	Amount n.a.
1		25.107	90.595	121.895	91.51	92.68	n.a.
2		27.893	8.406	9.627	8.49	7.32	n.a.
**Total:**			**99.001**	**131.523**	**100.00**	**100.00**	

THF:H_2O =1:1.5; %ee = 86%

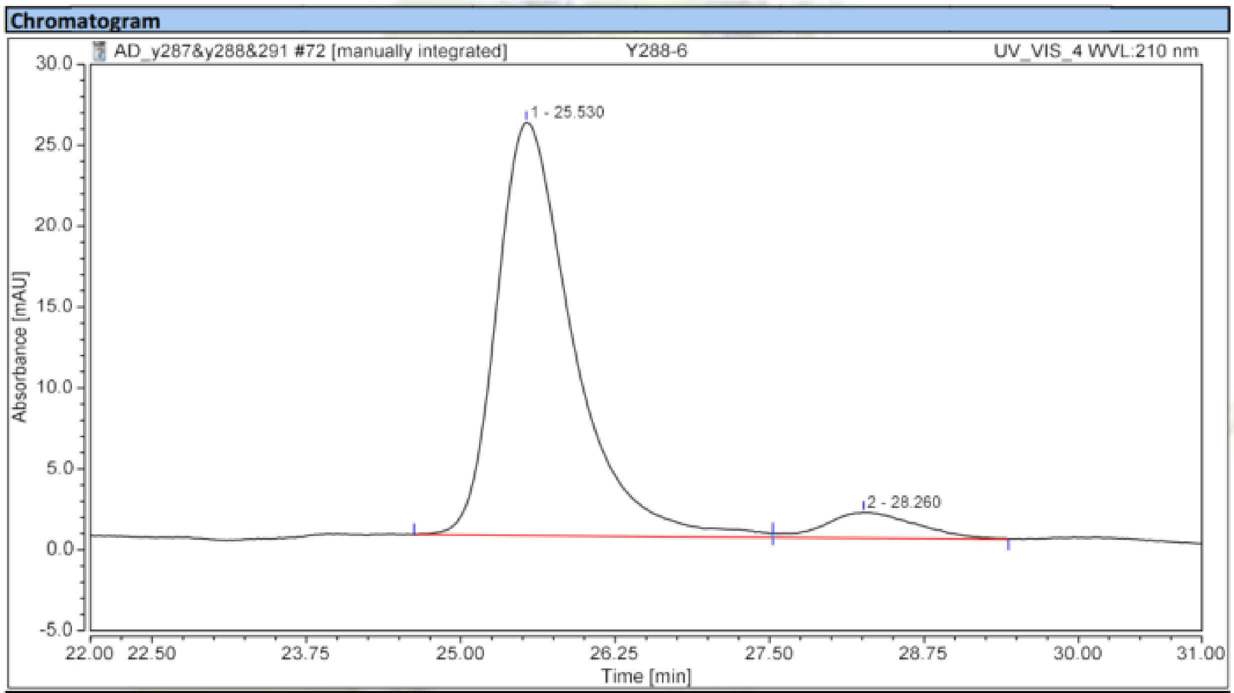

Integration Results

No.	Peak Name	Retention Time min	Area mAU*min	Height mAU	Relative Area %	Relative Height %	Amount n.a.
1		25.530	18.284	25.526	93.16	94.18	n.a.
2		28.260	1.342	1.577	6.84	5.82	n.a.
**Total:**			**19.627**	**27.103**	**100.00**	**100.00**	

THF:H_2O =1:3; %ee = 88%

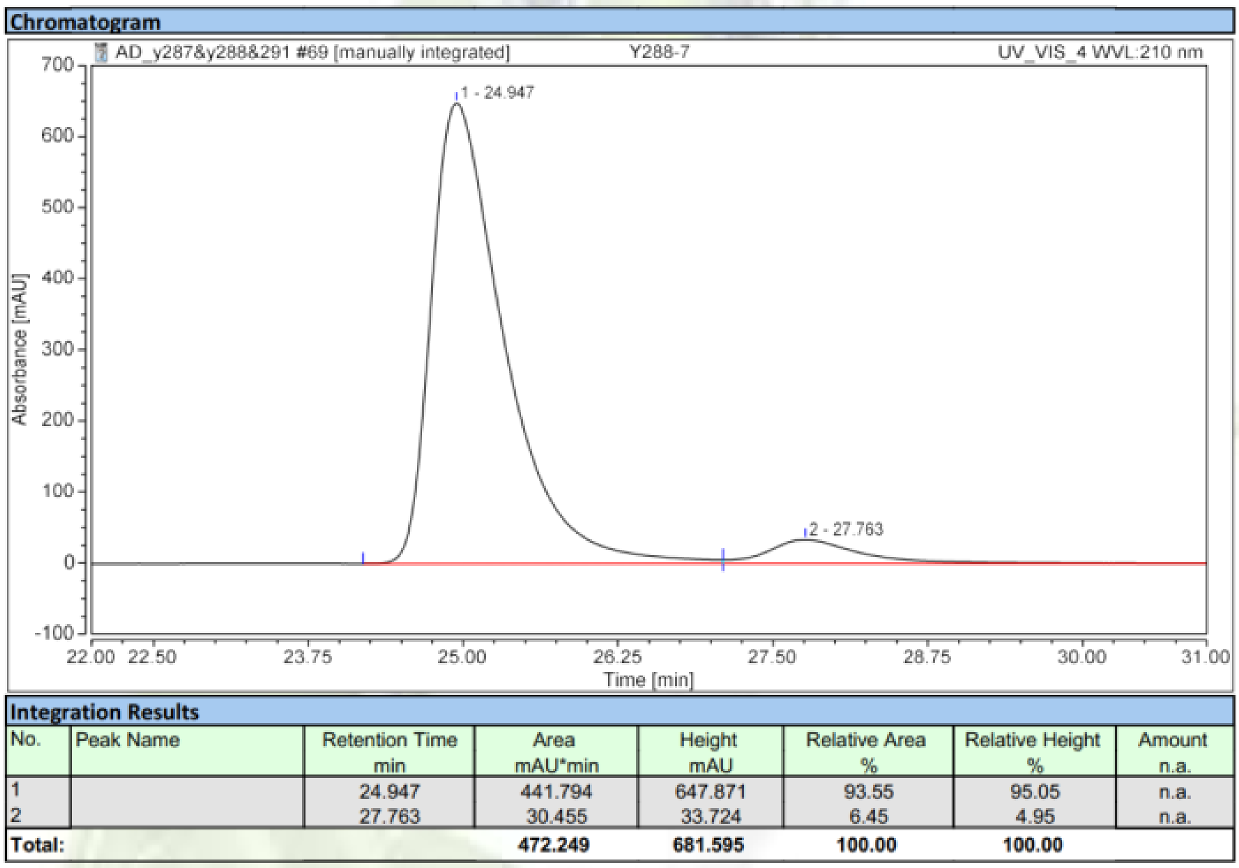

Integration Results

No.	Peak Name	Retention Time min	Area mAU*min	Height mAU	Relative Area %	Relative Height %	Amount n.a.
1		24.947	441.794	647.871	93.55	95.05	n.a.
2		27.763	30.455	33.724	6.45	4.95	n.a.
**Total:**			**472.249**	**681.595**	**100.00**	**100.00**	

THF:H_2O =1:4.5; %ee = 86%

Chromatogram

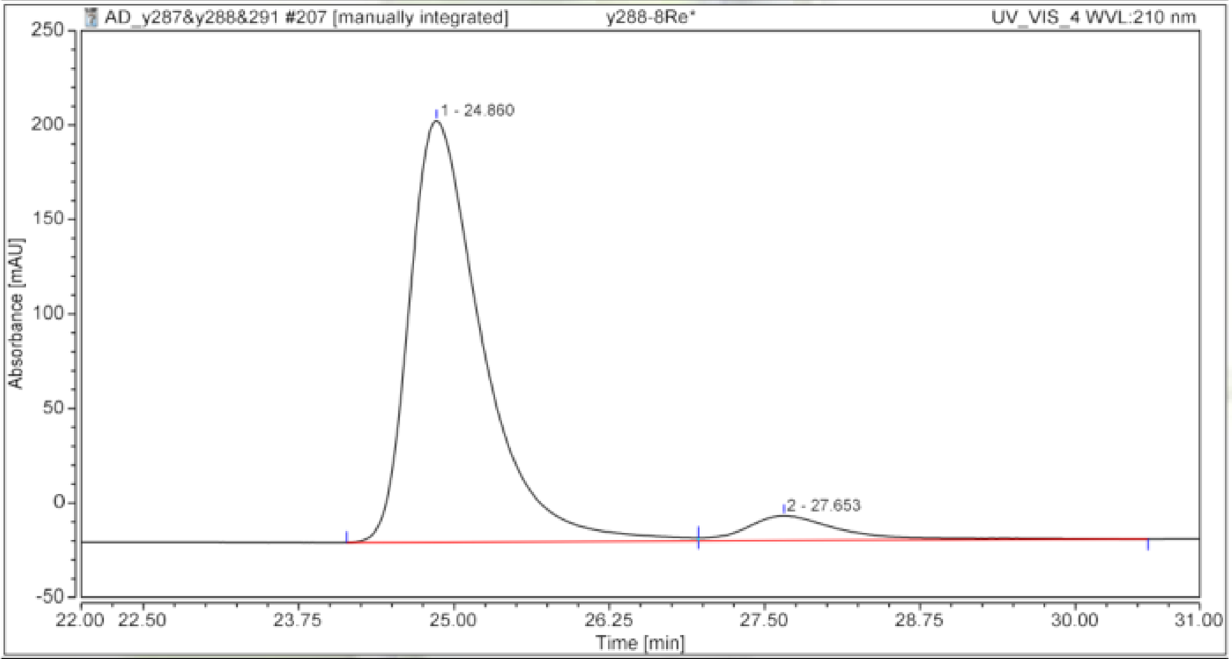

Integration Results

No.	Peak Name	Retention Time min	Area mAU*min	Height mAU	Relative Area %	Relative Height %	Amount n.a.
1		24.860	150.947	222.890	92.74	94.46	n.a.
2		27.653	11.824	13.066	7.26	5.54	n.a.
**Total:**			**162.770**	**235.956**	**100.00**	**100.00**	

THF:H_2O =1:6; %ee = 84%

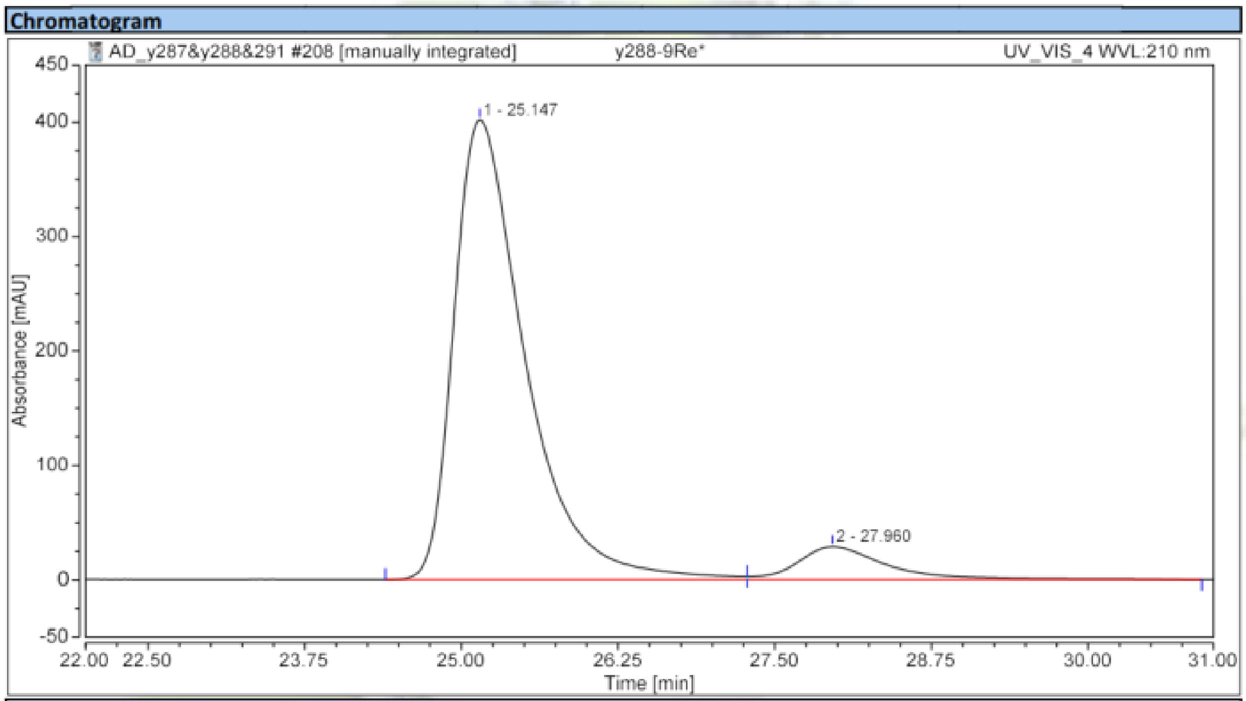

Integration Results

No.	Peak Name	Retention Time<br>min	Area<br>mAU*min	Height<br>mAU	Relative Area<br>%	Relative Height<br>%	Amount<br>n.a.
1		25.147	264.442	401.519	91.71	93.35	n.a.
2		27.960	23.900	28.616	8.29	6.65	n.a.
**Total:**			**288.342**	**430.136**	**100.00**	**100.00**	

(R)-1-(4-chlorophenyl)ethane-1,2-diol

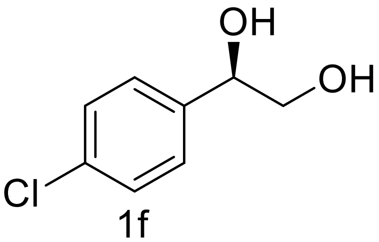

1f

- Column: Chiralpak OD-H.
- Condition: 3% *i*PrOH/Hexane at 0.5 mL/min, enantiomeric excess determined at 210 nm; 34.6 min (R), 40.2 min (S).

THF:H_2O =4.5:1; %ee = 80%

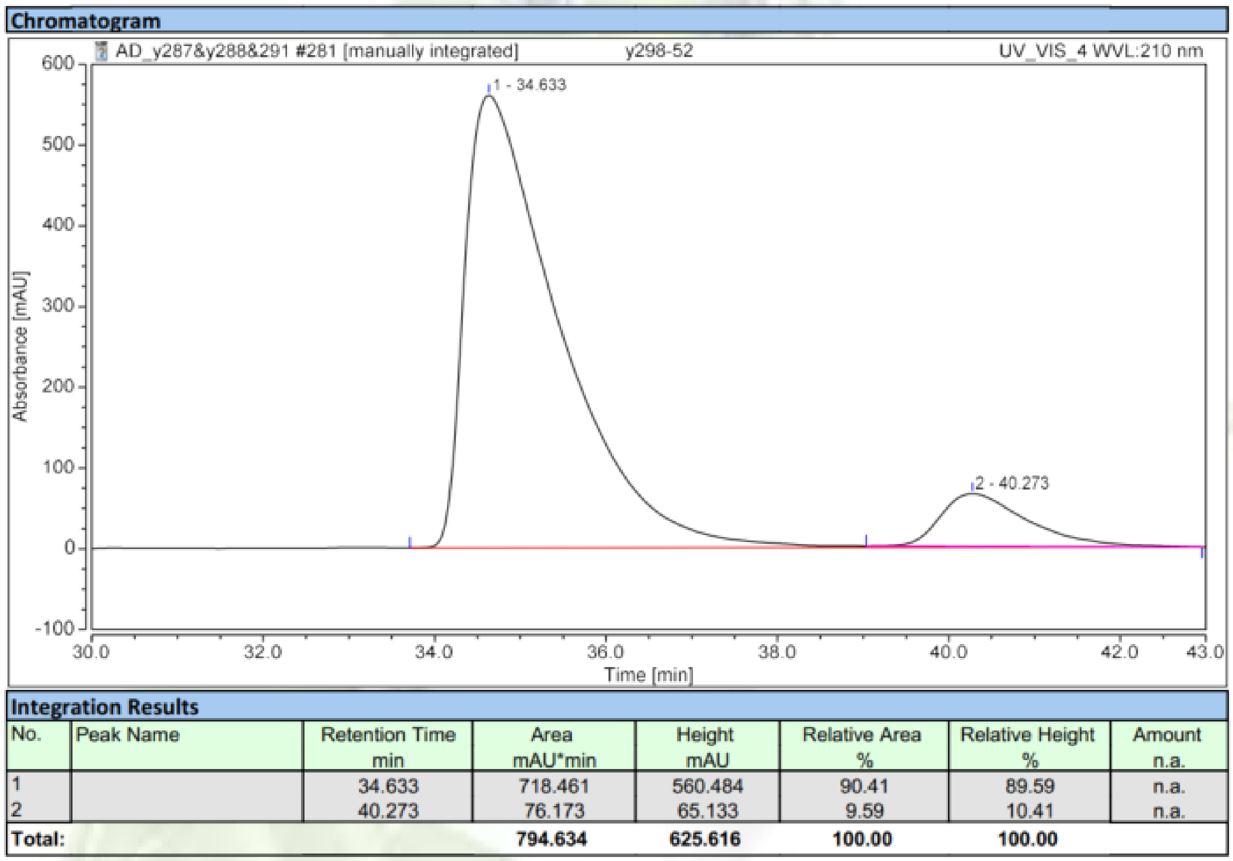

Integration Results

No.	Peak Name	Retention Time min	Area mAU*min	Height mAU	Relative Area %	Relative Height %	Amount n.a.
1		34.633	718.461	560.484	90.41	89.59	n.a.
2		40.273	76.173	65.133	9.59	10.41	n.a.
**Total:**			**794.634**	**625.616**	**100.00**	**100.00**	

THF:H_2O =3:1; %ee = 82%

Chromatogram

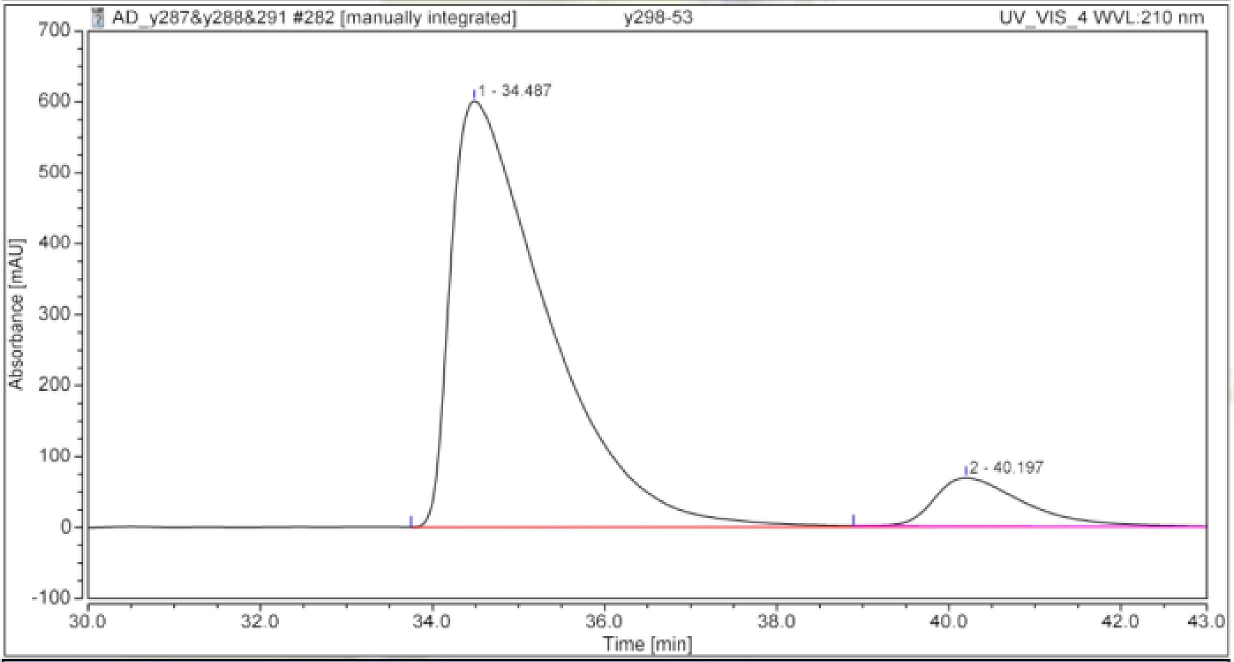

Integration Results

No.	Peak Name	Retention Time min	Area mAU*min	Height mAU	Relative Area %	Relative Height %	Amount n.a.
1		34.487	785.201	600.673	90.60	89.84	n.a.
2		40.197	81.460	67.922	9.40	10.16	n.a.
Total:			866.661	668.595	100.00	100.00	

THF:H_2O =1.5:1; %ee = 80%

Chromatogram

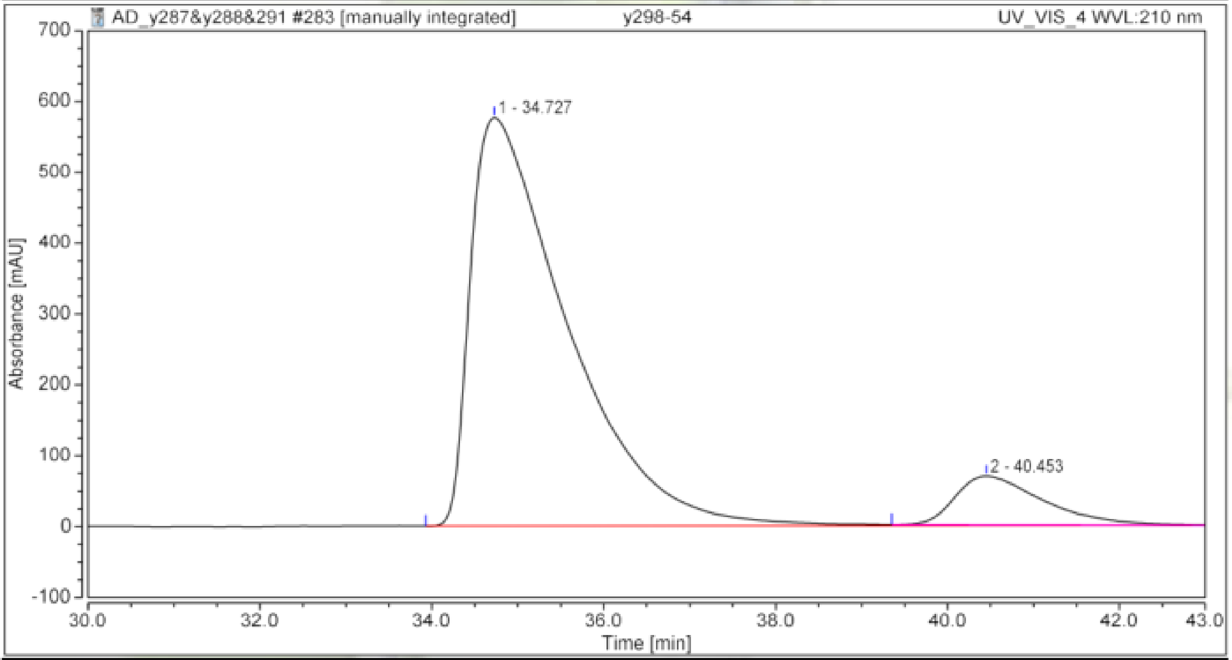

Integration Results

No.	Peak Name	Retention Time min	Area mAU*min	Height mAU	Relative Area %	Relative Height %	Amount n.a.
1		34.727	752.392	576.027	90.26	89.38	n.a.
2		40.453	81.208	68.412	9.74	10.62	n.a.
Total:			833.600	644.438	100.00	100.00	

THF:H_2O =1:1; %ee = 86%

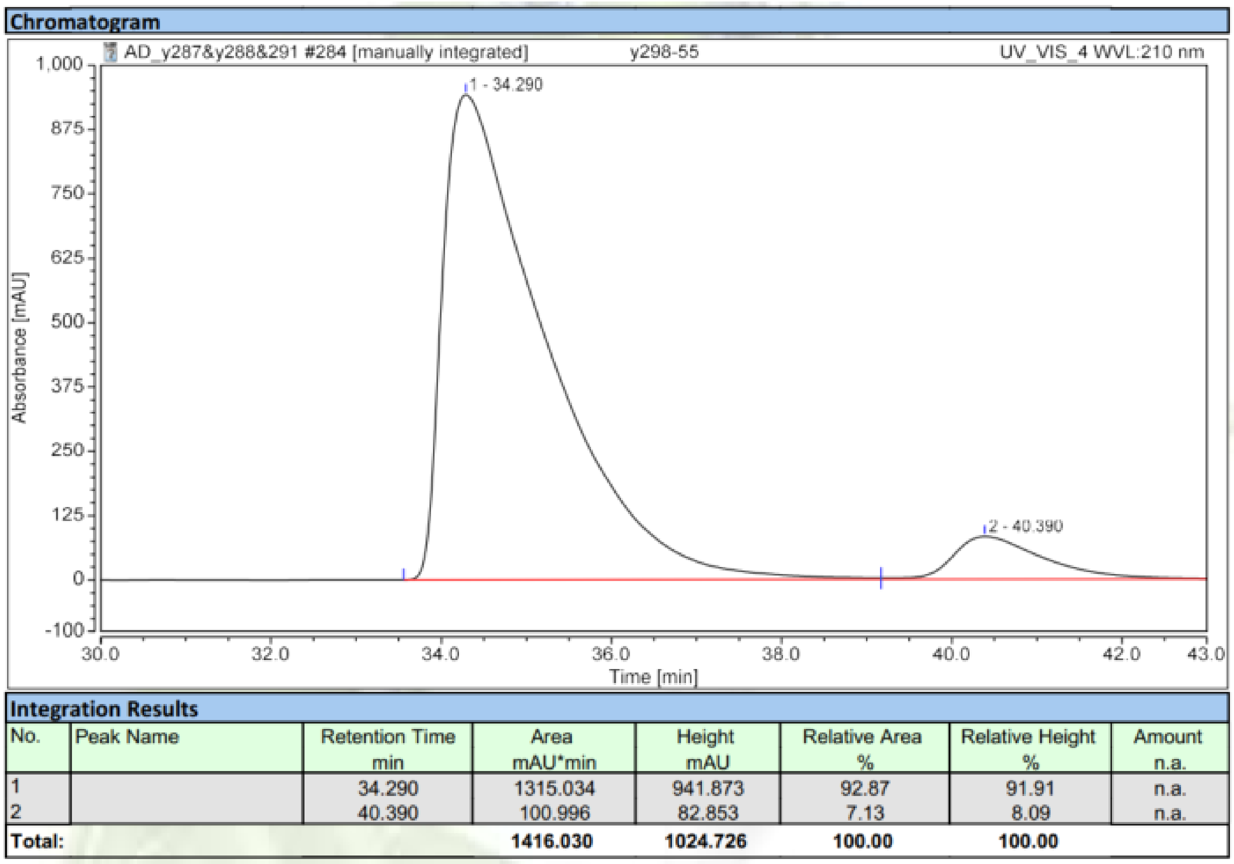

Integration Results

No.	Peak Name	Retention Time min	Area mAU*min	Height mAU	Relative Area %	Relative Height %	Amount n.a.
1		34.290	1315.034	941.873	92.87	91.91	n.a.
2		40.390	100.996	82.853	7.13	8.09	n.a.
**Total:**			**1416.030**	**1024.726**	**100.00**	**100.00**	

THF:H_2O =1:1.5; %ee = 86%

Chromatogram

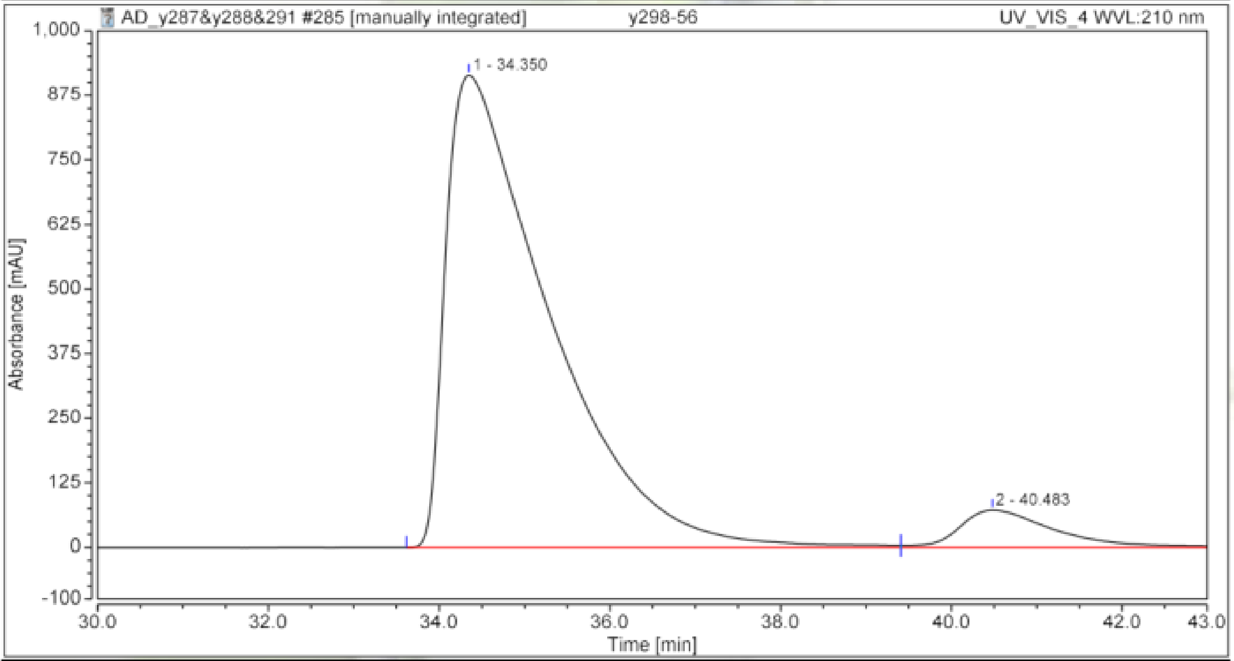

Integration Results

No.	Peak Name	Retention Time min	Area mAU*min	Height mAU	Relative Area %	Relative Height %	Amount n.a.
1		34.350	1281.247	914.086	93.15	92.68	n.a.
2		40.483	94.229	72.189	6.85	7.32	n.a.
**Total:**			**1375.476**	**986.275**	**100.00**	**100.00**	

THF:H_2O =1:3; %ee = 90%

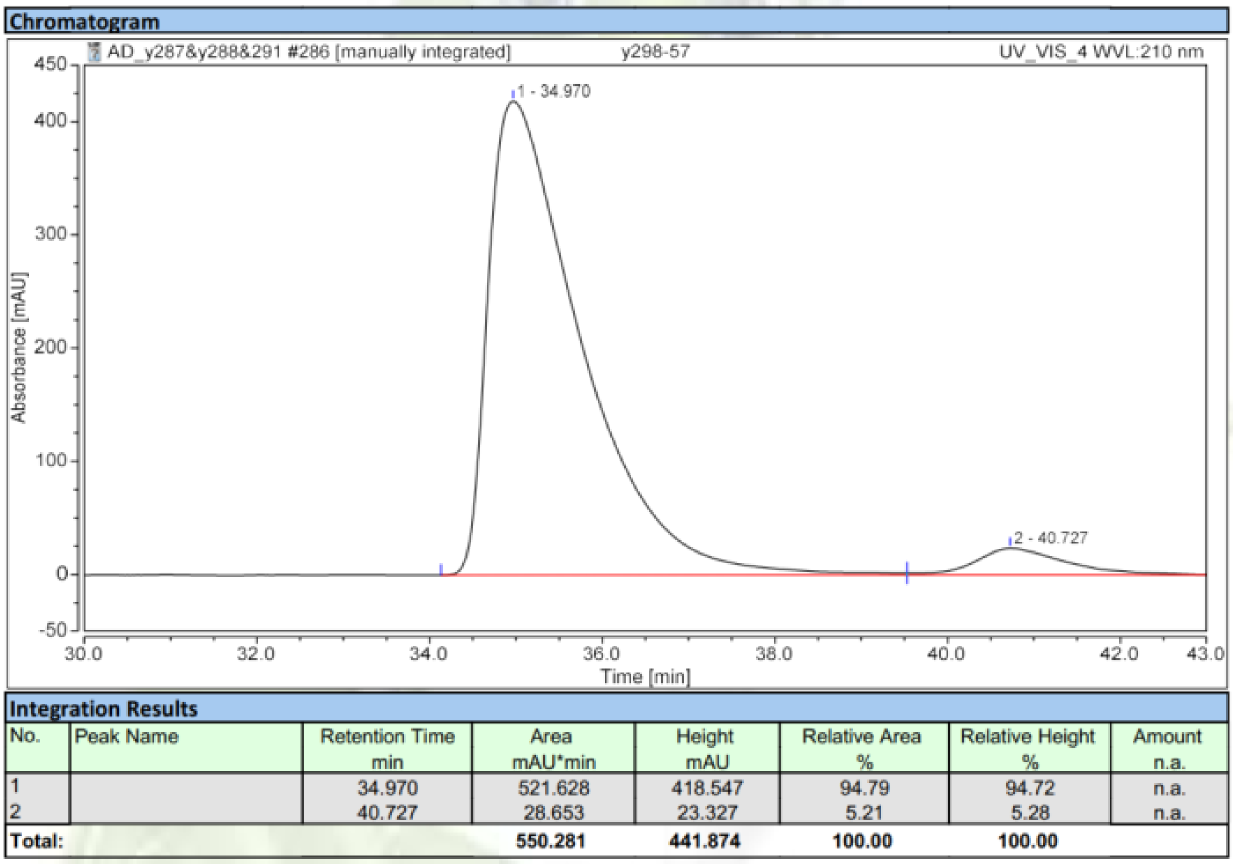

Integration Results

No.	Peak Name	Retention Time min	Area mAU*min	Height mAU	Relative Area %	Relative Height %	Amount n.a.
1		34.970	521.628	418.547	94.79	94.72	n.a.
2		40.727	28.653	23.327	5.21	5.28	n.a.
**Total:**			**550.281**	**441.874**	**100.00**	**100.00**	

THF:H_2O =1:4.5; %ee = 90%

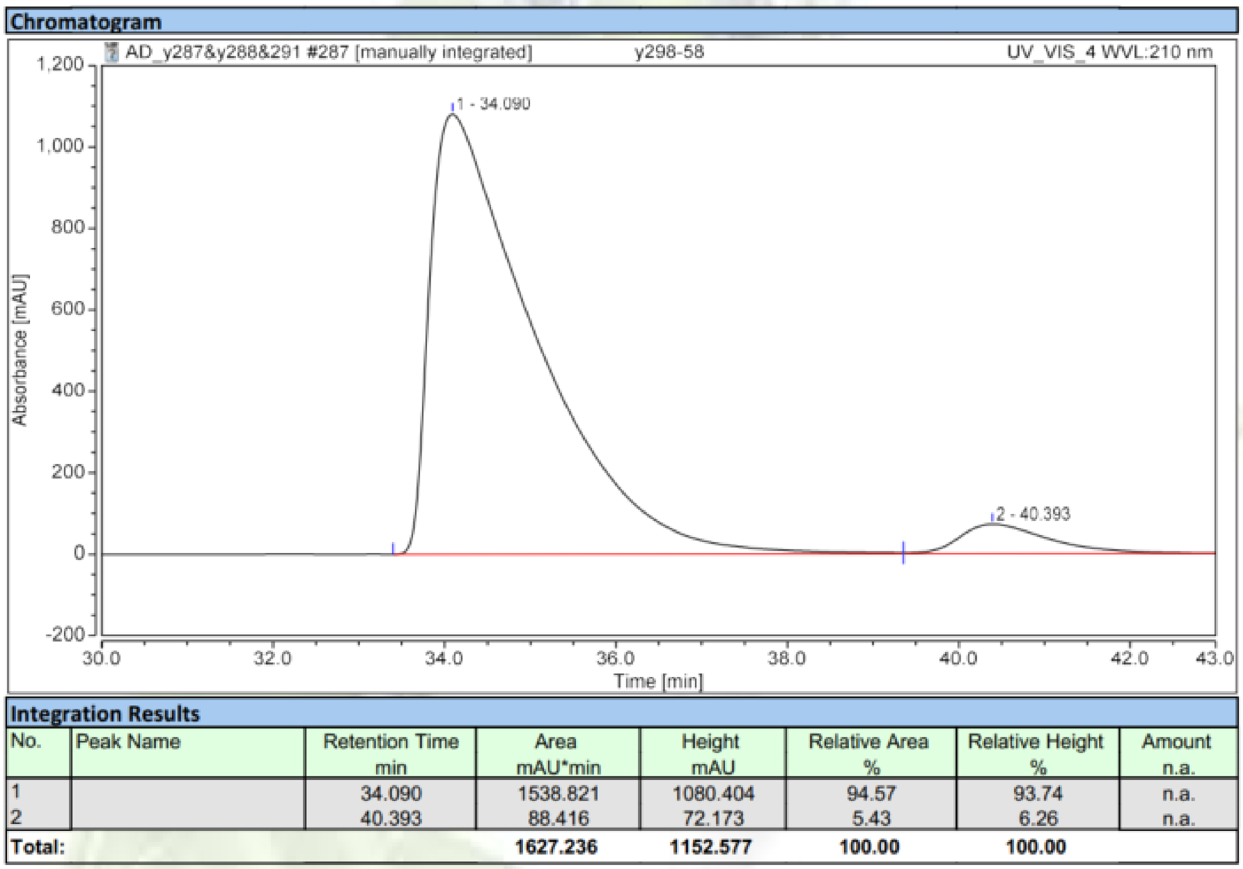

Integration Results

No.	Peak Name	Retention Time min	Area mAU*min	Height mAU	Relative Area %	Relative Height %	Amount n.a.
1		34.090	1538.821	1080.404	94.57	93.74	n.a.
2		40.393	88.416	72.173	5.43	6.26	n.a.
**Total:**			**1627.236**	**1152.577**	**100.00**	**100.00**	

THF:H_2O =1:6; %ee = 86%

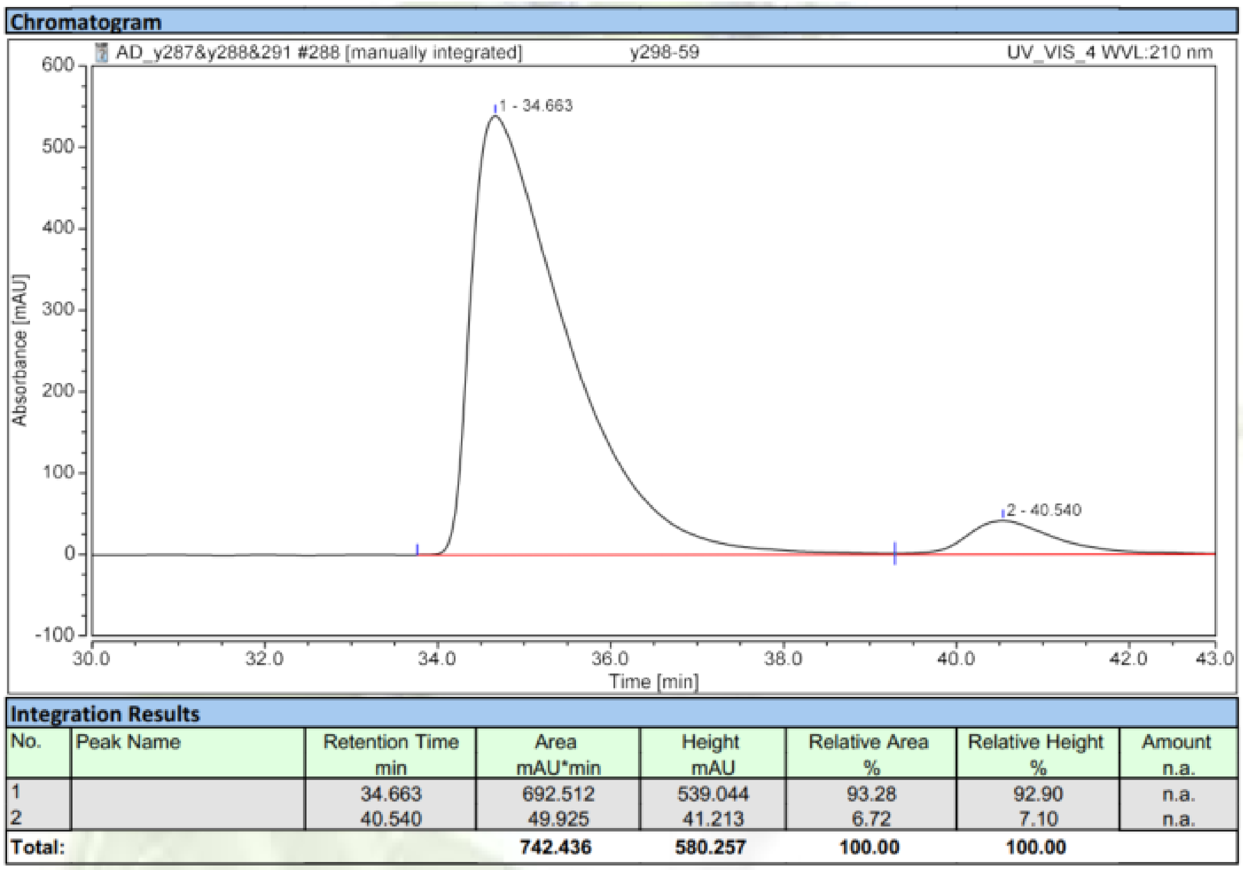

Integration Results

No.	Peak Name	Retention Time min	Area mAU*min	Height mAU	Relative Area %	Relative Height %	Amount n.a.
1		34.663	692.512	539.044	93.28	92.90	n.a.
2		40.540	49.925	41.213	6.72	7.10	n.a.
**Total:**			**742.436**	**580.257**	**100.00**	**100.00**	

(S)-1-phenylethane-1,2-diol

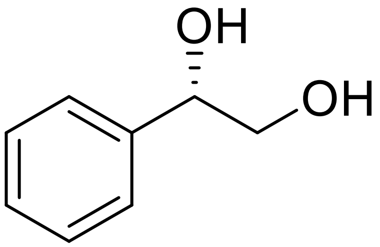

2a

- Column: Chiralpak OD-H.
- Condition: 10% *i*PrOH/Hexane at 0.5 mL/min, enantiomeric excess determined at 210 nm; 15.6 min (R), 16.9 min (S).

THF:H_2O =4.5:1; %ee = 64%

Chromatogram

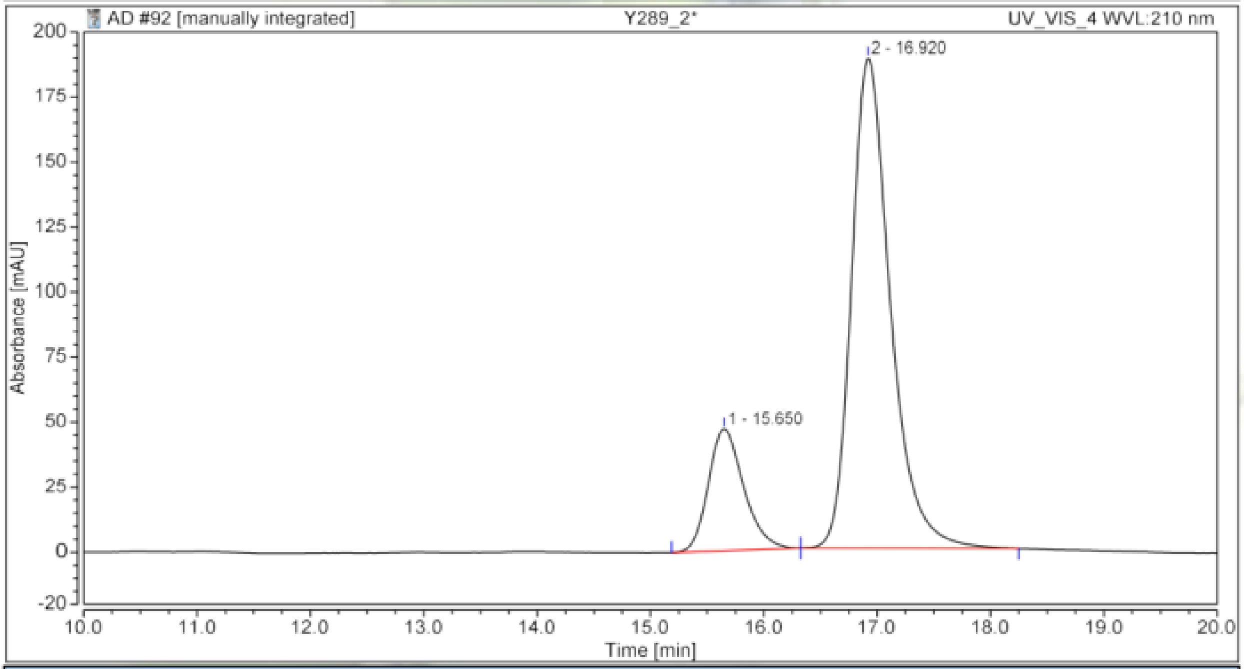

Integration Results

No.	Peak Name	Retention Time min	Area mAU*min	Height mAU	Relative Area %	Relative Height %	Amount n.a.
1		15.650	16.896	46.812	18.39	19.90	n.a.
2		16.920	74.956	188.397	81.61	80.10	n.a.
**Total:**			**91.852**	**235.208**	**100.00**	**100.00**	

THF:H_2O =3:1; %ee = 64%

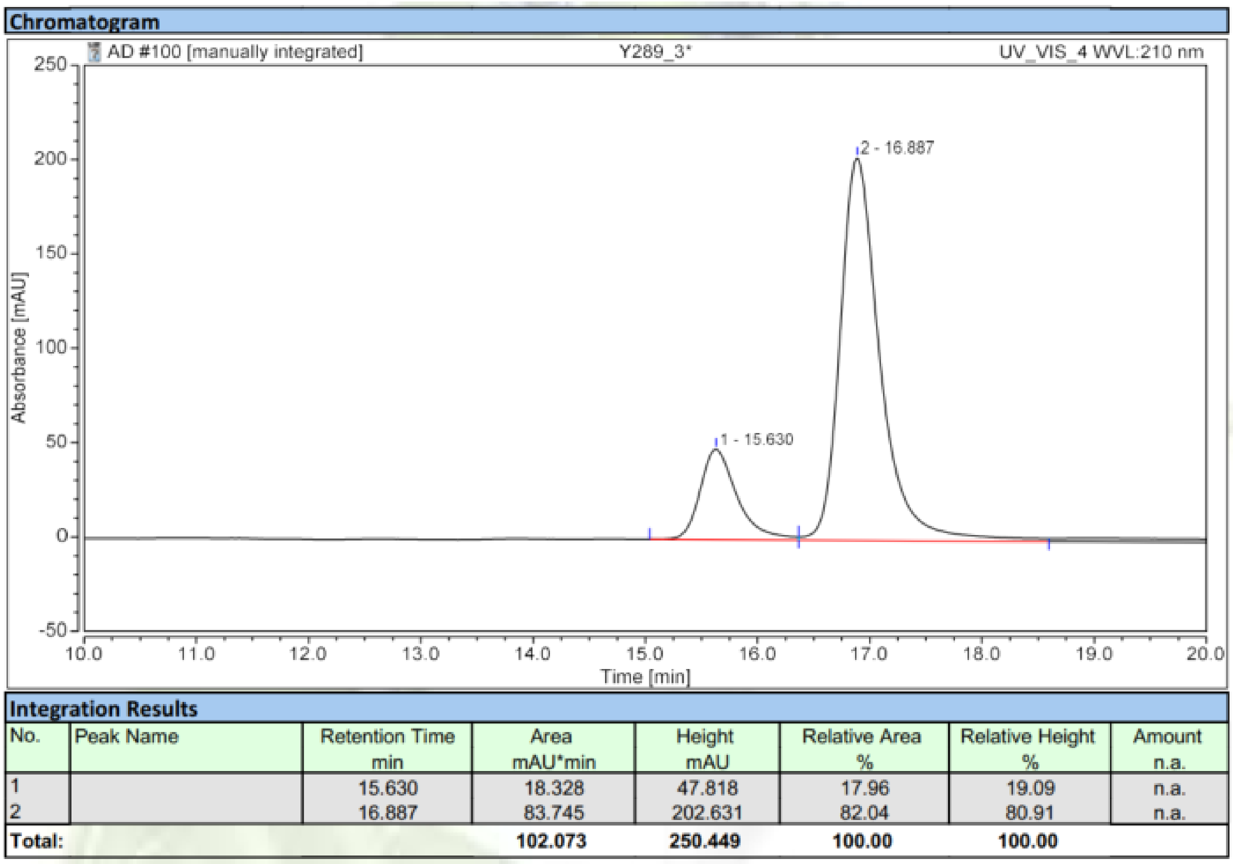

Integration Results

No.	Peak Name	Retention Time min	Area mAU*min	Height mAU	Relative Area %	Relative Height %	Amount n.a.
1		15.630	18.328	47.818	17.96	19.09	n.a.
2		16.887	83.745	202.631	82.04	80.91	n.a.
**Total:**			**102.073**	**250.449**	**100.00**	**100.00**	

THF:H_2O =1.5:1; %ee = 70%

Chromatogram

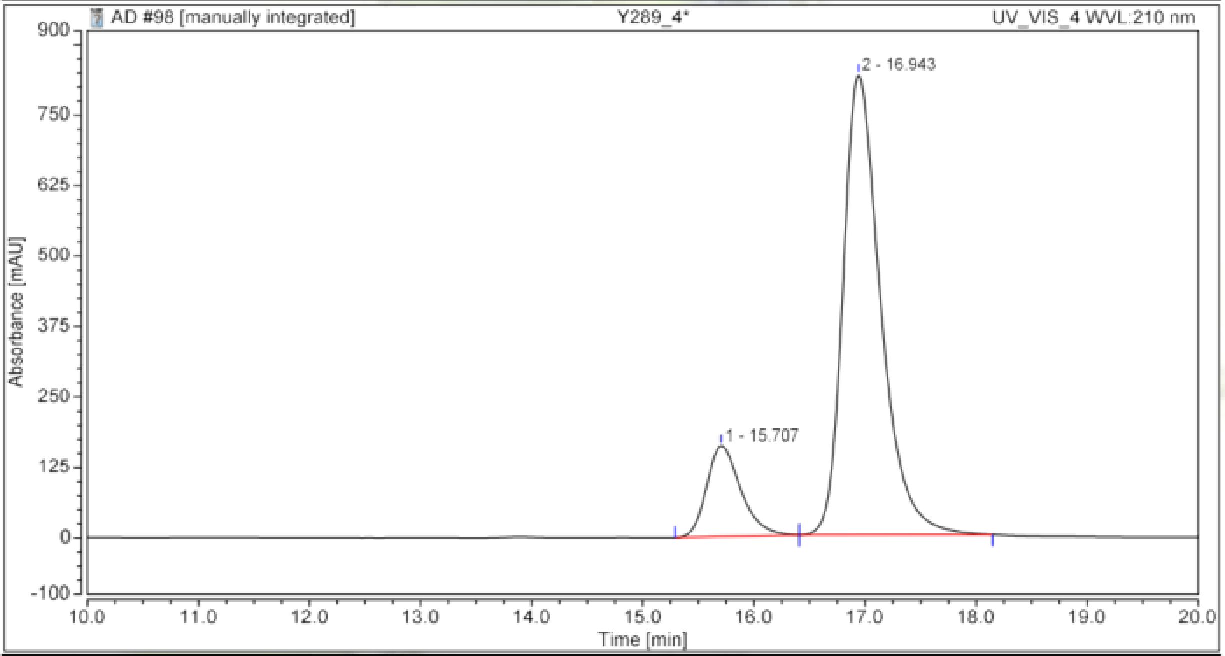

Integration Results

No.	Peak Name	Retention Time min	Area mAU*min	Height mAU	Relative Area %	Relative Height %	Amount n.a.
1		15.707	57.490	161.108	15.18	16.49	n.a.
2		16.943	321.169	816.131	84.82	83.51	n.a.
**Total:**			**378.659**	**977.239**	**100.00**	**100.00**	

THF:H_2O =1:1; %ee = 74%

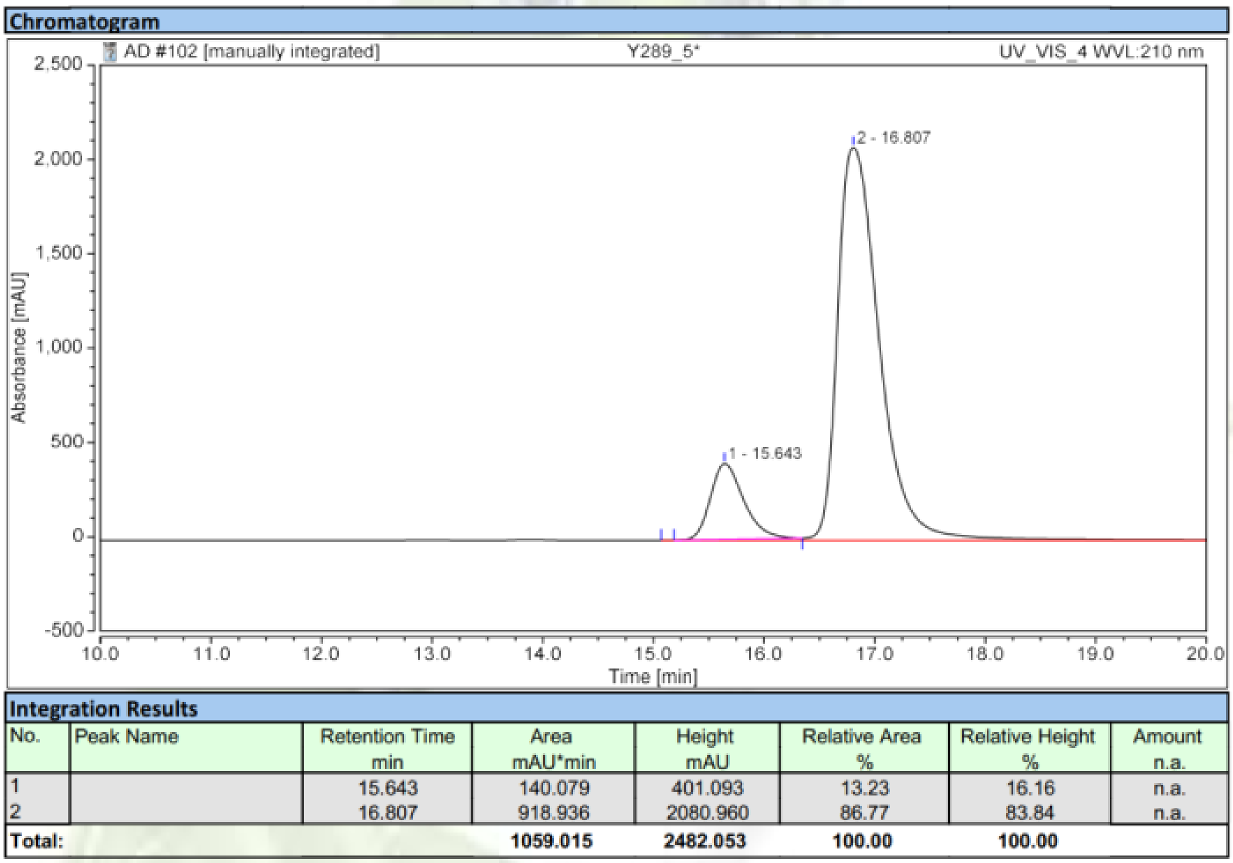

Integration Results

No.	Peak Name	Retention Time min	Area mAU*min	Height mAU	Relative Area %	Relative Height %	Amount n.a.
1		15.643	140.079	401.093	13.23	16.16	n.a.
2		16.807	918.936	2080.960	86.77	83.84	n.a.
**Total:**			**1059.015**	**2482.053**	**100.00**	**100.00**	

THF:H_2O =1:1.5; %ee = 80%

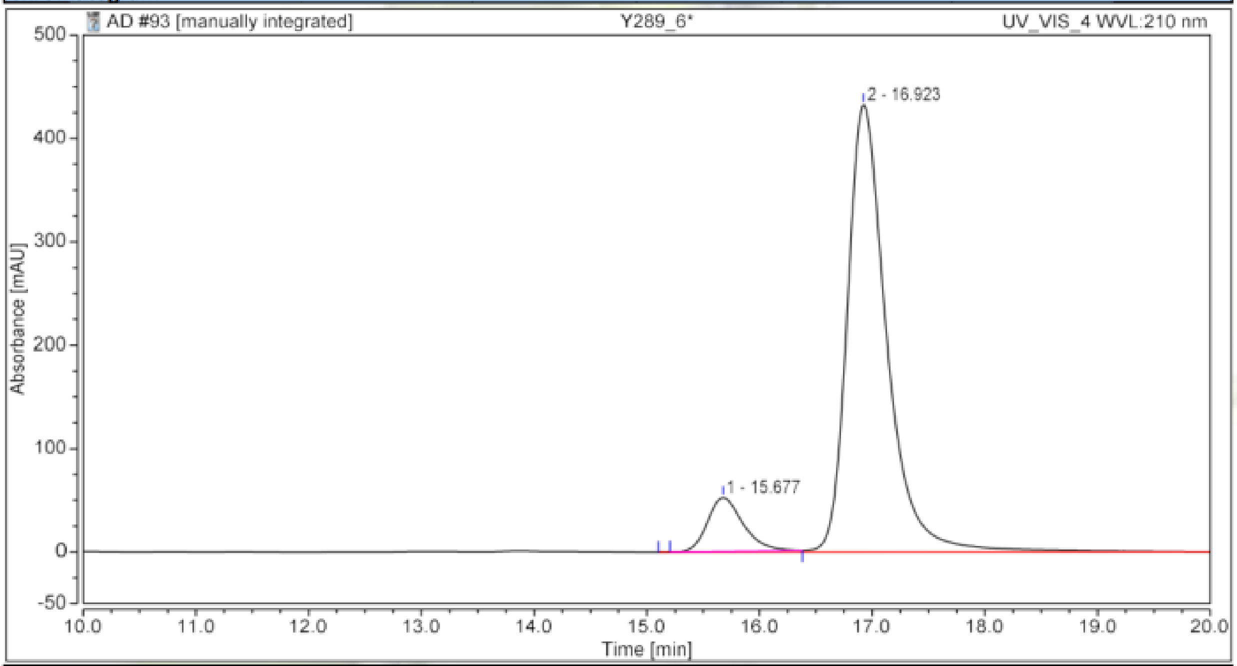

Integration Results

No.	Peak Name	Retention Time min	Area mAU*min	Height mAU	Relative Area %	Relative Height %	Amount n.a.
1		15.677	19.003	52.010	9.68	10.73	n.a.
2		16.923	177.355	432.846	90.32	89.27	n.a.
**Total:**			**196.357**	**484.856**	**100.00**	**100.00**	

THF:H_2O =1:3; %ee = 86%

Chromatogram

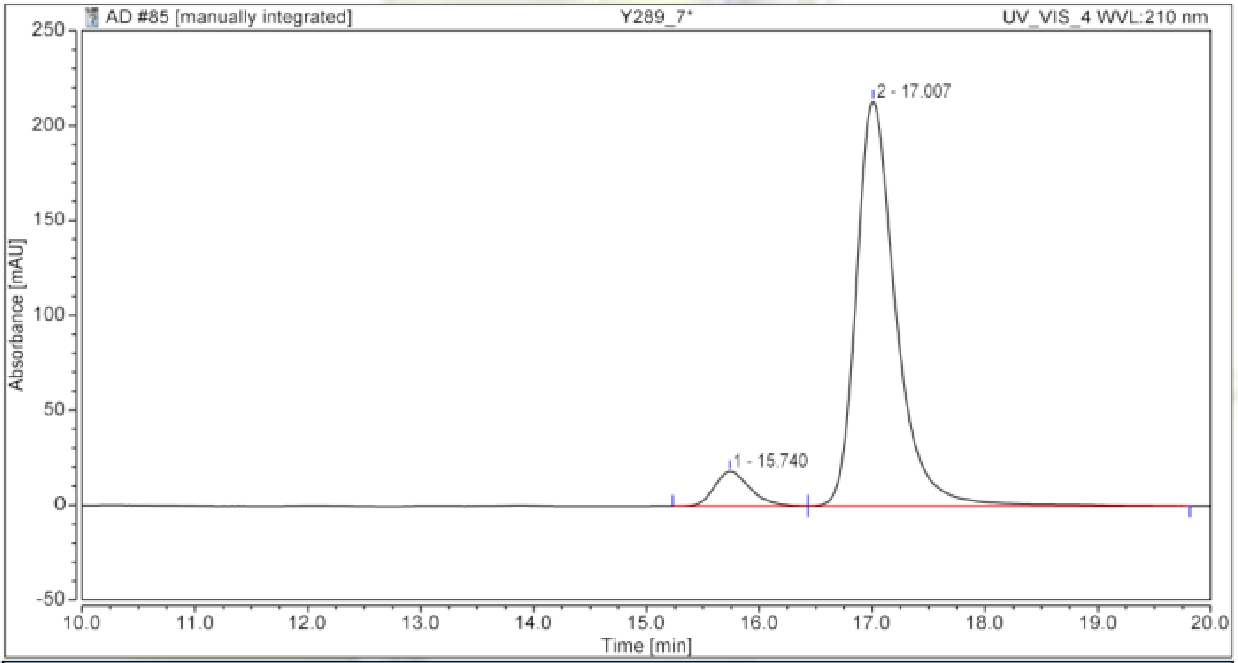

Integration Results

No.	Peak Name	Retention Time min	Area mAU*min	Height mAU	Relative Area %	Relative Height %	Amount n.a.
1		15.740	6.830	18.403	7.26	7.94	n.a.
2		17.007	87.280	213.409	92.74	92.06	n.a.
**Total:**			**94.110**	**231.812**	**100.00**	**100.00**	

THF:H_2O =1:4.5; %ee = 80%

Chromatogram

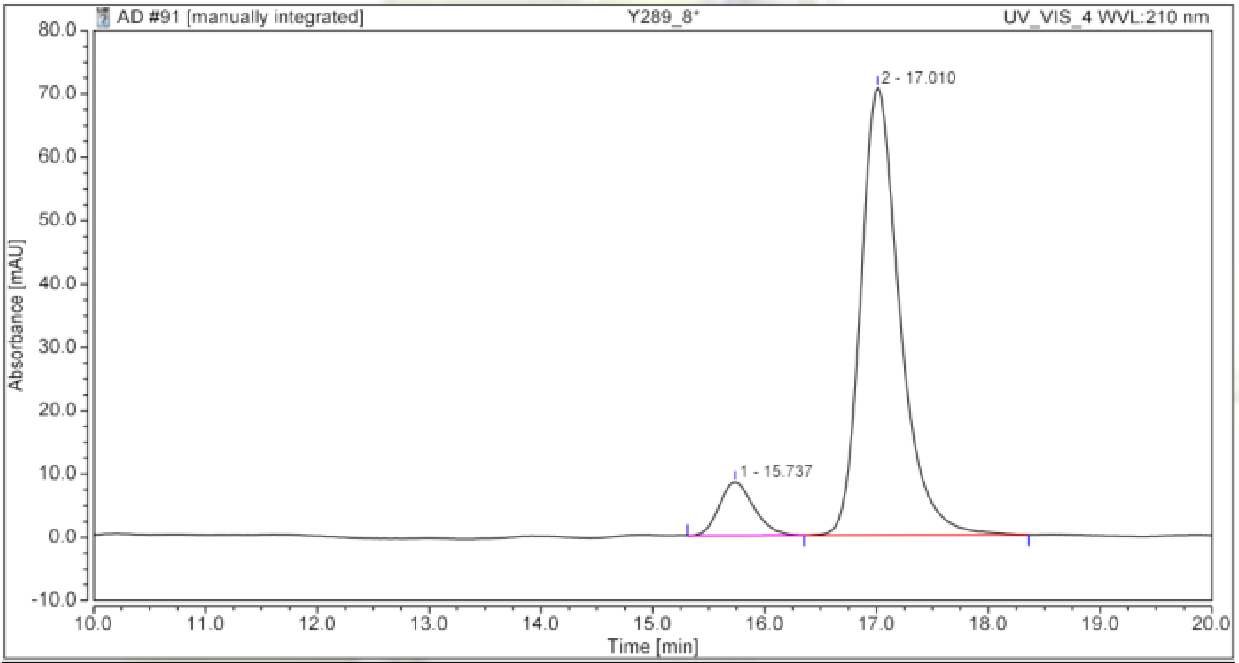

Integration Results

No.	Peak Name	Retention Time min	Area mAU*min	Height mAU	Relative Area %	Relative Height %	Amount n.a.
1		15.737	3.017	8.418	9.52	10.64	n.a.
2		17.010	28.680	70.666	90.48	89.36	n.a.
**Total:**			**31.697**	**79.085**	**100.00**	**100.00**	

THF:H_2O =1:6; %ee = 76%

Chromatogram

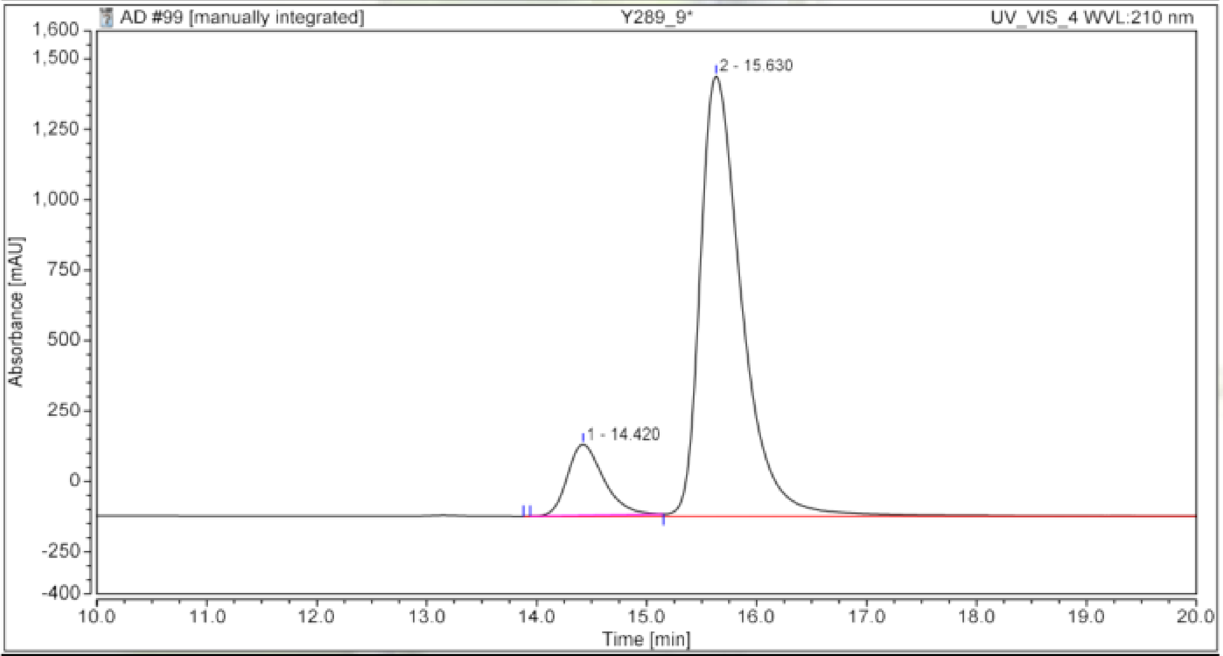

Integration Results

No.	Peak Name	Retention Time<br>min	Area<br>mAU*min	Height<br>mAU	Relative Area<br>%	Relative Height<br>%	Amount<br>n.a.
1		14.420	95.848	252.520	12.17	13.91	n.a.
2		15.630	691.641	1562.690	87.83	86.09	n.a.
**Total:**			**787.489**	**1815.210**	**100.00**	**100.00**	

(S)-1-(p-tolyl)ethane-1,2-diol

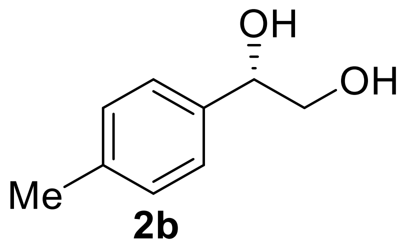

2b

- Column: Chiralpak OD-H.
- Condition: 10% *i*PrOH/Hexane at 0.5 mL/min, enantiomeric excess determined at 210 nm; 14.4 min (R), 15.8 min (S).

THF:H_2O =4.5:1; %ee = 68%

Chromatogram

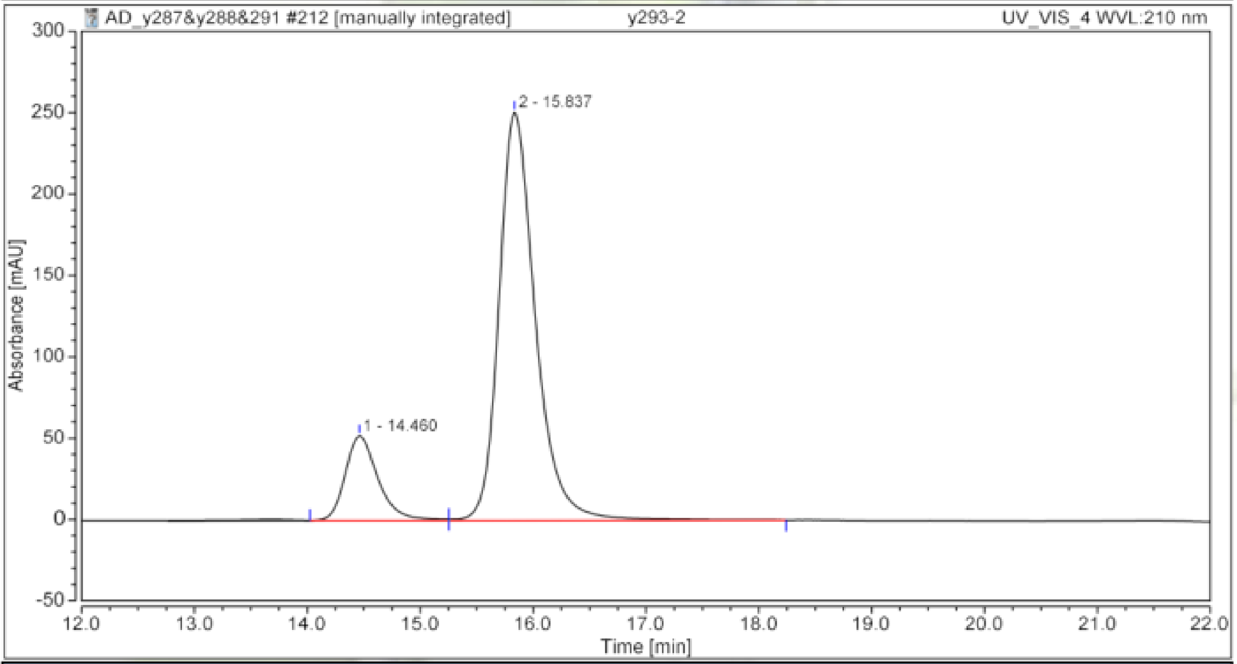

Integration Results

No.	Peak Name	Retention Time min	Area mAU*min	Height mAU	Relative Area %	Relative Height %	Amount n.a.
1		14.460	17.447	51.883	15.72	17.15	n.a.
2		15.837	93.520	250.613	84.28	82.85	n.a.
Total:			110.968	302.496	100.00	100.00	

THF:H_2O =3:1; %ee = 70%

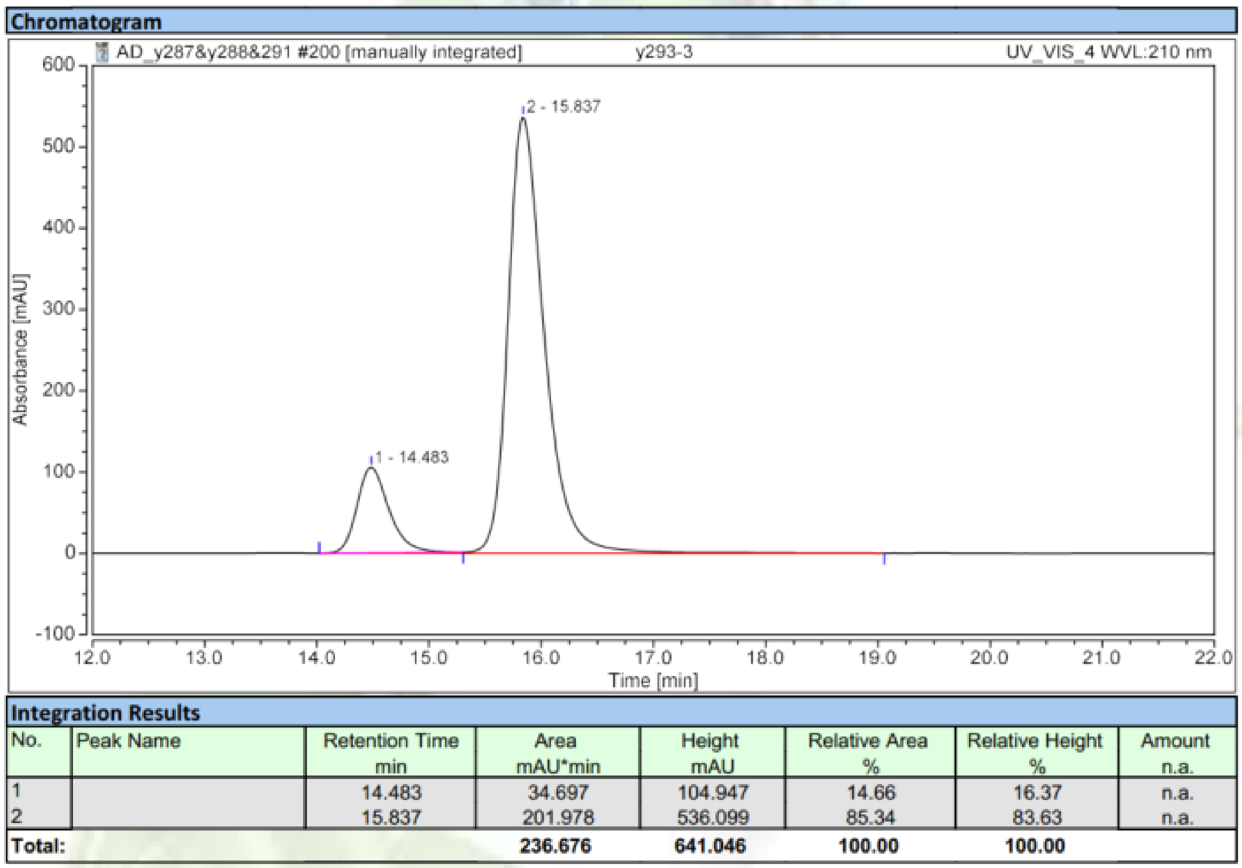

Integration Results

No.	Peak Name	Retention Time min	Area mAU*min	Height mAU	Relative Area %	Relative Height %	Amount n.a.
1		14.483	34.697	104.947	14.66	16.37	n.a.
2		15.837	201.978	536.099	85.34	83.63	n.a.
**Total:**			**236.676**	**641.046**	**100.00**	**100.00**	

THF:H_2O =1.5:1; %ee = 74%

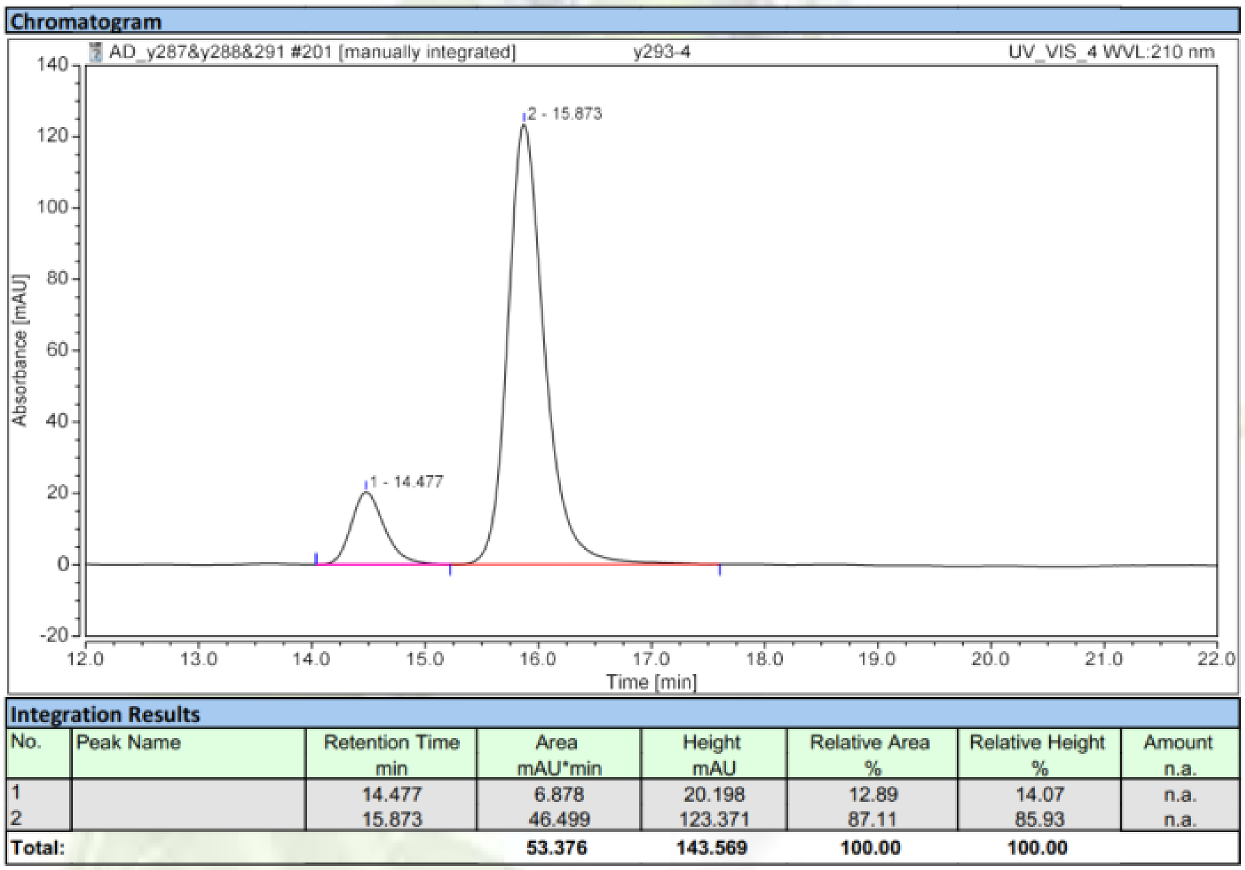

Integration Results

No.	Peak Name	Retention Time min	Area mAU*min	Height mAU	Relative Area %	Relative Height %	Amount n.a.
1		14.477	6.878	20.198	12.89	14.07	n.a.
2		15.873	46.499	123.371	87.11	85.93	n.a.
Total:			53.376	143.569	100.00	100.00	

THF:H_2O =1:1; %ee = 80%

Chromatogram

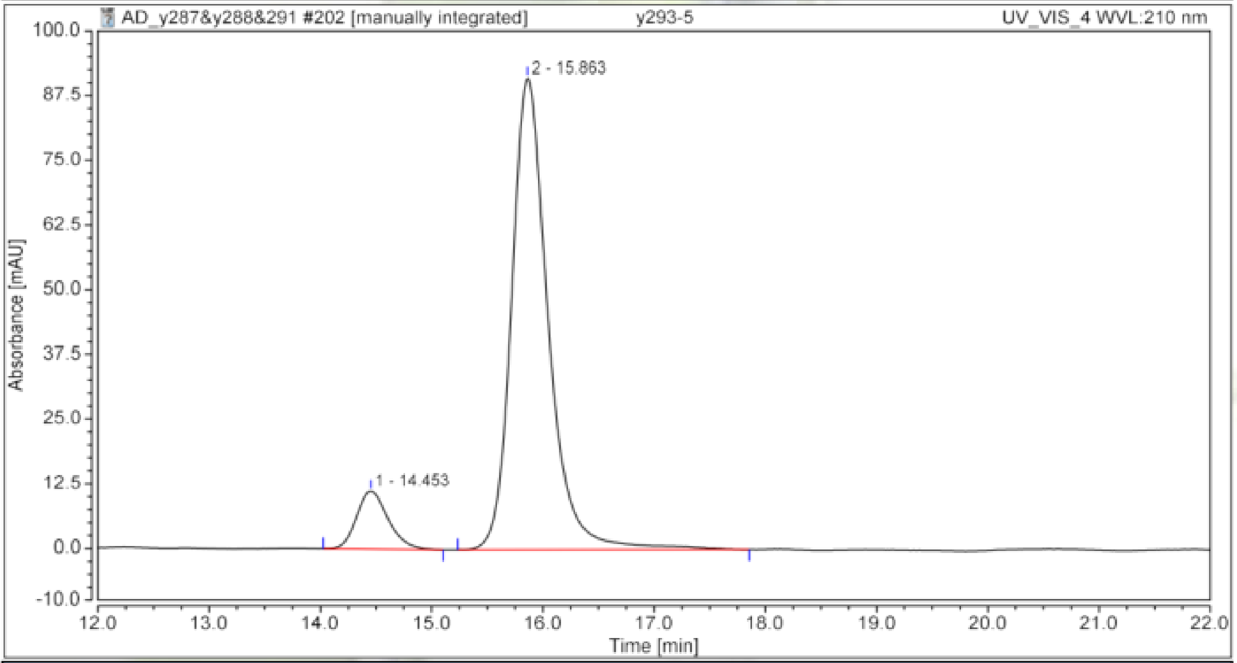

Integration Results

No.	Peak Name	Retention Time min	Area mAU*min	Height mAU	Relative Area %	Relative Height %	Amount n.a.
1		14.453	3.694	11.213	9.58	10.95	n.a.
2		15.863	34.854	91.161	90.42	89.05	n.a.
**Total:**			**38.549**	**102.375**	**100.00**	**100.00**	

THF:H_2O =1:1.5; %ee = 82%

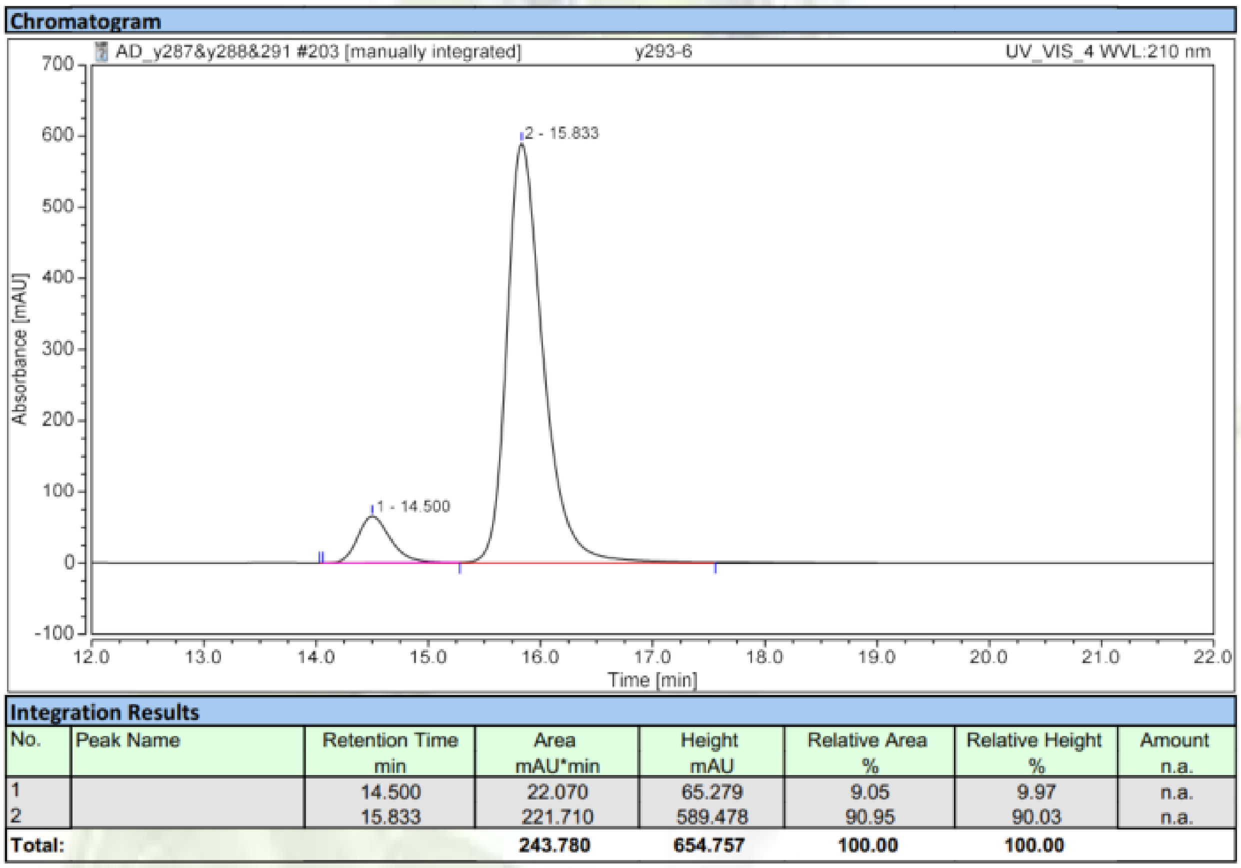

Integration Results

No.	Peak Name	Retention Time<br>min	Area<br>mAU*min	Height<br>mAU	Relative Area<br>%	Relative Height<br>%	Amount<br>n.a.
1		14.500	22.070	65.279	9.05	9.97	n.a.
2		15.833	221.710	589.478	90.95	90.03	n.a.
**Total:**			**243.780**	**654.757**	**100.00**	**100.00**	

THF:H_2O =1:3; %ee = 86%

Chromatogram

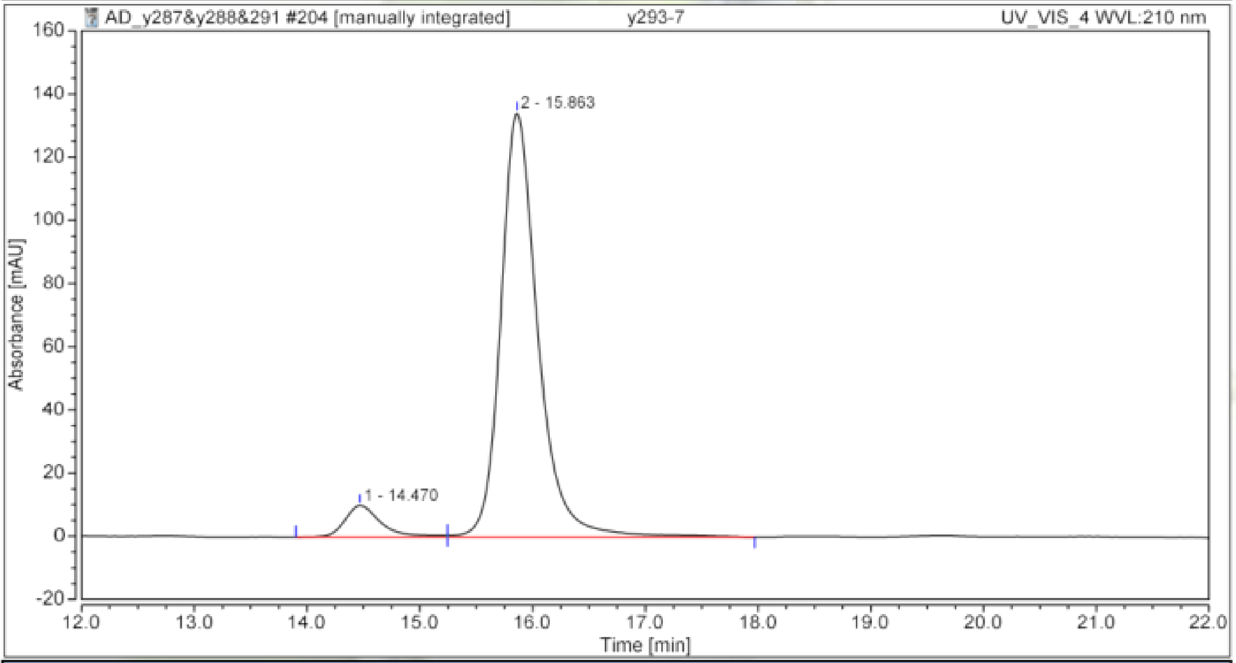

Integration Results

No.	Peak Name	Retention Time min	Area mAU*min	Height mAU	Relative Area %	Relative Height %	Amount n.a.
1		14.470	3.701	9.953	6.72	6.90	n.a.
2		15.863	51.362	134.196	93.28	93.10	n.a.
**Total:**			**55.064**	**144.149**	**100.00**	**100.00**	

THF:H_2O =1:4.5; %ee = 86%

Chromatogram

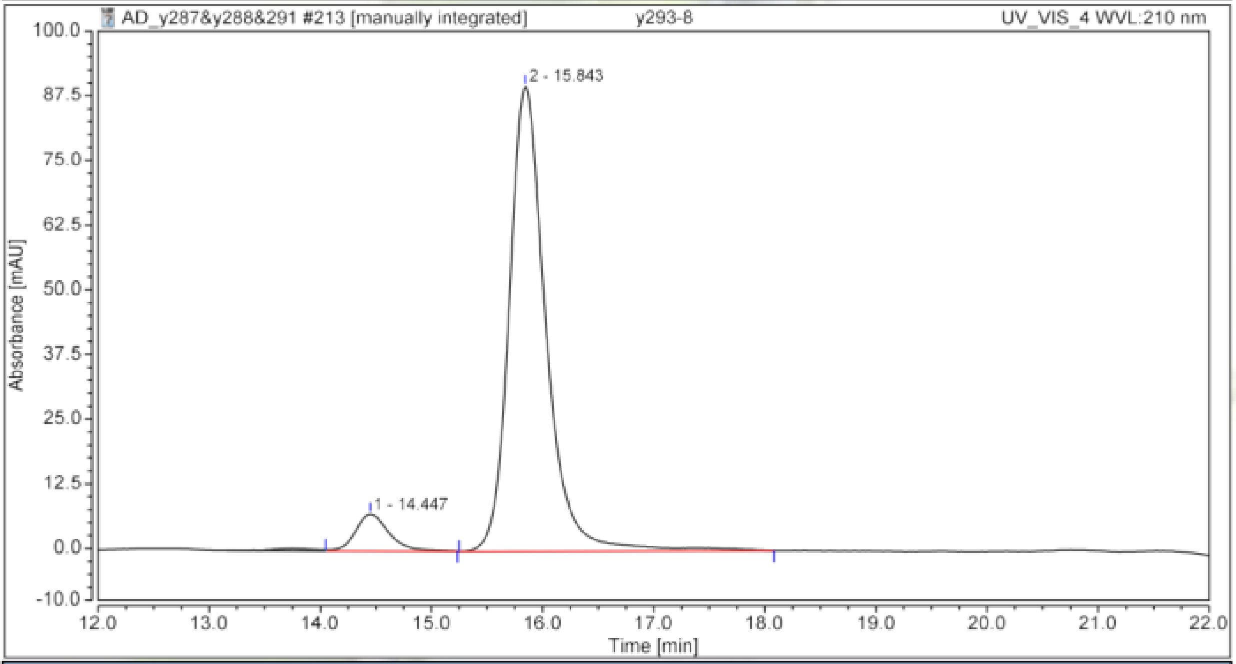

Integration Results

No.	Peak Name	Retention Time min	Area mAU*min	Height mAU	Relative Area %	Relative Height %	Amount n.a.
1		14.447	2.481	7.106	6.71	7.33	n.a.
2		15.843	34.514	89.879	93.29	92.67	n.a.
Total:			36.995	96.986	100.00	100.00	

THF:H_2O =1:6; %ee = 86%

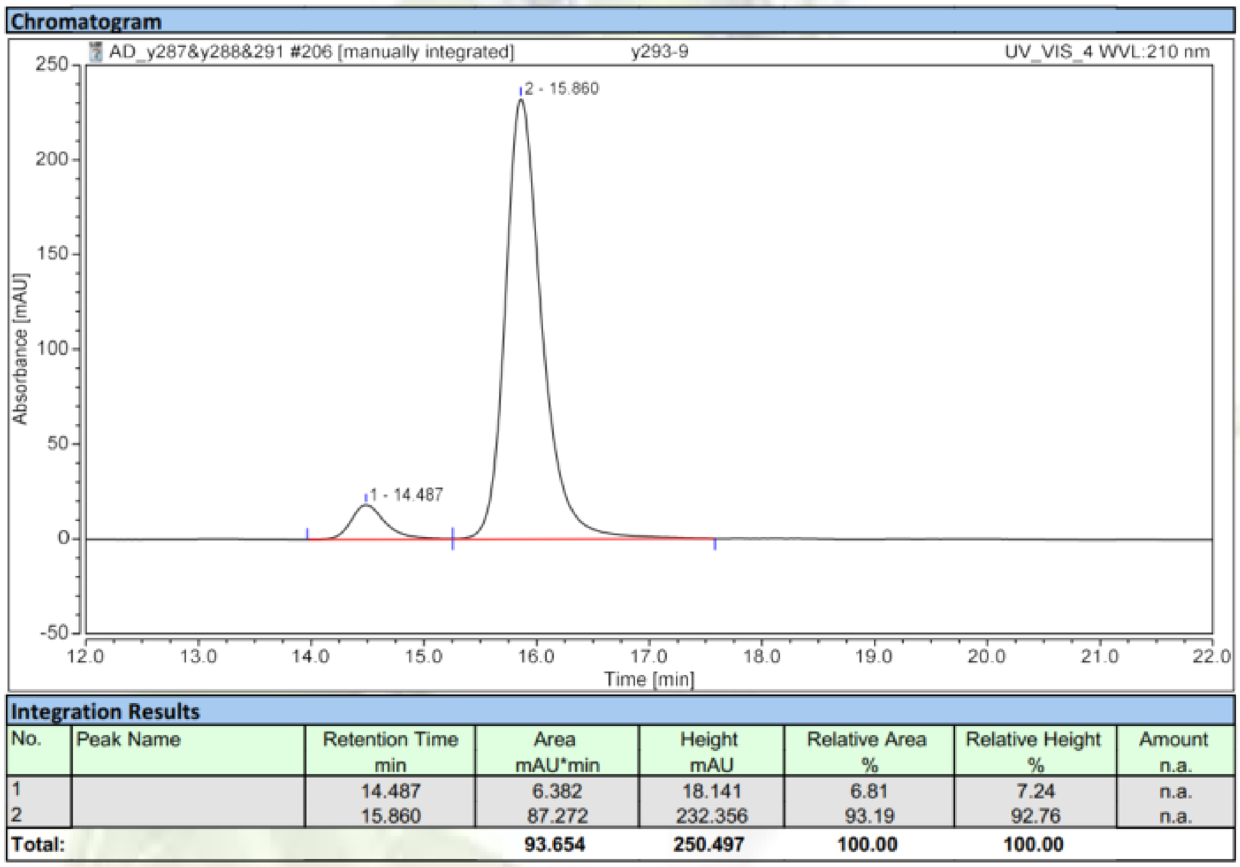

Integration Results

No.	Peak Name	Retention Time min	Area mAU*min	Height mAU	Relative Area %	Relative Height %	Amount n.a.
1		14.487	6.382	18.141	6.81	7.24	n.a.
2		15.860	87.272	232.356	93.19	92.76	n.a.
**Total:**			**93.654**	**250.497**	**100.00**	**100.00**	

(1R,2R)-1-phenylpropane-1,2-diol

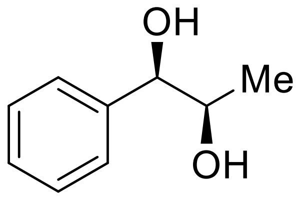

3a

- Column: Chiralpak OJ-H.
- Condition: 5% *i*PrOH/Hexane at 0.5 mL/min, enantiomeric excess determined at 210 nm; 32.3 min (SS), 34.6 min (RR).

THF:H_2O =4.5:1; %ee = 78%

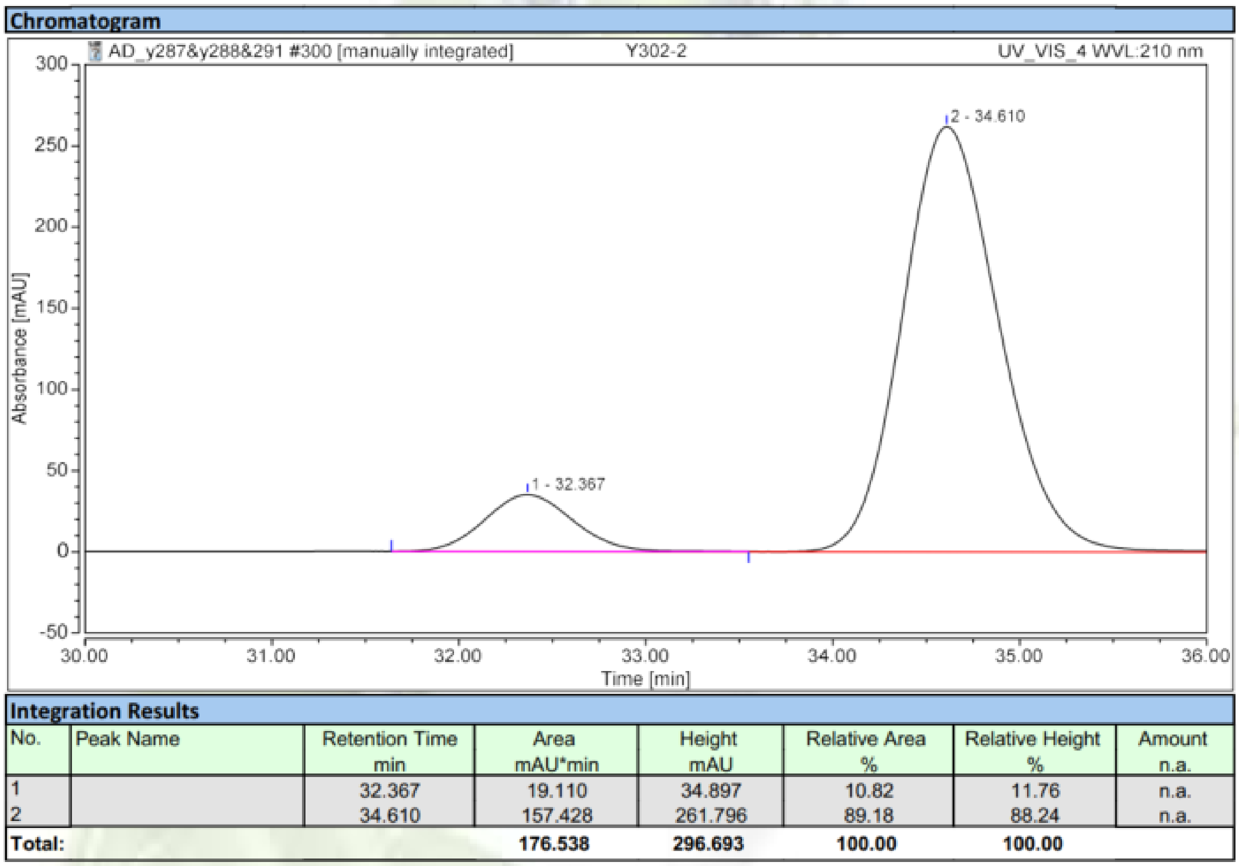

Integration Results

No.	Peak Name	Retention Time min	Area mAU*min	Height mAU	Relative Area %	Relative Height %	Amount n.a.
1		32.367	19.110	34.897	10.82	11.76	n.a.
2		34.610	157.428	261.796	89.18	88.24	n.a.
**Total:**			**176.538**	**296.693**	**100.00**	**100.00**	

THF:H_2O =3:1; %ee = 78%

Chromatogram

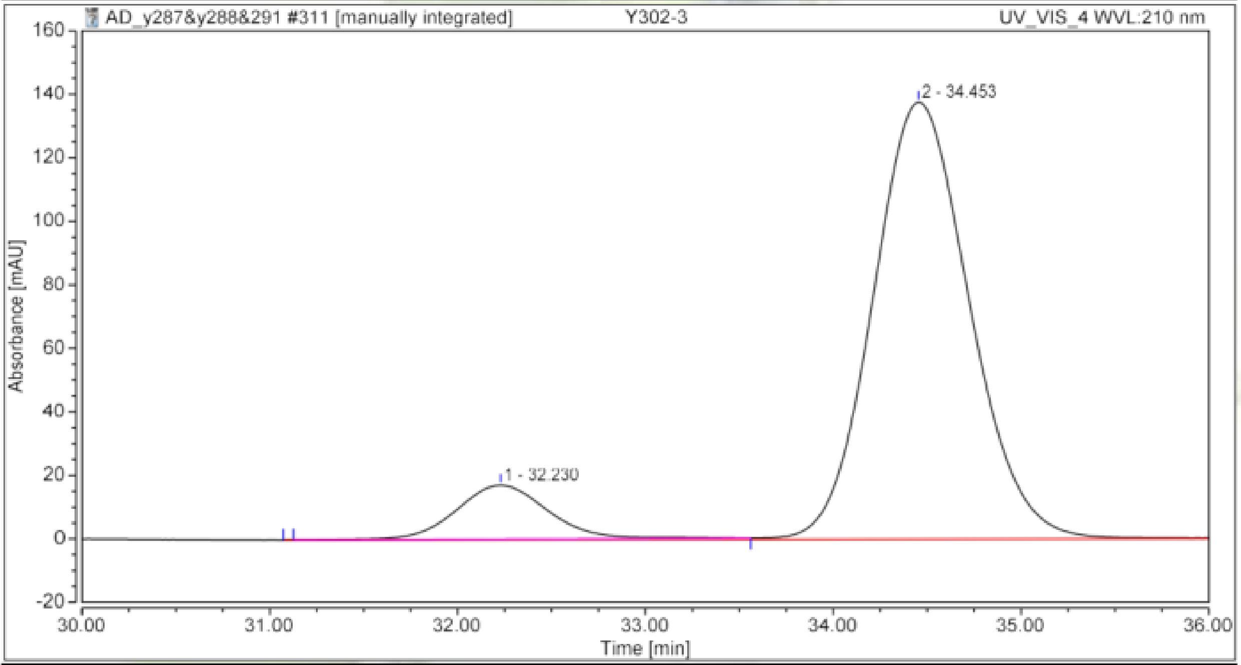

Integration Results

No.	Peak Name	Retention Time min	Area mAU*min	Height mAU	Relative Area %	Relative Height %	Amount n.a.
1		32.230	9.647	17.017	10.51	11.01	n.a.
2		34.453	82.105	137.582	89.49	88.99	n.a.
**Total:**			**91.752**	**154.599**	**100.00**	**100.00**	

THF:H_2O =1.5:1; %ee = 82%

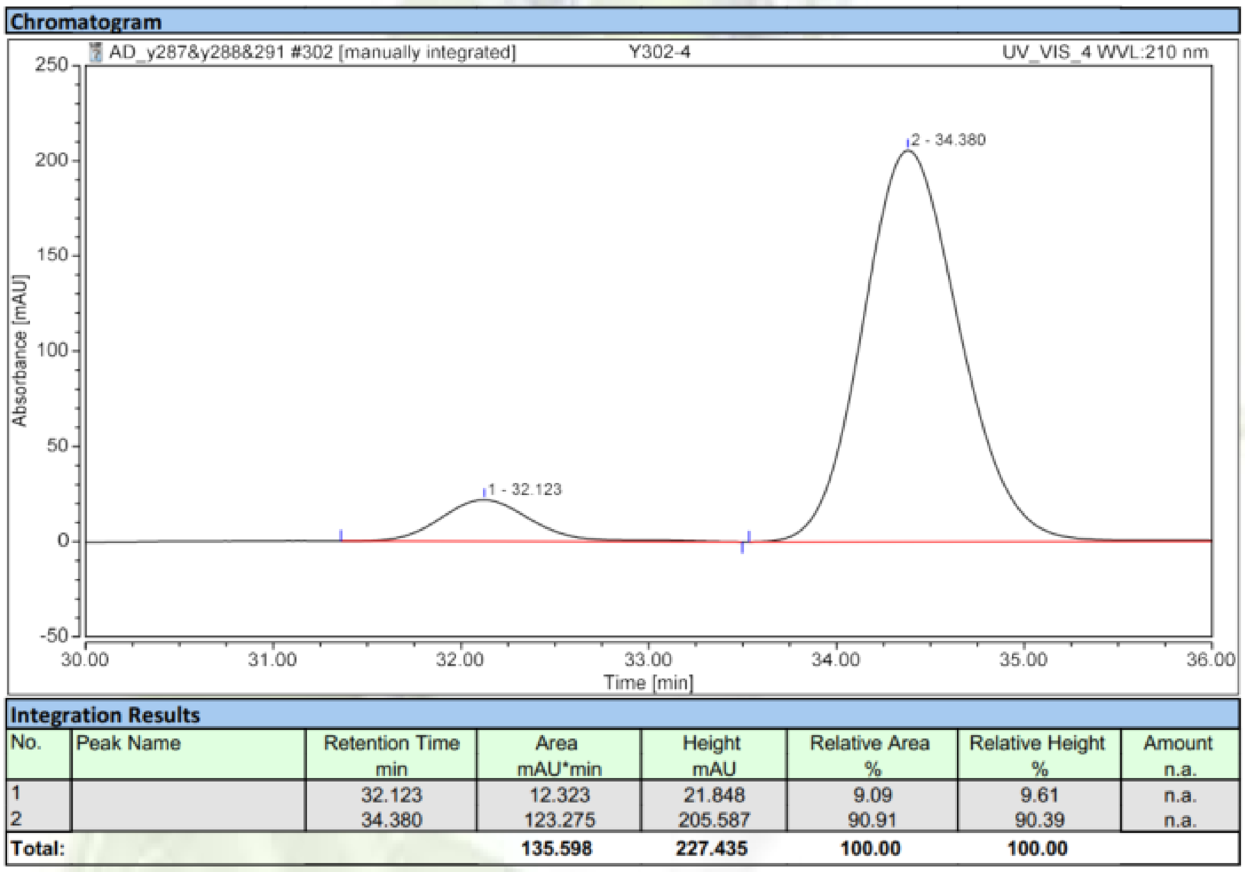

Integration Results

No.	Peak Name	Retention Time min	Area mAU*min	Height mAU	Relative Area %	Relative Height %	Amount n.a.
1		32.123	12.323	21.848	9.09	9.61	n.a.
2		34.380	123.275	205.587	90.91	90.39	n.a.
**Total:**			**135.598**	**227.435**	**100.00**	**100.00**	

THF:H_2O =1:1; %ee = 86%

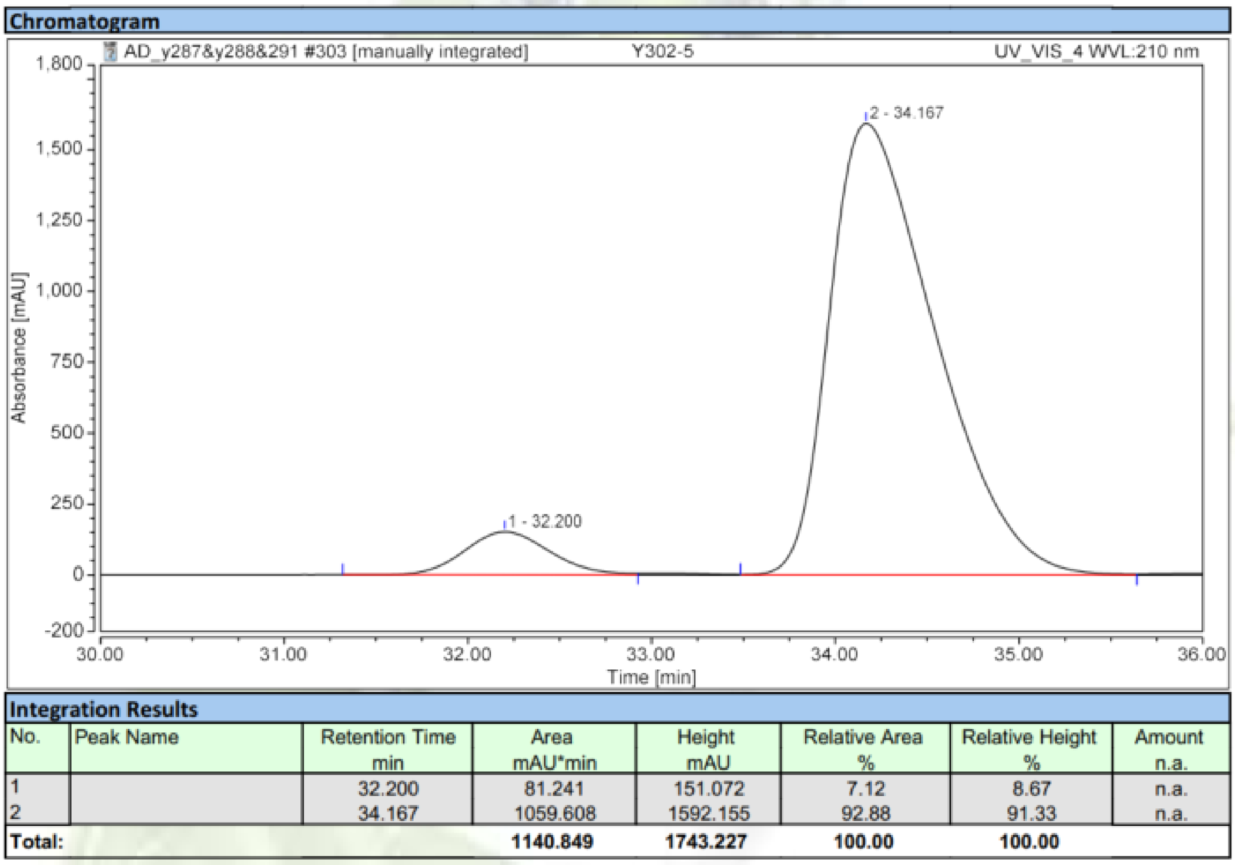

Integration Results

No.	Peak Name	Retention Time min	Area mAU*min	Height mAU	Relative Area %	Relative Height %	Amount n.a.
1		32.200	81.241	151.072	7.12	8.67	n.a.
2		34.167	1059.608	1592.155	92.88	91.33	n.a.
**Total:**			**1140.849**	**1743.227**	**100.00**	**100.00**	

THF:H_2O =1:1.5; %ee = 90%

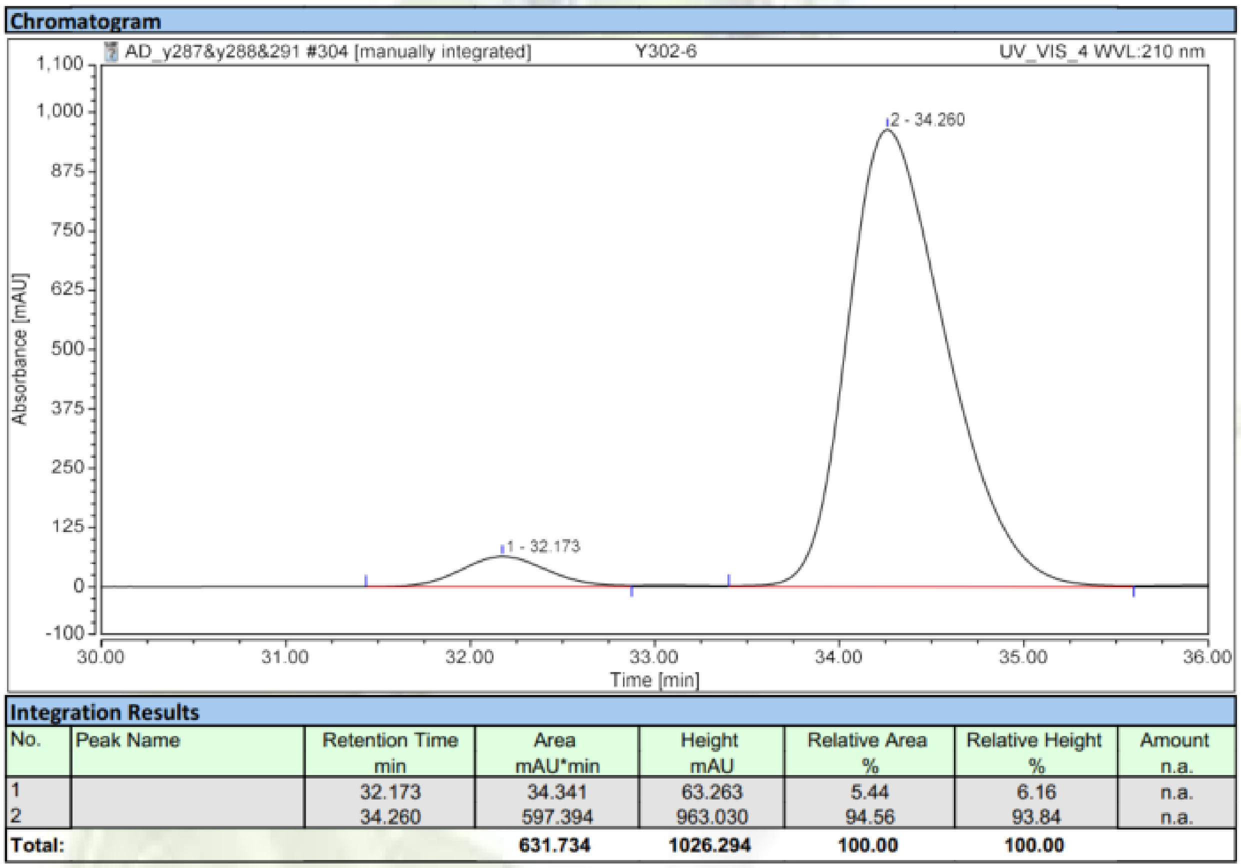

Integration Results

No.	Peak Name	Retention Time min	Area mAU*min	Height mAU	Relative Area %	Relative Height %	Amount n.a.
1		32.173	34.341	63.263	5.44	6.16	n.a.
2		34.260	597.394	963.030	94.56	93.84	n.a.
**Total:**			**631.734**	**1026.294**	**100.00**	**100.00**	

THF:H_2O =1:3; %ee = 94%

Chromatogram

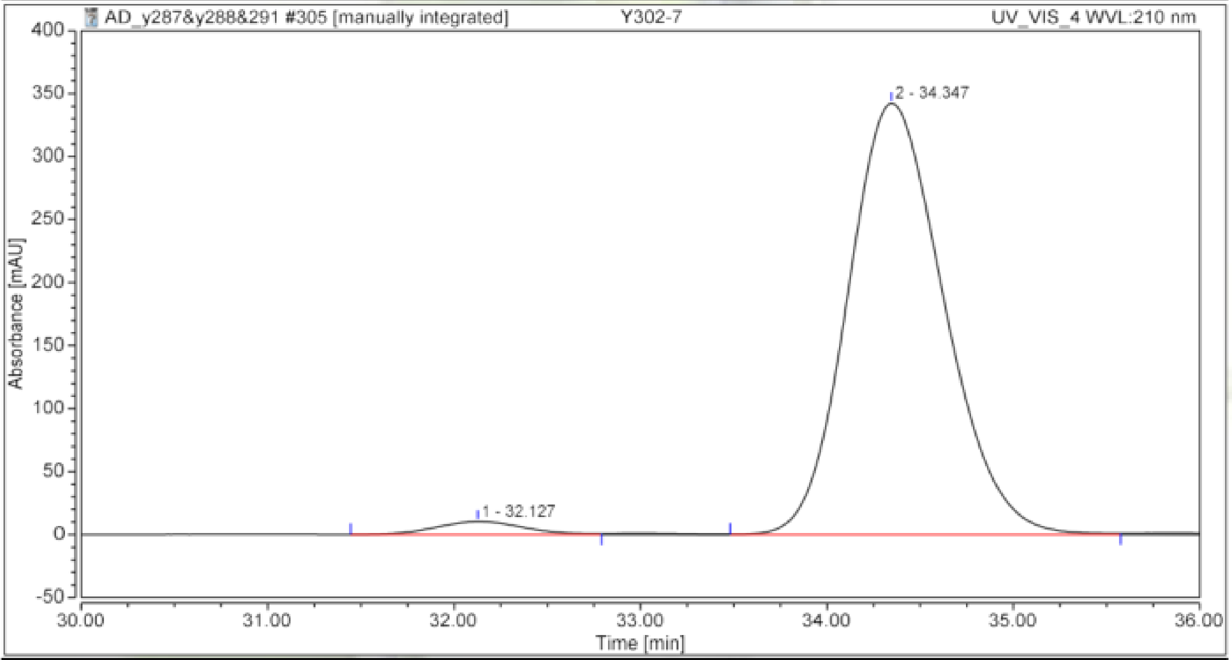

Integration Results

No.	Peak Name	Retention Time min	Area mAU*min	Height mAU	Relative Area %	Relative Height %	Amount n.a.
1		32.127	5.610	10.286	2.67	2.92	n.a.
2		34.347	204.835	342.239	97.33	97.08	n.a.
**Total:**			**210.445**	**352.525**	**100.00**	**100.00**	

THF:H_2O =1:4.5; %ee = 90%

Chromatogram

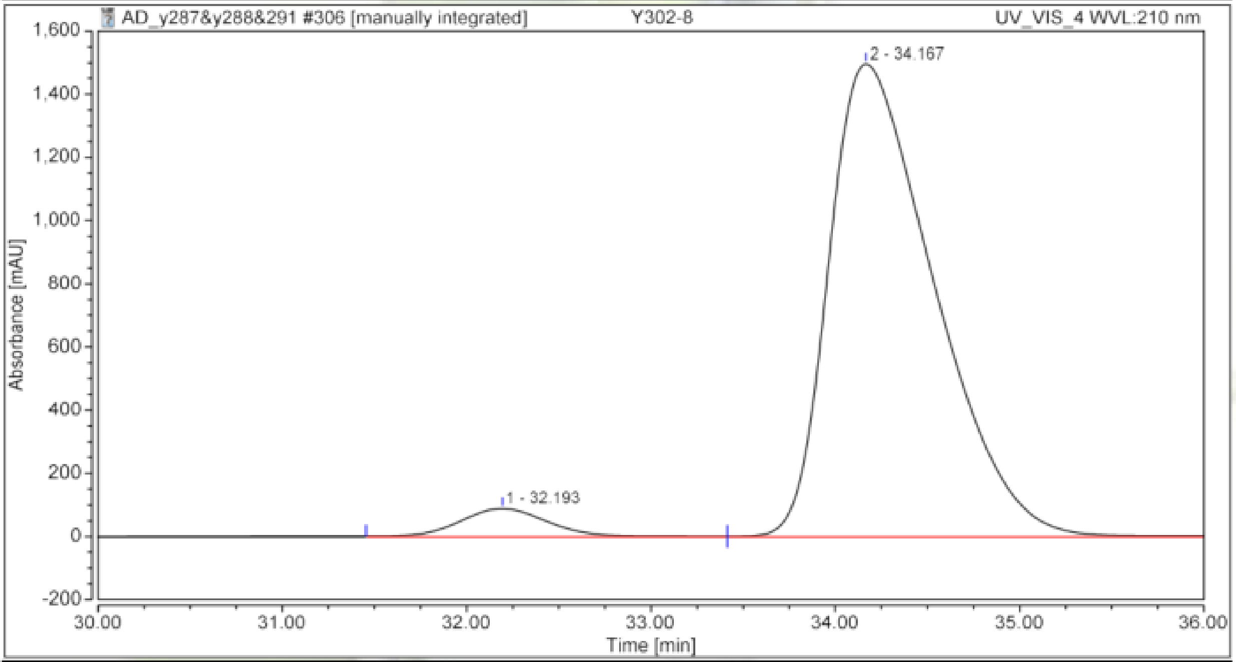

Integration Results

No.	Peak Name	Retention Time min	Area mAU*min	Height mAU	Relative Area %	Relative Height %	Amount n.a.
1		32.193	48.721	88.729	4.69	5.60	n.a.
2		34.167	990.090	1495.657	95.31	94.40	n.a.
**Total:**			**1038.812**	**1584.385**	**100.00**	**100.00**	

THF:H_2O =1:6; %ee = 88%

Chromatogram

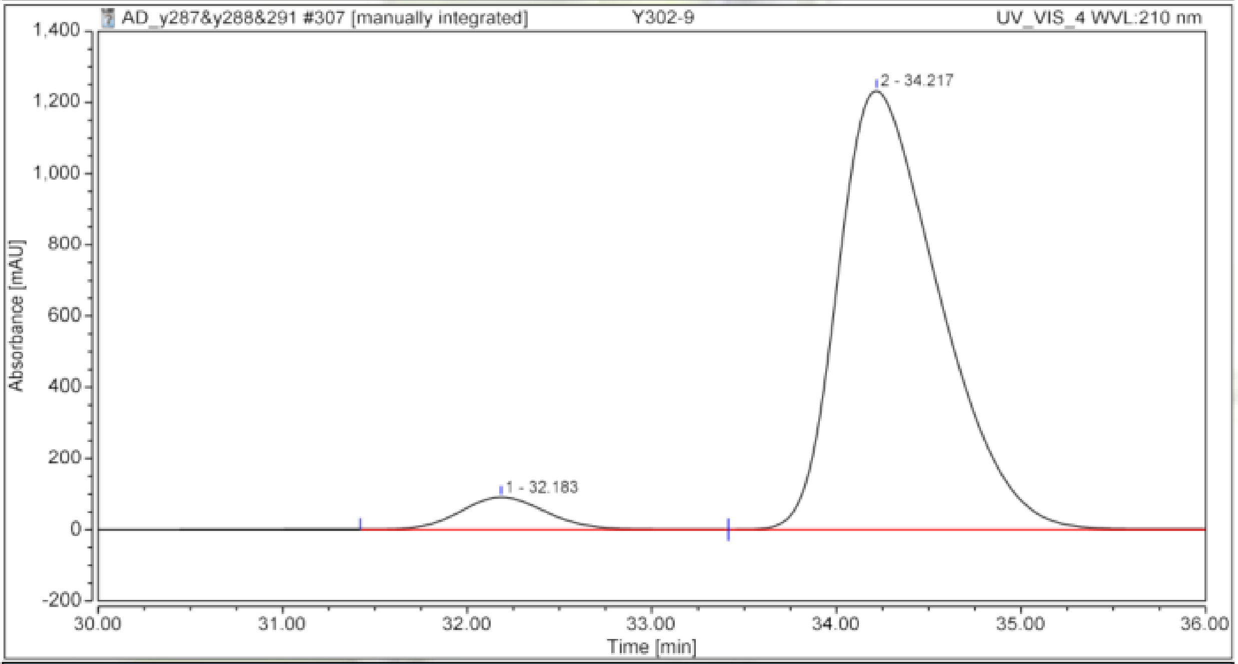

Integration Results

No.	Peak Name	Retention Time min	Area mAU*min	Height mAU	Relative Area %	Relative Height %	Amount n.a.
1		32.183	49.934	90.980	5.98	6.88	n.a.
2		34.217	784.743	1231.995	94.02	93.12	n.a.
**Total:**			**834.677**	**1322.975**	**100.00**	**100.00**	

(1R,2R)-3-methoxy-1-phenylpropane-1,2-diol

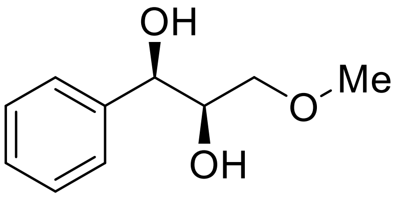

3b

- Column: Chiralpak OJ-H.
- Condition: 5% *i*PrOH/Hexane at 0.5 mL/min, enantiomeric excess determined at 210 nm; 14.3 min (SS), 17.0 min (RR).

THF:H_2O =4.5:1; %ee = 78%

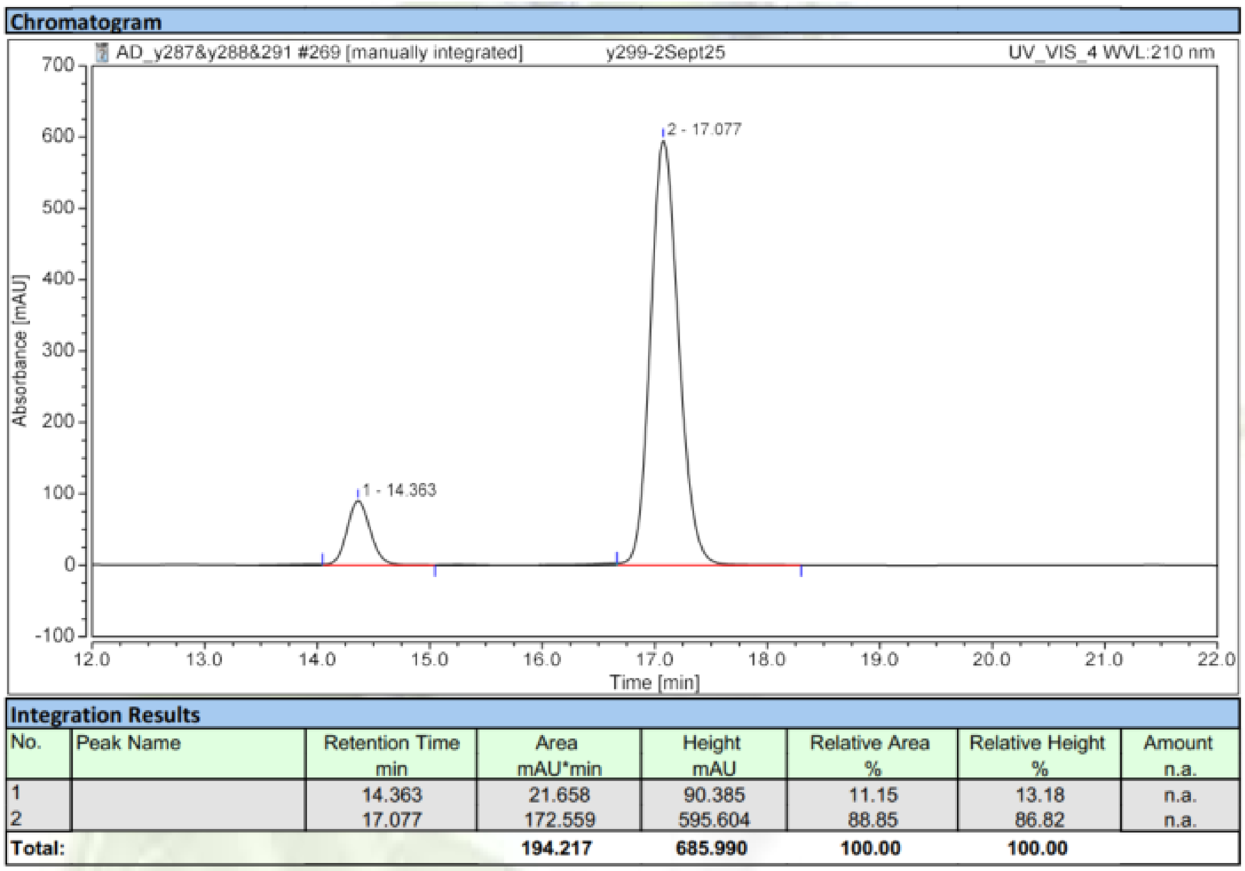

Integration Results

No.	Peak Name	Retention Time min	Area mAU*min	Height mAU	Relative Area %	Relative Height %	Amount n.a.
1		14.363	21.658	90.385	11.15	13.18	n.a.
2		17.077	172.559	595.604	88.85	86.82	n.a.
**Total:**			**194.217**	**685.990**	**100.00**	**100.00**	

THF:H_2O =3:1; %ee = 80%

Chromatogram

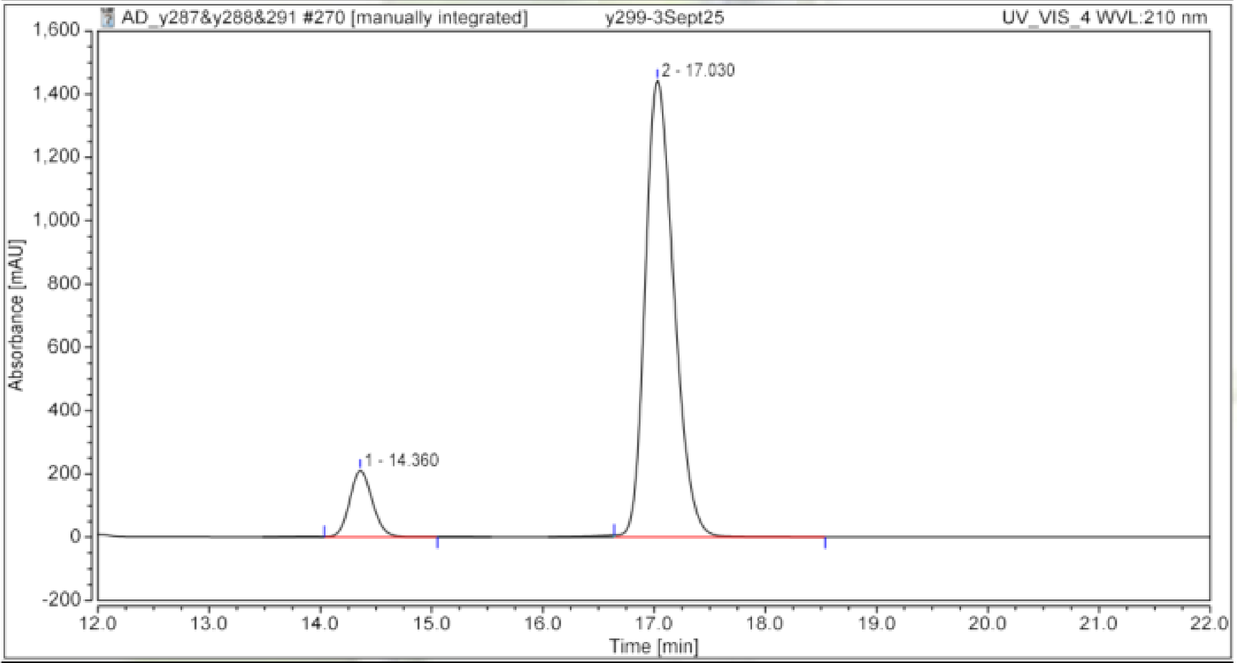

Integration Results

No.	Peak Name	Retention Time min	Area mAU*min	Height mAU	Relative Area %	Relative Height %	Amount n.a.
1		14.360	50.024	210.061	10.39	12.71	n.a.
2		17.030	431.335	1442.874	89.61	87.29	n.a.
**Total:**			**481.359**	**1652.936**	**100.00**	**100.00**	

THF:H_2O =1.5:1; %ee = 84%

Chromatogram

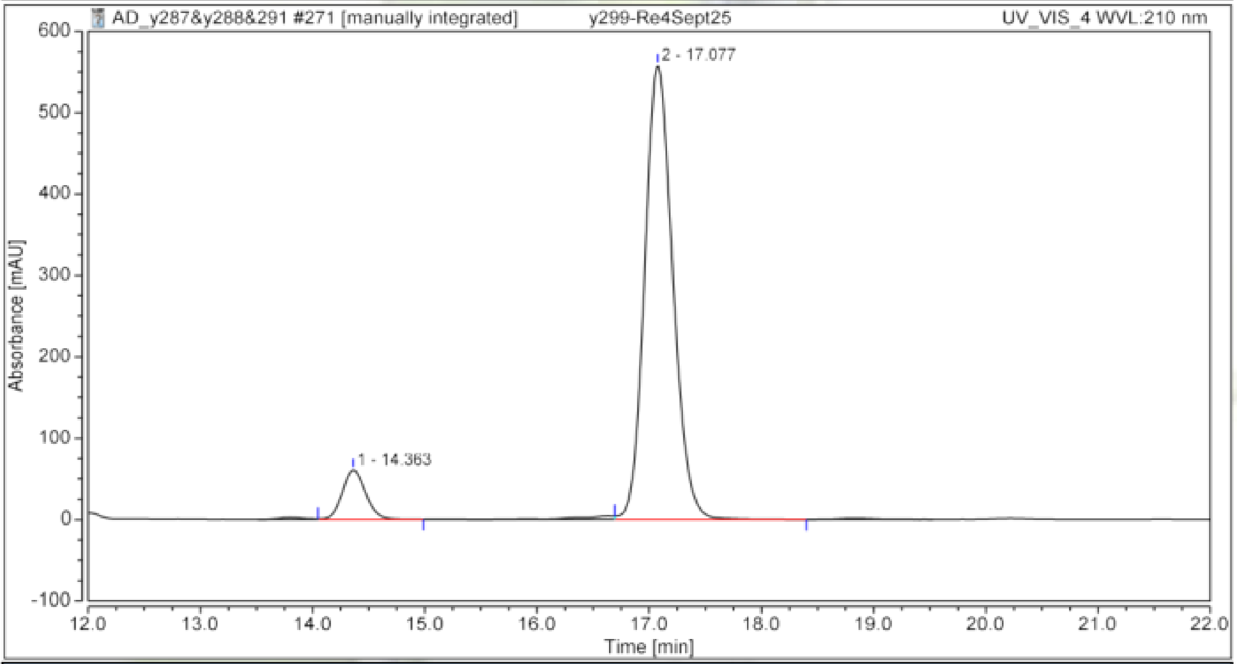

Integration Results

No.	Peak Name	Retention Time<br>min	Area<br>mAU*min	Height<br>mAU	Relative Area<br>%	Relative Height<br>%	Amount<br>n.a.
1		14.363	14.747	60.791	8.35	9.83	n.a.
2		17.077	161.842	557.910	91.65	90.17	n.a.
**Total:**			**176.589**	**618.701**	**100.00**	**100.00**	

THF:H_2O =1:1; %ee = 86%

Chromatogram

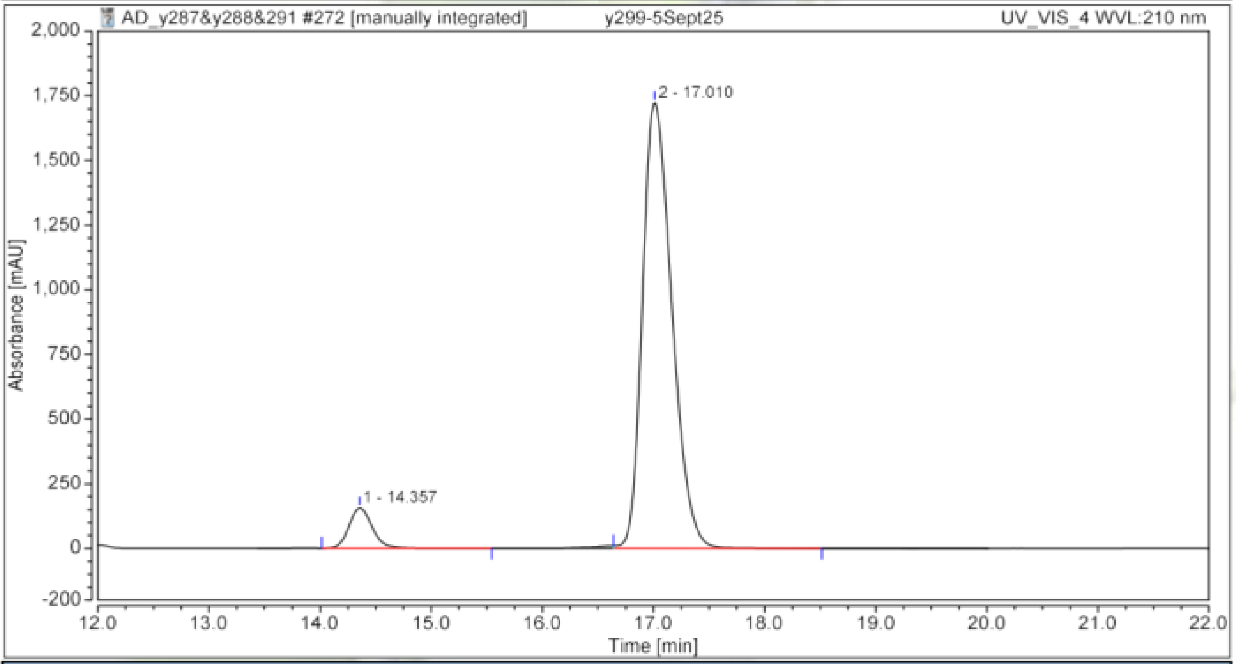

Integration Results

No.	Peak Name	Retention Time min	Area mAU*min	Height mAU	Relative Area %	Relative Height %	Amount n.a.
1		14.357	37.887	156.385	6.70	8.32	n.a.
2		17.010	527.563	1723.291	93.30	91.68	n.a.
**Total:**			**565.451**	**1879.676**	**100.00**	**100.00**	

THF:H_2O =1:1.5; %ee = 90%

Chromatogram

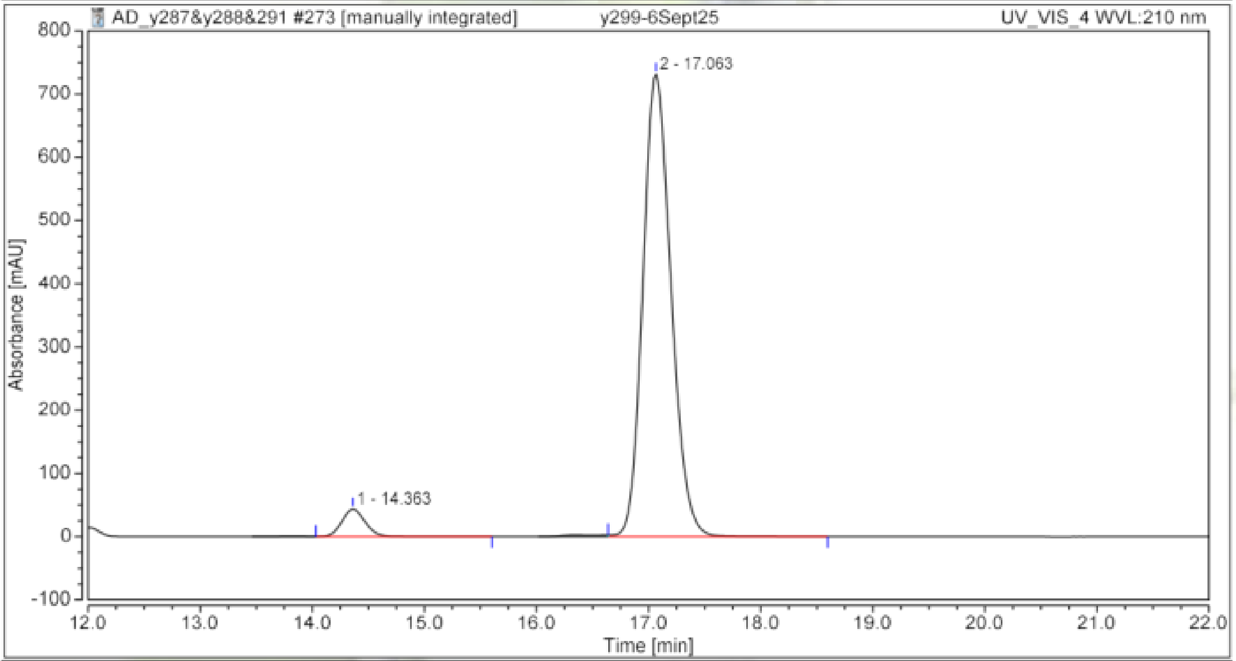

Integration Results

No.	Peak Name	Retention Time min	Area mAU*min	Height mAU	Relative Area %	Relative Height %	Amount n.a.
1		14.363	10.611	43.566	4.75	5.62	n.a.
2		17.063	212.654	731.440	95.25	94.38	n.a.
**Total:**			**223.264**	**775.005**	**100.00**	**100.00**	

THF:H_2O =1:3; %ee = 94%

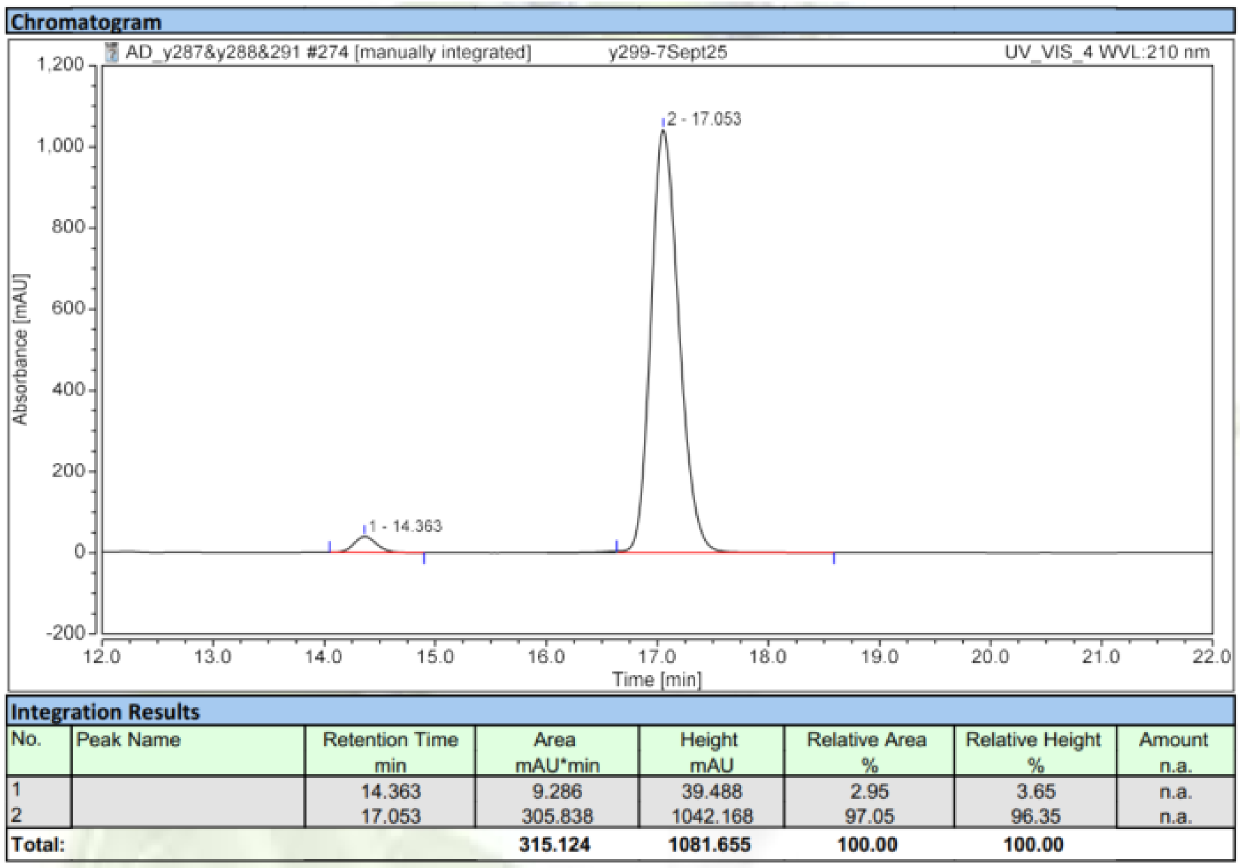

Integration Results

No.	Peak Name	Retention Time min	Area mAU*min	Height mAU	Relative Area %	Relative Height %	Amount n.a.
1		14.363	9.286	39.488	2.95	3.65	n.a.
2		17.053	305.838	1042.168	97.05	96.35	n.a.
**Total:**			**315.124**	**1081.655**	**100.00**	**100.00**	

THF:H_2O =1:4.5; %ee = 94%

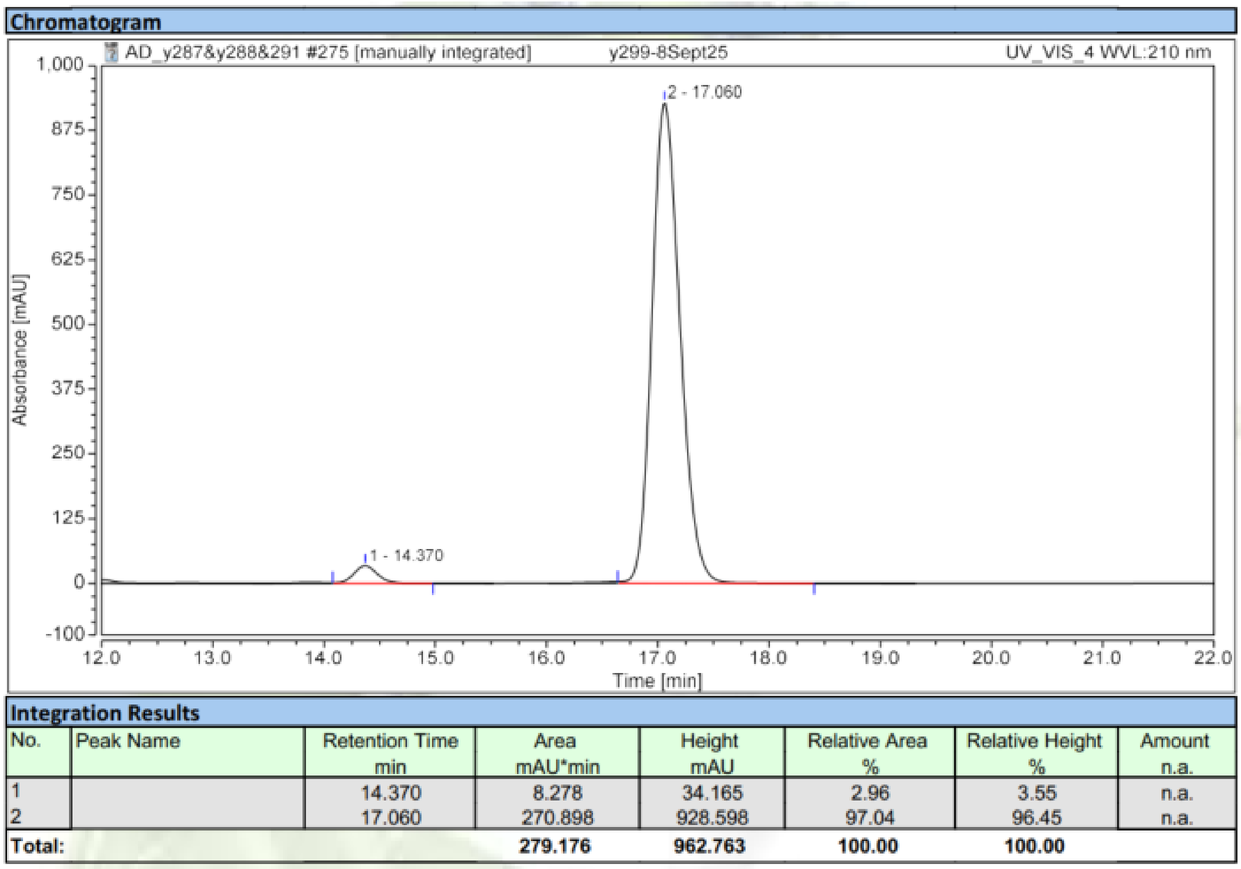

Integration Results

No.	Peak Name	Retention Time min	Area mAU*min	Height mAU	Relative Area %	Relative Height %	Amount n.a.
1		14.370	8.278	34.165	2.96	3.55	n.a.
2		17.060	270.898	928.598	97.04	96.45	n.a.
**Total:**			**279.176**	**962.763**	**100.00**	**100.00**	

THF:H_2O =1:6; %ee = 92%

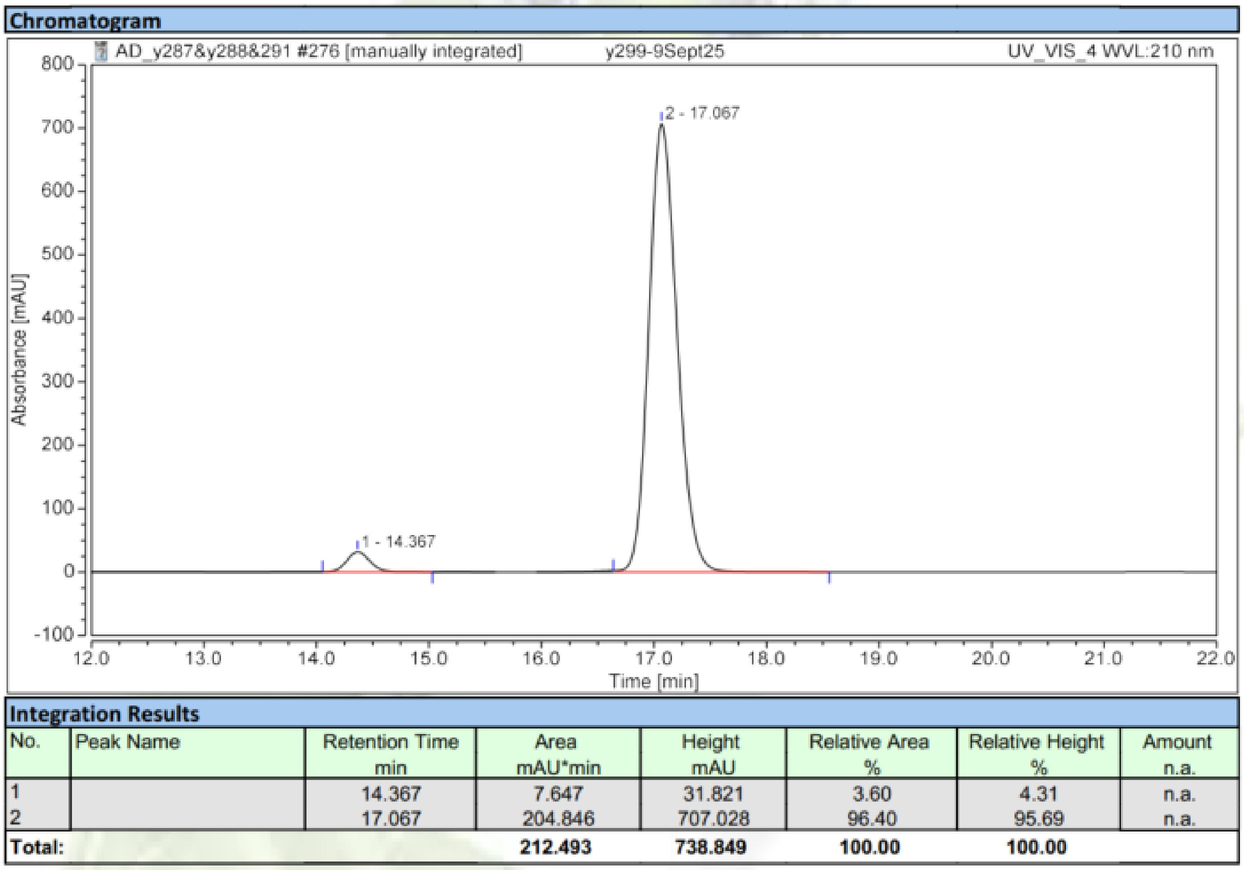

Integration Results

No.	Peak Name	Retention Time min	Area mAU*min	Height mAU	Relative Area %	Relative Height %	Amount n.a.
1		14.367	7.647	31.821	3.60	4.31	n.a.
2		17.067	204.846	707.028	96.40	95.69	n.a.
**Total:**			**212.493**	**738.849**	**100.00**	**100.00**	

5. DLS-based particle size distribution of AD-β

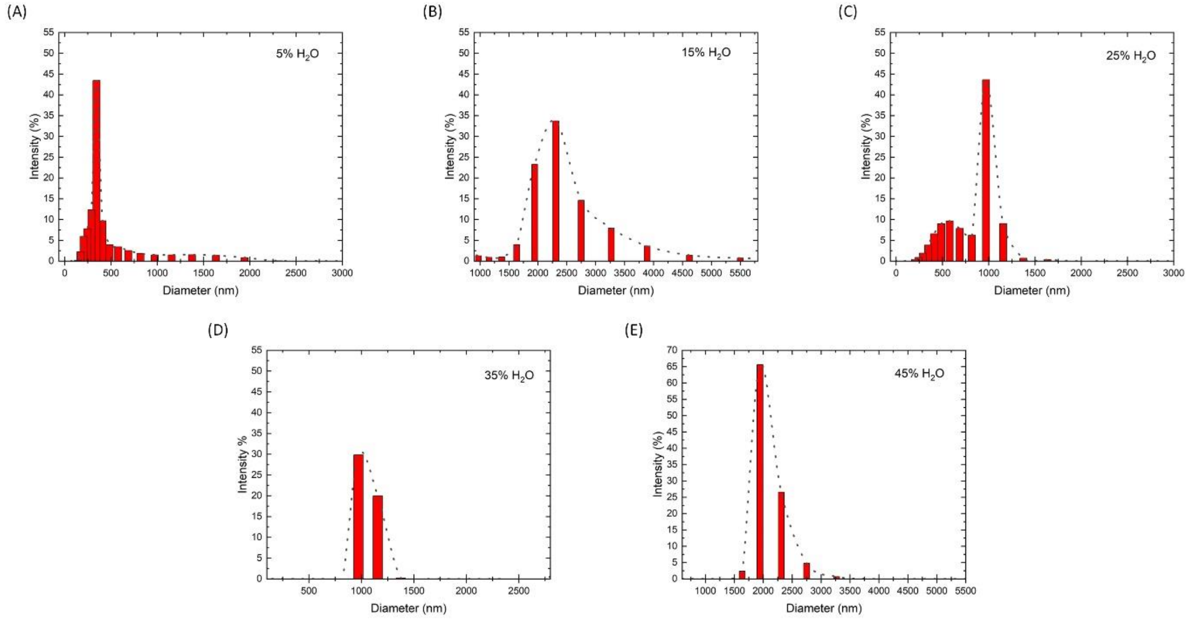

Figure S15. DLS particle size distribution curves of AD-β obtained in THF/Water mixture (5-5 45% H_2O, v/v)

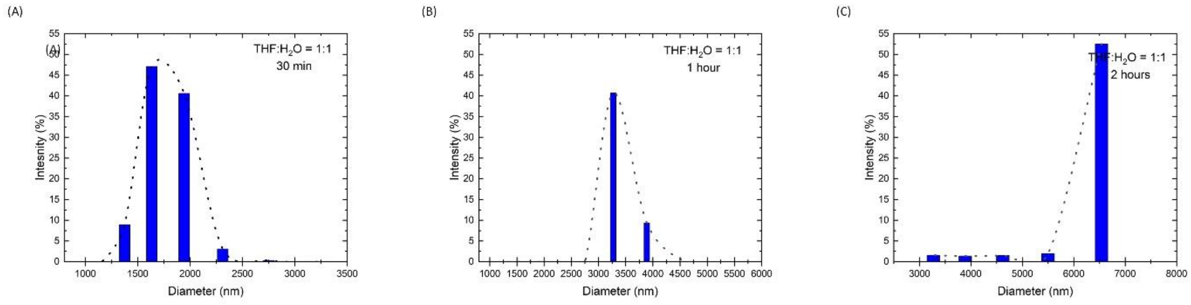

Figure S16. DLS particle size distribution curves of AD-β obtained in THF/Water mixture (1:1 THF: H_2O) at times of 30 min, 1 hour, and 2 hours.

6. Aggregation-induced catalysis on organocatalytic asymmetric Diels-Alder reaction.

The present aggregation-induced asymmetric catalysis strategy demonstrated promising results on other reactions. For example, in the same co-solvent system containing THF and H_2O, organocatalytic asymmetric Diels-Alder reaction between 1,3-diphenylisobenzofuran and (*E*)-but-2-enal depicted opposite enantioselectivity with respect to *endo* isomer by increasing *f*w values. The exclusive endo/exo selectivity was observed under this aggregation system which is different from the reported counterpart using the same catalyst of (S)-5-benzyl-2,2,3-trimethylimidazolidin-4-one [38-41]. The enantioselectivity was unambiguously determined by converting Diels-Alder adducts to corresponding sulfimine diastereoisomers measured by proton NMR. As shown in Figure 5, enantioselectivity was controlled as 0.76:0.24 with the ratio of v-THF: v-H_2O as 3.17:1.0.

However, enantioselectivity was reversed as 0.42: 0.58 when the ratio of v-THF: v-H_2O reached 0.72:1.0. This aggregation strategy would serve as a greener and more environmentally friendly tool of controlling chirality of products as compared with classical methods aforementioned. In the latter, in order to generate products of opposite chirality, the chirality of reactants, as well as catalysts or chiral solvents, needs to be reversed by performing synthesis of these materials, causing manpower and energy and producing more waste generation.

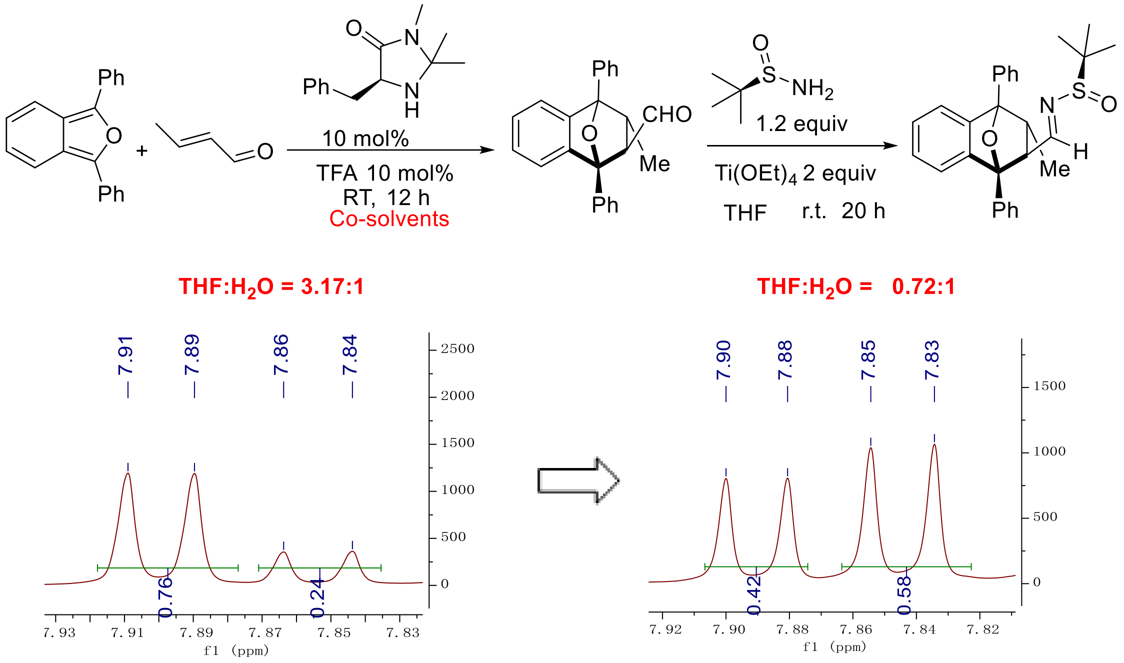

Figure S17. Aggregation-induced catalysis on organocatalytic asymmetric Diels-Alder reaction.

7. Reference:

1. E. K. Aratikatla, T. R. Valkute, S. K. Puri, K. Srivastava, A. K. Bhattacharya, Norepinephrine alkaloids as antiplasmodial agents: Synthesis of syncarpamide and insight into the structure-activity relationships of its analogues as antiplasmodial agents. *Eur. J. Med. Chem.* **138**, 1089–1105 (2017).
2. J. Zhu, X.-T. Sun, X.-D. Wang, L. Wu, Enantioselective dihydroxylation of alkenes catalyzed by 1,4-bis(9-O -dihydroquinidinyl)phthalazine-modified binaphthyl-osmium nanoparticles. *ChemCatChem*. **10**, 1788–1792 (2018).
